# Synthesis and Antibacterial Evaluation of Ciprofloxacin Congeners with Spirocyclic Amine Periphery

**DOI:** 10.3390/ijms24020954

**Published:** 2023-01-04

**Authors:** Alexei Lukin, Kristina Komarova, Lyubov Vinogradova, Elizaveta Rogacheva, Lyudmila Kraeva, Mikhail Krasavin

**Affiliations:** 1 Lomonosov Institute of Fine Chemical Technologies, MIREA—Russian Technological University, Moscow 119454, Russia; 2 Pasteur Institute of Epidemiology and Microbiology, 14 Mira Street, Saint Petersburg 197101, Russia; 3 Institute of Chemistry, Saint Petersburg State University, Saint Petersburg 199034, Russia; 4 Institute for Medicine and Life Sciences, Immanuel Kant Baltic Federal University, Kaliningrad 236016, Russia

**Keywords:** fluoroquinolones, ciprofloxacin, spirocycles, azomethine [3+2] cycloaddition, 1,2,4-triazoles, ESKAPE pathogens

## Abstract

The synthesis of novel fluoroquinolones, congeners of ciprofloxacin, which was inspired by earlier work on spirocyclic ciprofloxacin, is described. An antibacterial evaluation of the 11 fluoroquinolone compounds synthesized against the ESKAPE panel of pathogens in comparison with ciprofloxacin revealed that the more compact spirocycles in the fluoroquinolone periphery resulted in active compounds, while larger congeners gave compounds that displayed no activity at all. In the active cohort, the level of potency was comparable to that of ciprofloxacin. However, the spectrum of antibacterial activity was quite different, as the new compounds showed no activity against *Pseudomonas aeruginosa*. Among the prepared and tested compounds, the broadest range of activity (five pathogens of the six in the ESKAPE panel) and the highest level of activity were demonstrated by 1-yclopropyl-7-[8-(4-cyclopropyl-4*H*-1,2,4-triazol-3-yl)-6-azaspiro[3.4]oct-6-yl]-6-fluoro-4-oxo-1,4-dihydroquinoline-3-carboxylic acid, which is the lead compound nominated for further characterization and development.

## 1. Introduction

Fluoroquinolones represent a distinctly important class of antibiotics, which is effective against both Gram-positive and Gram-negative bacteria [1] with acceptable levels of resistance [2]. The mechanism of action of these antimicrobial compounds involves entering into the cytosol of bacteria, binding to topoisomerase II (DNA gyrase) and IV and inactivating these enzymes. This action, which determines excellent safety profiles of fluoroquinolones in humans, is specific to prokaryotes [3]. Fluoroquinolones have been shown to possess the right physicochemical properties to allow them to penetrate the lipid bilayer of Gram-negative bacteria [4]. Moreover, fluoroquinolones are effective against mycobacteria, which defines their role in the treatment of tuberculosis [5].

In addition to their antibacterial action, fluoroquinolones exert immunomodulatory effects due to their ability to regulate the immune system’s modulators and suppress pro-inflammatory cytokines [6]. Moreover, fluoroquinolones have shown promise as anticancer agents [7].

**Figure 1:**
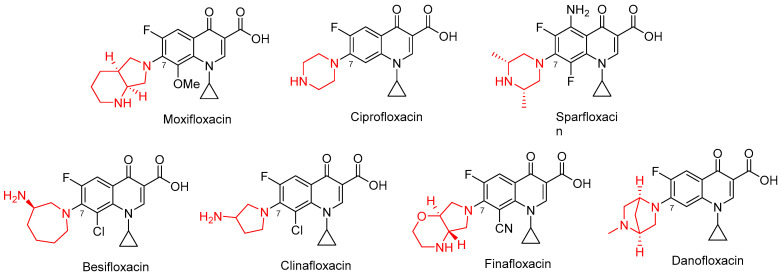
Approved and advanced investigational fluoroquinolones with variable 7-amino group.

Many fluoroquinolones have either achieved the status of advanced research chemical tools or have been approved for clinical use, which allows for classifying them into four generations [8]. Among the periphery elements that vary in different fluoroquinolones, the amine substituent in position 7 is of particular importance [9]. Notable examples illustrating the 7-amino group’s variations in fluoroquinolones, in combination with altering the aromatic ring substituents, include moxifloxacin [10], ciprofloxacin [11], sparfloxacin [12], besifloxacin [13], clinafloxacin [14], finafloxacin [15] and danofloxacin [16] (Figure 1). Apparently, despite the abundance of advanced fluoroquinolones, new drugs of this class with new 7-amino substituents continue to be approved [15,17], which makes bringing new candidate fluoroquinolone molecules up the development pipeline a worthy goal.

While the substituent on the quinolone nitrogen atom in fluoroquinolones can be varied [9], the majority of approved drugs contain the cyclopropyl group in this position. This makes the *N*-cyclopropyl template such as ciprofloxacin a suitable starting point for exploring variations of the 7-amino moiety [11].

**Figure 2:**
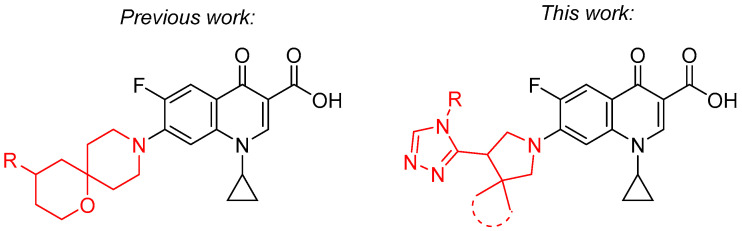
Spirocyclic congeners of ciprofloxacin reported previously and explored in this work.

Previously, we reported a series of 1-oxa-9-azaspiro[5.5]undecane derivatives of ciprofloxacin that demonstrated high antibacterial potency against Gram-negative *Acinetobacter baumannii* 987^®^ and Gram-positive Bacillus cereus 138^®^ [18]. Spirocycles are of special significance for drug discovery [19] as they are conformationally rigid and inherently three-dimensional and possess high F_sp3_ (fraction of sp^3^-hybridized atoms among the total atom count) [20]. Encouraged by our initial success exploring spirocycles, in lieu of the piperazine moiety in ciprofloxacin, and having established the beneficial effect of bulky heterocyclic R group on the 1-oxa-9-azaspiro[5.5]undecane scaffold [18], we embarked upon replacing this motif with a different type of spirocyclic amines, namely spirocyclic pyrrolidines that bear a privileged [21] 1,2,4-triazol-3-yl substituent (Figure 2). In this article, we describe the synthesis of these new spirocyclic congeners of ciprofloxacin and their antibacterial profiles established against the ESKAPE panel of pathogens, i.e., six highly virulent and antibiotic-resistant bacteria [22].

## 2. Results and Discussion

### 2.1. Compound Synthesis

**Scheme 1:**
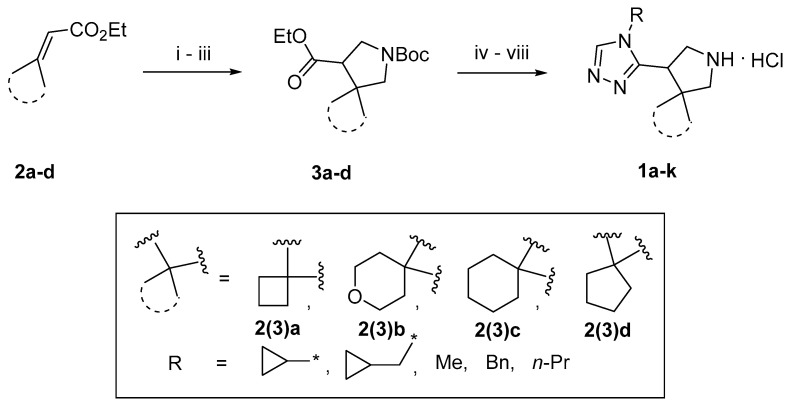
Synthesis of spirocyclic 1,2,4-triazol-3-yl-substituted building blocks **1a–k** (for structures of **1a–k**, see Table 1). *Reagents and conditions:* i. MeOCH_2_N(Bn)CH_2_SiMe_3_, LiF, MeCN, 60 °C, 18 h; ii. H_2_, 10% Pd-C, 100 atm, r. t., 12 h; iii. Boc_2_O, EtOAc, 18 h; iv. 64% aq. N_2_H_4_, ethanol, reflux, 8 h; v. RNCS, ethanol, reflux, 2 h; vi. sat. aq. K_2_CO_3_, reflux, 8 h; vii. Raney Ni, ethanol, reflux, 12 h; viii. HCl, 1,4-dioxane, r.t., 18 h.

The requisite spirocyclic 1,2,4-triazol-3-yl-substituted pyrrolidine building blocks **1a–k** were synthesized as hydrochloride salts from known (ethoxycarbonyl)methylidene starting materials **2a** [23], **2b** [24], **2c** [25] and **2d** [26] as follows. A [3 + 2] cycloaddition with **2a–d** and *N*-(methoxymethyl)-*N*-(trimethylsilylmethyl) benzylamine in the presence of lithium fluoride was established the spirocyclic core. The benzyl group was removed by hydrogenation and replaced with a Boc group afforded intermediates **3a–d**. Synthesis of acyl hydrazides by treatment of **3a–d** with aqueous hydrazine in refluxing ethanol was followed by the reaction with alkyl thioisocyanates, also in refluxing ethanol, to afford intermediates that were cyclized on treatment with potassium carbonate. The resulting 1,2,4-triazole-5-thiones were treated with Raney nickel, which caused them to desulfurize and afford 1,2,4-triazoles Boc-protected at the pyrrolidine nitrogen. The Boc group was removed upon treatment with anhydrous HCl in 1,4-dioxane, to furnish 11 spirocyclic building blocks **1a–k** as hydrochloride salts; see Table 1 for the structures (Scheme 1).

**Scheme 2:**
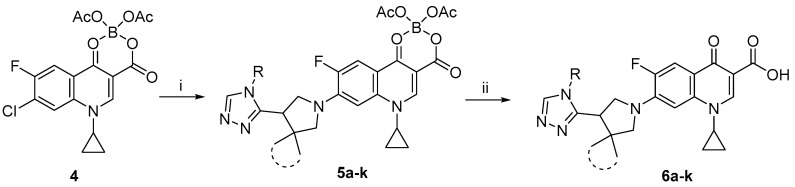
Preparation of ciprofloxacin congeners **6a–k** that bear spirocyclic periphery. *Reagents and conditions:* i. 1, Et_3_N, MeCN, 60 °C, 10 h; ii. 2% aq. NaOH, r. t., 18 h.

Spirocycles **1a–k** were directly used to prepare the ciprofloxacin congeners. To this end, we employed the earlier described [18] boron complex **4** and performed the displacement of the chlorine atom in the latter in the presence of triethylamine. After a brief fractionation on silica gel, intermediate boron complexes **5a–k** were decomposed by treatment with dilute aqueous sodium hydroxide, which furnished 11 ciprofloxacin congeners **6a–k** that bear the spirocyclic periphery (Scheme 2).

### 2.2. Establishment of the Antibacterial Profile of Compounds ***6a–k***

Compounds **6a–k** were evaluated for the susceptibility of the ESKAPE bacteria [22] to their action using the Kirby–Bauer disk diffusion method [27] and, depending on the appearance of the inhibition zone, were then tested by the serial dilution method to evaluate their minimum inhibitory concentration (MIC) in comparison with the values displayed by ciprofloxacin itself. The MIC data are collated in Table 1.

Clearly, the antibacterial activity of the compounds was sensitive to the structure of the spirocyclic periphery. Compounds with their spirocyclic component larger than five-membered (i.e., compounds **6f–6k**) did not display bacterial-growth inhibition in the initial disk diffusion susceptibility test, warranting their progression to MIC testing. A more compact spirocyclic periphery—i.e., 6-azaspiro[3.4]octane (**6a–c**) and 2-azaspiro[4.4]nonane (**6d–e**) resulted in potent antibacterials with activity against some of the ESKAPE pathogens that was comparable to that of ciprofloxacin. However, the spectrum of activity of the active compounds (**6a–e**) was different from that of the clinically used antibiotic. While the potency against *Enterobacter cloacae*, *Staphylococcus aureus* and *Enterococcus faecalis* was in the single-digit mg/mL range, these compounds displayed virtually no activity against *Pseudomonas aeruginosa*. At the same time, the activity against *Klebsiella pneumoniae* and *Acinetobacter baumannii*, although displayed by some of the compounds, turned out to be particularly sensitive to the compound’s periphery. For instance, only cyclopropane-containing azaspiro[3.4]octanes **6a** and **6c** were reasonably active against *Klebsiella pneumoniae*. While most of the compounds in the active cohort were active specifically against *Acinetobacter baumannii* (albeit moderately), *n*-propyl 1.2.4-triazole compound **6b** showed only a weak activity. Notably, all of the compounds in the active cohort compared favorably to a lead structure (**3w**) from our previous study [18], except for MIC vs. *Acinetobacter baumannii*, where the latter compound was exceptionally potent.

Judging by the breadth of the antibacterial activity spectrum and its level being comparable to that of ciprofloxacin, compound **6a** that bear azaspiro[3.4]octane periphery clearly manifested itself as the lead compound active against five of the six pathogens (i.e., all except for *Pseudomonas aeruginosa*) of the ESKAPE panel.

## 3. Materials and Methods

### 3.1. Compound Synthesis

#### 3.1.1. General

All commercial reagents were used without purification. NMR spectra were recorded using a Bruker Avance III spectrometer (Moscow, Russia) in CDCl_3_, *d*_6_-DMSO, D_2_O or 1 M DCl in D_2_O (^1^H: 400.13 MHz; ^13^C: 100.61 MHz); chemical shifts are reported as parts per million (δ, ppm); multiplicities are abbreviated as follows: s = singlet, d = doublet, t = triplet, q = quartet, m = multiplet, br = broad, dd = doublet of doublets, dt = doublet of triplets, and ddd = doublet/doublets of doublets; coupling constants, *J*, are reported in Hz (see Supplementary Materials). Mass spectra were recorded using a Bruker microTOF spectrometer (ionization by electrospray, positive ions detection) (Moscow, Russia). Melting points were determined in open capillary tubes on a Stuart SMP50 Automatic Melting Point Apparatus (Stone, UK). Analytical thin-layer chromatography was carried out on UV-254 silica gel plates using appropriate eluents. Compounds were visualized with short-wavelength UV light. Column chromatography was performed using silica gel Merk grade 60 (0.040−0.063 mm) 230−400 mesh (Darmstadt, Germany). All reactions were conducted in the atmosphere of argon. All compounds were judged to be at least 90% pure based on their high-performance liquid chromatography traces.

#### 3.1.2. 6-*tert*-Butyl 8-ethyl 6-azaspiro[3.4]octane-6,8-dicarboxylate (**3a**)

General Procedure 1 for the preparation of compounds **3a–d**.

To a solution of ethyl cyclobutylideneacetate **2a** (2.9 g, 20.75 mmol, 1 equiv.) in acetonitrile (50 mL), lithium fluoride (2.15 g, 83 mmol, 4 equiv.) and (methoxymethyl)-1-phenyl-N-(trimethylsilylmethyl)methanamine (6.25 g, 25 mmol, 1.2 equiv.) were added. The resulting mixture was stirred at 60 °C overnight. The volatiles were removed in vacuo, the residue was dissolved in ethyl acetate (50 mL), and the solution was washed with sat. aq. citric acid (3 × 25 mL). The combined aqueous solutions were extracted with ethyl acetate (2 × 100 mL), basified to pH 8.0 with sat. aq. K_2_CO_3_ and extracted again with ethyl acetate (2 × 100 mL). The combined organic solutions were dried over anhydrous Na_2_SO_4_, filtered and concentrated in vacuo. The residue was dissolved in ethanol (25 mL). Then, 10% Pd/C (0.25 g) was added, and the mixture was hydrogenated in an autoclave at 100 atm and room temperature for 12 h. The mixture was filtered through a plug of Celite, and the filtrated was concentrated in vacuo. The residue was dissolved in ethyl acetate (50 mL), and Boc_2_O (4.5 g, 20.75 mmol, 1 equiv.) was added in portions. The reaction mixture was attired at room temperature overnight, whereupon it was washed with 5% aq. citric acid (3 × 50 mL), dried over anhydrous Na_2_SO_4_, filtered and concentrated in vacuo. The residue was purified by column chromatography on silica gel using 1% methanol in CH_2_Cl_2_ as eluent to afford 2.87 g (49%) of the title compound as a transparent oil.

^1^H NMR (300 MHz, *d_6_*-DMSO) δ 4.21–4.00 (m, 2H), 3.46–3.26 (m, 4H), 2.94 (dd, *J* = 12.3, 6.3 Hz, 1H), 2.17–1.67 (m, 6H), 1.40 (s, *J* = 21.9 Hz, 9H), 1.21 (t, *J* = 7.1 Hz, 3H); ^13^C NMR (75 MHz, *d_6_*-DMSO) δ 172.06, 171.98, 154.06, 153.94, 78.84, 60.60, 56.95, 56.65, 51.11, 50.42, 47.23, 47.04, 46.89, 46.46, 31.78, 31.44, 28.57, 27.42, 27.31, 15.81, 14.61. LRMS (ESI): *m/z* (M + H) calcd, 284.4; found, 284.2.

#### 3.1.3. 2-*tert*-Butyl 4-ethyl 8-oxa-2-azaspiro[4.5]decane-2,4-dicarboxylate (**3b**)

Prepared according to General Procedure 1 from olefin **2b**. Yield 3.44 g (53%), clear oil. ^1^H NMR (300 MHz, *d_6_*-DMSO) δ 4.21–4.01 (m, 2H), 3.76–3.60 (m, 2H), 3.42 (dt, *J* = 26.0, 10.8 Hz, 5H), 3.20 (dd, *J* = 10.7, 4.1 Hz, 1H), 2.90 (dd, *J* = 8.5, 5.2 Hz, 1H), 1.79 (td, *J* = 13.5, 4.1 Hz, 1H), 1.48 (d, *J* = 9.8 Hz, 1H), 1.40 (s, 9H), 1.39–1.27 (m, 3H), 1.20 (t, *J* = 7.1 Hz, 3H); ^13^C NMR (75 MHz, *d_6_*-DMSO) δ 172.28, 172.06, 154.13, 153.97, 78.77, 60.41, 54.68, 54.29, 51.95, 50.86, 46.85, 45.25, 44.32, 35.38, 35.28, 30.63, 30.51, 28.54, 25.82, 23.29, 23.14, 22.90, 14.51. LRMS (ESI): m/z (M + H) calcd, 314.4; found, 312.2.

#### 3.1.4. 2-*tert*-Butyl 4-ethyl 2-azaspiro[4.4]decane-2,4-dicarboxylate (**3c**)

Prepared according to General Procedure 1 from olefin **2c**. Yield 2.96 g (46%), clear oil. ^1^H NMR (300 MHz, *d_6_*-DMSO) δ 4.23–3.96 (m, 2H), 3.50–3.38 (m, 2H), 3.36–3.22 (m, 1H), 3.15–3.00 (m, 1H), 2.80 (q, *J* = 6.9 Hz, 1H), 1.68–1.44 (m, 5H), 1.43–1.35 (m, 10H), 1.34–1.04 (m, 9H); ^13^C NMR (75 MHz, *d_6_*-DMSO) δ 172.28, 172.06, 154.13, 153.97, 78.77, 60.41, 54.68, 54.29, 51.95, 50.86, 46.85, 45.25, 44.32, 35.38, 35.28, 30.63, 30.51, 28.54, 25.82, 23.29, 23.14, 22.90, 14.51. LRMS (ESI): m/z (M + H) calcd, 312.2; found, 312.2.

#### 3.1.5. 2-*tert*-Butyl 4-ethyl 2-azaspiro[4.4]nonane-2,4-dicarboxylate (**3d**)

Prepared according to General Procedure 1 from olefin **2d**. Yield 3.4 g (56%), clear oil. ^1^H NMR (300 MHz, *d_6_*-DMSO) δ 4.20–3.98 (m, 2H), 3.49–3.39 (m, 2H), 3.32 (s, 1H), 3.08 (dd, *J* = 10.2, 6.1 Hz, 1H), 2.89 (dt, *J* = 17.3, 6.5 Hz, 1H), 2.81–2.58 (m, 1H), 1.80–1.68 (m, 1H), 1.66–1.50 (m, 5H), 1.46–1.29 (m, 11H), 1.19 (t, *J* = 7.1 Hz, 3H); ^13^C NMR (75 MHz, *d_6_*-DMSO) δ 172.98, 172.80, 169.83, 154.03, 153.91, 78.82, 57.22, 56.96, 52.31, 51.89, 51.41, 50.71, 50.05, 47.91, 47.84, 36.66, 36.33, 32.49, 28.54, 24.63, 24.55, 24.39, 24.34. LRMS (ESI): *m/z* (M + H) calcd, 294.4; found, 294.2.

#### 3.1.6. 8-(4-Cyclopropyl-4*H*-1,2,4-triazol-3-yl)-6-azaspiro[3.4]octane hydrochloride (**1a**)

General Procedure 2 for the preparation of compounds **1a–k**.

To a solution of 6-tert-butyl 8-ethyl, 6-azaspiro[3.4]octane-6,8-dicarboxylate (**3a**) (2 g, 7 mmol) in ethanol (15 mL) N_2_H_4_ (64% aqueous solution, 1 mL) was added. The resulting mixture was heated at reflux for 8 h, cooled to room temperature and concentrated in vacuo. The residue was dissolved in ethanol (25 mL), and cyclopropyl thioisocyanate (0.87 g, 8.75 mmol, 1.25 equiv.) was added. The mixture was heated at reflux for 2 h and cooled down to room temperature. Sat. aq. K_2_CO_3_ (5 mL) was added, and the mixture was brought to reflux again and was stirred at that temperature for 8 h. Upon cooling to room temperature, the mixture was concentrated in vacuo. The residue was dissolved in water (25 mL), and the solution was neutralized with 5% aq. HCl. The resulting precipitate was separated by filtration and dissolved in ethanol (25 mL). A suspension of freshly prepared Raney nickel in a minimum amount of ethanol was added, and the resulting mixture was heated at reflux for 12 h. Upon cooling to room temperature, the mixture was filtered through a plug of Celite, and the filtrate was concentrated in vacuo. The residue was fractionated by column chromatography on silica gel using 1% methanol in CH_2_Cl_2_ as eluent. Fractions containing the product were pooled and concentrated in vacuo. The residue was dissolved in 1,4-dioxane (5 mL), and 4M solution of HCl in 1,4-dioxane (5 mL) was added. The solution was stirred at room temperature overnight and concentrated in vacuo. The residue was crystallized from ethanol.

Yield 1.1 g (62%), white solid, m.p. 121–123 °C. ^1^H NMR (300 MHz, *d_6_*-DMSO) δ 10.14 (s, 1H), 9.73 (s, *J* = 28.2 Hz, 1H), 9.61 (s, 1H), 3.99 (t, *J* = 6.0 Hz, 1H), 3.72–3.54 (m, 2H), 3.54–3.30 (m, 3H), 2.29–2.03 (m, 2H), 1.99–1.79 (m, 3H), 1.78–1.56 (m, 1H), 1.31–1.05 (m, 4H); ^13^C NMR (75 MHz, *d_6_*-DMSO) δ 154.84, 144.29, 55.37, 54.20, 49.03, 48.63, 47.61, 31.92, 27.34, 26.46, 15.82, 7.17, 6.87; LRMS (ESI): *m/z* (M + H) calcd, 219.3; found, 219.4.

#### 3.1.7. 8-(4-Propyl-4*H*-1,2,4-triazol-3-yl)-6-azaspiro[3.4]octane hydrochloride (**1b**)

Prepared according to General Procedure 2 from ester **3a** and *n*-propyl thioisocyanate. Yield 0.88 g (49%), white solid, m.p. 128–129 °C. ^1^H NMR (300 MHz, *d_6_*-DMSO) δ 10.26 (s, 1H), 9.79 (s, 2H), 4.25–4.12 (m, 2H), 3.99 (dd, *J* = 7.2, 5.4 Hz, 1H), 3.65–3.26 (m, 4H), 2.21–2.01 (m, 2H), 1.97–1.75 (m, 5H), 1.74–1.49 (m, 1H), 0.92 (t, *J* = 7.3 Hz, 3H); ^13^C NMR (75 MHz, *d_6_*-DMSO) δ 153.33, 144.06, 55.37, 54.06, 48.72, 47.62, 47.14, 31.44, 26.25, 23.28, 15.77, 11.01; LRMS (ESI): *m/z* (M + H) calcd, 221.3; found, 221.2.

#### 3.1.8. 8-[4-(Cyclopropylmethyl)-4*H*-1,2,4-triazol-3-yl]-6-azaspiro[3.4]octane hydrochloride (**1c**)

Prepared according to General Procedure 2 from ester **3a** and (cyclopropyl)methyl thioisocyanate. Yield 0.82 g (44%), white solid, m.p. 103–104 °C. ^1^H NMR (300 MHz, *d_6_*-DMSO) δ 10.30 (s, 1H), 10.02–9.68 (m, 2H), 4.13 (d, *J* = 7.0 Hz, 2H), 4.00 (t, *J* = 6.0 Hz, 1H), 3.51–3.28 (m, 3H), 2.19–1.99 (m, 3H), 1.95–1.75 (m, 3H), 1.76–1.51 (m, *J* = 4.5 Hz, 1H), 1.50–1.16 (m, *J* = 12.3 Hz, 1H), 0.70–0.41 (m, 4H);^13^C NMR (75 MHz, *d_6_*-DMSO) δ 153.33, 144.06, 55.37, 54.06, 48.72, 47.62, 47.14, 31.44, 26.25, 23.28, 15.77, 11.01. LRMS (ESI): *m/z* (M + H) calcd, 233.3; found, 233.4.

#### 3.1.9. 4-(4-Methyl-4*H*-1,2,4-triazol-3-yl)-2-azaspiro[4.4]nonane hydrochloride (**1d**)

Prepared according to General Procedure 2 from ester **3d** and methyl thioisocyanate. Yield 1.05 g (62%), white solid, m.p. 150–151 °C. ^1^H NMR (300 MHz, *d_6_*-DMSO) δ 10.27 (s, 1H), 9.93 (s, 1H), 9.73 (s, 1H), 3.94–3.86 (m, 1H), 3.83 (s, 3H), 3.73–3.40 (m, 2H), 3.34–3.05 (m, 2H), 1.97–1.75 (m, 1H), 1.48 (tdd, *J* = 33.8, 26.4, 7.5 Hz, 8H); ^13^C NMR (75 MHz, *d_6_*-DMSO) δ 154.18, 144.64, 54.28, 54.12, 48.19, 35.78, 33.15, 31.13, 24.14, 23.99; LRMS (ESI): *m/z* (M + H) calcd, 207.3; found, 207.4.

#### 3.1.10. 4-(4-Cyclopropyl-4*H*-1,2,4-triazol-3-yl)-2-azaspiro[4.4]nonane hydrochloride (**1e**)

Prepared according to General Procedure 2 from ester **3d** and cyclopropyl thioisocyanate. Yield 1.01 g (54%), white solid, m.p. 135–136 °C. ^1^H NMR (300 MHz, *d_6_*-DMSO) δ 10.23 (s, 1H), 9.95 (s, 1H), 9.79 (s, 1H), 3.93 (s, 1H), 3.80–3.42 (m, 3H), 3.37–3.06 (m, 2H), 1.94–1.78 (m, *J* = 11.6 Hz, 1H), 1.77–1.36 (m, 7H), 1.33–0.99 (m, *J* = 37.2 Hz, 4H); ^13^C NMR (75 MHz, *d_6_*-DMSO) δ 155.26, 144.16, 54.43, 53.98, 48.30, 41.14, 36.24, 31.20, 27.69, 24.28, 24.14, 7.28, 6.73; LRMS (ESI): *m/z* (M + H) calcd, 233.3; found, 233.2.

#### 3.1.11. 4-(4-Methyl-4*H*-1,2,4-triazol-3-yl)-2-azaspiro[4.5]decane hydrochloride (**1f**)

Prepared according to General Procedure 2 from ester **3c** and methyl thioisocyanate. Yield 1.06 g (59%), white solid, m.p. 147–148 °C. ^1^H NMR (300 MHz, *d_6_*-DMSO) δ 10.17 (s, 1H), 9.84 (s, 1H), 9.51 (s, 1H), 3.80 (s, *J* = 18.5 Hz, 3H), 3.73–3.47 (m, *J* = 14.5 Hz, 3H), 3.43–3.28 (m, 1H), 3.26–3.10 (m, 1H), 1.83 (d, *J* = 11.3 Hz, 1H), 1.65–1.18 (m, 7H), 1.16–0.61 (m, 2H); ^13^C NMR (75 MHz, *d_6_*-DMSO) δ 152.88, 145.12, 55.37, 51.44, 47.13, 46.99, 42.69, 34.37, 32.80, 29.84, 25.31, 23.40, 22.69; LRMS (ESI): *m/z* (M + H) calcd, 221.3; found, 221.4.

#### 3.1.12. 4-[4-(Cyclopropylmethyl)-4*H*-1,2,4-triazol-3-yl]-2-azaspiro[4.5]decane hydrochloride (**1g**)

Prepared according to General Procedure 2 from ester **3c** and (cyclopropyl)methyl thioisocyanate. Yield 1.07 g (52%), white solid, m.p. 136–137 °C. ^1^H NMR (300 MHz, *d_6_*-DMSO) δ 10.28 (s, 1H), 9.87 (s, 1H), 9.60 (s, 1H), 4.08–3.86 (m, *J* = 21.8 Hz, 3H), 3.76–3.46 (m, 3H), 3.42–3.08 (m, 2H), 1.83 (d, *J* = 11.0 Hz, 1H), 1.63–1.17 (m, 9H), 1.12–0.85 (m, *J* = 23.5 Hz, 2H), 0.66–0.41 (m, 4H); ^13^C NMR (75 MHz, *d_6_*-DMSO) δ 152.34, 143.91, 55.39, 51.33, 50.34, 47.14, 42.70, 34.22, 29.91, 25.34, 23.33, 22.76, 11.00, 4.82, 4.41; LRMS (ESI): *m/z* (M + H) calcd, 261.4; found, 261.2.

#### 3.1.13. 4-(4-Benzyl-4*H*-1,2,4-triazol-3-yl)-2-azaspiro[4.5]decane hydrochloride (**1h**)

Prepared according to General Procedure 2 from ester **3c** and benzyl thioisocyanate. Yield 0.97 g (42%), white solid, m.p. 164–165 °C. ^1^H NMR (300 MHz, CDCl_3_) δ 10.06 (s, 1H), 9.71 (s, 1H), 9.29 (s, 1H), 7.46–7.15 (m, 5H), 5.57–5.24 (m, 2H), 3.70–3.54 (m, 2H), 3.52–3.34 (m, *J* = 23.1 Hz, 1H), 3.31–3.15 (m, 2H), 1.72 (d, *J* = 8.7 Hz, 1H), 1.53–1.34 (m, *J* = 6.0 Hz, 2H), 1.34–1.00 (m, 6H), 0.96–0.76 (m, 1H), 0.73–0.49 (m, *J* = 23.6 Hz, 1H); ^13^C NMR (75 MHz, CDCl_3_) δ 144.74, 141.67, 129.45, 128.99, 128.55, 51.44, 48.46, 47.85, 47.24, 47.11, 46.61, 42.58, 34.12, 31.74, 30.16, 25.29, 22.99, 22.85. LRMS (ESI): *m/z* (M + H) calcd, 297.4; found, 297.2.

#### 3.1.14. 4-(4-Methyl-4*H*-1,2,4-triazol-3-yl)-8-oxa-2-azaspiro[4.5]decane hydrochloride (**1i**)

Prepared according to General Procedure 2 from ester **3b** and methyl thioisocyanate. Yield 1.04 g (58%), white solid, m.p. 158–159 °C. ^1^H NMR (300 MHz, *d_6_*-DMSO) δ 10.28 (s, 1H), 10.00 (s, 1H), 9.61 (s, 1H), 3.83 (s, 3H), 3.78–3.47 (m, 6H), 3.46–3.27 (m, *J* = 11.3 Hz, 3H), 1.82 (d, *J* = 12.7 Hz, 1H), 1.75–1.57 (m, *J* = 14.8 Hz, 1H), 1.44–1.15 (m, *J* = 4.3 Hz, 2H); ^13^C NMR (75 MHz, *d_6_*-DMSO) δ 152.74, 145.05, 64.49, 64.06, 50.80, 46.73, 45.01, 42.31, 33.96, 32.99, 30.02; LRMS (ESI): *m/z* (M + H) calcd, 223.3; found, 223.2.

#### 3.1.15. 4-(4-Cyclopropyl-4*H*-1,2,4-triazol-3-yl)-8-oxa-2-azaspiro[4.5]decane hydrochloride (**1j**)

Prepared according to General Procedure 2 from ester **3b** and cyclopropyl thioisocyanate. Yield 0.71 g (36%), white solid, m.p. 145–146 °C. ^1^H NMR (300 MHz, *d_6_*-DMSO) δ 10.25 (s, 1H), 10.03 (s, 1H), 9.67 (s, 1H), 3.93–3.10 (m, 7H), 1.92–1.60 (m, 3H), 1.24 (dt, *J* = 91.8, 38.8 Hz, 8H); ^13^C NMR (75 MHz, *d_6_*-DMSO) δ 153.84, 144.62, 66.80, 64.60, 64.12, 51.09, 46.92, 44.84, 34.56, 30.41, 7.46, 6.83; LRMS (ESI): *m/z* (M + H) calcd, 249.3; found, 249.4.

#### 3.1.16. 4-[4-(Cyclopropylmethyl)-4*H*-1,2,4-triazol-3-yl]-8-oxa-2-azaspiro[4.5]decane hydrochloride (**1k**)

Prepared according to General Procedure 2 from ester **3b** and (cyclopropyl)methyl thioisocyanate. Yield 1.06 g (51%), white solid, m.p. 129–130 °C. ^1^H NMR (300 MHz, *d_6_*-DMSO) δ 10.42 (s, 1H), 10.04 (s, 1H), 9.69 (s, 1H), 4.22–3.88 (m, 2H), 3.85–3.24 (m, 9H), 1.82 (d, *J* = 12.6 Hz, 1H), 1.64 (td, *J* = 12.8, 4.7 Hz, 1H), 1.42–1.15 (m, *J* = 42.7 Hz, 3H), 0.69–0.36 (m, 4H); ^13^C NMR (75 MHz, *d_6_*-DMSO) δ 152.17, 143.90, 64.43, 64.12, 55.41, 50.67, 50.43, 46.86, 45.03, 42.32, 33.81, 30.05, 10.97, 4.84, 4.41; LRMS (ESI): *m/z* (M + H) calcd, 263.4; found, 263.4.

#### 3.1.17. 1-Cyclopropyl-7-[8-(4-cyclopropyl-4*H*-1,2,4-triazol-3-yl)-6-azaspiro[3.4]oct-6-yl]-6-fluoro-4-oxo-1,4-dihydroquinoline-3-carboxylic acid (**6a**)

General procedure 3 for the preparation of compounds **6a–k**.

Compound **4** (98 mg, 0.24 mmol) [18] was dissolved in acetonitrile (10 mL) and treated, with stirring, with spirocyclic amine **1a** (122 mg, 0.48 mmol) and triethylamine (66 μL, 0.48 mmol). The stirring continued at 60 °C for 10 h. The volatiles were removed in vacuo. The residue was fractionated on a silica gel column eluted with 0 → 20% methanol in dichloromethane. Fractions containing the product (by TLC analysis) were pooled and concentrated in vacuo. The residue was dissolved in 2% aqueous NaOH and left to stir at r. t. overnight. The reaction mixture was acidified with 5% aqueous citric acid to pH 4–5. The resulting precipitate was filtered off, washed with water and air-dried.

Yield 38 mg (35%), white solid, m.p. 201–202 °C. ^1^H NMR (300 MHz, *d_6_*-DMSO) δ 15.48 (s, 1H), 8.56 (s, 1H), 8.43 (s, 1H), 7.80 (d, *J* = 14.2 Hz, 1H), 7.08 (d, *J* = 7.3 Hz, 1H), 4.04 (d, *J* = 9.4 Hz, 2H), 3.94–3.62 (m, *J* = 9.9 Hz, 4H), 3.52–3.39 (m, 1H), 2.14 (t, *J* = 7.1 Hz, 2H), 2.03–1.68 (m, 4H), 1.33–0.98 (m, 8H); ^13^C NMR (75 MHz, D_2_O) δ 178.53, 168.98, 155.20, 154.24, 150.98, 150.05, 146.59, 144.33, 144.18, 142.56, 117.22, 117.13, 113.59, 113.28, 108.86, 103.05, 62.88, 56.15, 50.10, 47.71, 38.37, 34.37, 29.03, 26.31, 18.16, 13.37, 10.16. HRMS (ESI) *m/z* calcd for C_25_H_27_FN_5_O_3_ [M + H^+^] 464.2097, found 464.2102.

#### 3.1.18. 1-Cyclopropyl-6-fluoro-4-oxo-7-[8-(4-propyl-4*H*-1,2,4-triazol-3-yl)-6-azaspiro[3.4]oct-6-yl]-1,4-dihydroquinoline-3-carboxylic acid (**6b**)

Prepared according to General Procedure 3 from spirocyclic amine **1b**. Yield 47 mg (43%), white solid, m.p. 189–190 °C. ^1^H NMR (300 MHz, CDCl_3_) δ 15.54 (s, 1H), 8.51 (s, *J* = 14.0 Hz, 1H), 7.79 (d, *J* = 14.1 Hz, 1H), 7.07 (d, *J* = 7.3 Hz, 1H), 4.14–3.91 (m, *J* = 7.1 Hz, 4H), 3.91–3.63 (m, 4H), 2.13–2.01 (m, 1H), 1.96–1.83 (m, 2H), 1.81–1.62 (m, 2H), 1.34–1.08 (m, 4H), 0.87 (t, *J* = 7.3 Hz, 3H); ^13^C NMR (75 MHz, D_2_O) δ 178.53, 168.98, 155.20, 154.24, 150.98, 150.05, 146.59, 144.33, 144.18, 142.56, 117.22, 117.13, 113.59, 113.28, 108.86, 103.05, 62.88, 56.15, 50.10, 47.71, 38.37, 34.37, 29.03, 26.31, 18.16, 13.37, 10.16; HRMS (ESI) *m/z* calcd for C_25_H_29_FN_5_O_3_ [M + H^+^] 464.2254, found 464.2250.

#### 3.1.19. 1-Cyclopropyl-7-{8-[4-(cyclopropylmethyl)-4*H*-1,2,4-triazol-3-yl]-6-azaspiro[3.4]oct-6-yl}-6-fluoro-4-oxo-1,4-dihydroquinoline-3-carboxylic acid (**6c**)

Prepared according to General Procedure 3 from spirocyclic amine **1c**. Yield 25 mg (22%), white solid, m.p. 172–173 °C. ^1^H NMR (300 MHz, D_2_O∙DCl) δ 8.77 (s, 1H), 8.12 (d, *J* = 14.5 Hz, 1H), 7.13 (d, *J* = 13.6 Hz, 1H), 3.50–3.34 (m, *J* = 7.4 Hz, 3H), 3.33–3.14 (m, 4H), 3.05 (s, 1H), 1.38–1.26 (m, 2H), 1.23–0.89 (m, *J* = 8.7 Hz, 4H), 0.68–0.32 (m, *J* = 61.3 Hz, 5H), −0.05 (d, *J* = 7.6 Hz, 2H), −0.31 (s, 2H); ^13^C NMR (75 MHz, D_2_O∙DCl) δ 169.00, 167.82, 154.29, 153.52, 150.16, 148.25, 144.31, 144.15, 142.18, 141.11, 111.00, 110.88, 110.54, 110.22, 102.28, 60.06, 52.79, 52.15, 47.55, 41.33, 37.81, 32.73, 26.14, 15.69, 9.30, 7.86, 4.52, 4.34; HRMS (ESI) *m/z* calcd for C_26_H_29_FN_5_O_3_ [M + H^+^] 478.2254, found 478.2251.

#### 3.1.20. 1-Cyclopropyl-6-fluoro-7-[4-(4-methyl-4*H*-1,2,4-triazol-3-yl)-2-azaspiro[4.4]non-2-yl]-4-oxo-1,4-dihydroquinoline-3-carboxylic acid (**6d**)

Prepared according to General Procedure 3 from spirocyclic amine **1d**. Yield 35 mg (33%), white solid, m.p. 205–206 °C. ^1^H NMR (300 MHz, D_2_O∙DCl) δ 8.57 (s, 1H), 8.14 (s, 1H), 7.12 (d, *J* = 13.1 Hz, 1H), 3.49 (s, 1H), 3.34 (s, 1H), 3.13 (s, 3H), 3.09–2.82 (m, 4H), 1.03–0.46 (m, *J* = 6.7 Hz, 10H), 0.46–0.31 (m, 2H); ^13^C NMR (75 MHz, D_2_O∙DCl) δ 166.04, 165.05, 164.98, 152.41, 150.71, 147.34, 145.40, 141.31, 141.15, 140.62, 138.19, 108.35, 108.22, 107.48, 99.40, 57.30, 51.07, 50.38, 38.25, 34.96, 34.38, 31.12, 28.63, 21.07, 5.01; HRMS (ESI) *m/z* calcd for C_24_H_22_FN_5_O_3_ [M + H^+^] 452.2097, found 452.2100.

#### 3.1.21. 1-Cyclopropyl-7-[4-(4-cyclopropyl-4*H*-1,2,4-triazol-3-yl)-2-azaspiro[4.4]non-2-yl]-6-fluoro-4-oxo-1,4-dihydroquinoline-3-carboxylic acid (**6e**)

Prepared according to General Procedure 3 from spirocyclic amine **1e**. Yield 32 mg (28%), white solid, m.p. 188–189 °C. ^1^H NMR (300 MHz, D_2_O∙DCl) δ 8.59 (s, 1H), 8.11 (d, *J* = 19.6 Hz, 2H), 7.11 (d, *J* = 12.8 Hz, 1H), 3.53 (s, 1H), 3.29 (d, *J* = 32.0 Hz, 2H), 3.09 (s, 3H), 2.77 (s, 1H), 1.08–0.30 (m, 19H); ^13^C NMR (75 MHz, D_2_O∙DCl) δ 166.16, 165.73, 165.02, 158.51, 154.02, 151.34, 145.42, 142.67, 140.57, 138.27, 113.98, 108.17, 100.34, 99.39, 98.45, 50.45, 43.65, 38.78, 34.96, 28.80, 25.59, 21.35, 9.93, 5.09, 4.31, 4.06, −10.57; HRMS (ESI) *m/z* calcd for C_26_H_29_FN_5_O_3_ [M + H^+^] 477.2254, found 477.2257.

#### 3.1.22. 1-Cyclopropyl-6-fluoro-7-[4-(4-methyl-4*H*-1,2,4-triazol-3-yl)-2-azaspiro[4.5]dec-2-yl]-4-oxo-1,4-dihydroquinoline-3-carboxylic acid (**6f**)

Prepared according to General Procedure 3 from spirocyclic amine **1f**. Yield 49 mg (44%), white solid, m.p. 228–229 °C. ^1^H NMR (300 MHz, D_2_O∙DCl) δ 8.53 (s, 1H), 8.16 (s, 1H), 7.17 (d, *J* = 13.4 Hz, 1H), 3.38 (s, 2H), 3.14 (s, 3H), 3.11–2.92 (m, *J* = 30.1 Hz, 4H), 2.33 (s, 2H), 1.03–0.09 (m, 17H); ^13^C NMR (75 MHz, D_2_O∙DCl) δ 166.01, 165.17, 151.26, 145.50, 141.00, 138.11, 108.57, 99.51, 54.54, 49.89, 43.75, 39.91, 35.00, 32.01, 31.26, 27.17, 21.85, 19.91, 19.54, 5.02; HRMS (ESI) *m/z* calcd for C_25_H_29_FN_5_O_3_ [M + H^+^] 466.2254, found 466.2253.

#### 3.1.23. 1-Cyclopropyl-7-{4-[4-(cyclopropylmethyl)-4*H*-1,2,4-triazol-3-yl]-2-azaspiro[4.5]dec-2-yl}-6-fluoro-4-oxo-1,4-dihydroquinoline-3-carboxylic acid (**6g**)

Prepared according to General Procedure 3 from spirocyclic amine **1g**. Yield 50 mg (42%), white solid, m.p. 201–202 °C. ^1^H NMR (300 MHz, D_2_O∙DCl) δ 8.73 (s, 1H), 8.18 (s, *J* = 14.4 Hz, 1H), 7.18 (d, *J* = 12.8 Hz, 1H), 3.60–3.48 (m, 1H), 3.46–3.23 (m, 4H), 3.18–2.91 (m, 4H), 1.07–0.91 (m, 1H), 0.86–0.21 (m, 16H), −0.03 (d, *J* = 7.6 Hz, 2H), −0.22–−0.37 (m, 2H); ^13^C NMR (75 MHz, D_2_O∙DCl) δ 166.19, 165.30, 165.14, 156.51, 150.97, 147.58, 145.54, 142.67, 139.66, 138.30, 99.57, 54.57, 50.13, 49.58, 43.75, 40.20, 35.09, 33.05, 32.13, 27.44, 22.04, 20.00, 19.78, 6.51, 5.15, 1.89, 1.55; HRMS (ESI) *m/z* calcd for C_28_H_33_FN_5_O_3_ [M + H^+^] 506.2567, found 506.2571.

#### 3.1.24. 7-[4-(4-Benzyl-4*H*-1,2,4-triazol-3-yl)-2-azaspiro[4.5]dec-2-yl]-1-cyclopropyl-6-fluoro-4-oxo-1,4-dihydroquinoline-3-carboxylic acid (**6h**)

Prepared according to General Procedure 3 from spirocyclic amine **1h**. Yield 24 mg (19%), white solid, m.p. 250–252 °C. ^1^H NMR (300 MHz, D_2_O∙DCl) δ 8.75 (s, 1H), 8.30 (s, 1H), 7.37–7.09 (m, 1H), 6.96–6.52 (m, 5H), 5.17–4.57 (m, 2H), 3.54–2.76 (m, 5H), 1.21–−0.25 (m, *J* = 227.7 Hz, 15H); ^13^C NMR (75 MHz, D_2_O∙DCl) δ 215.98, 166.06, 164.97, 151.76, 145.43, 141.35, 140.06, 138.13, 128.34, 127.02, 126.78, 126.10, 123.13, 123.09, 122.97, 108.11, 99.39, 54.57, 50.35, 48.30, 43.53, 40.07, 35.12, 31.95, 27.58, 27.32, 27.06, 21.86, 19.76, 5.22; HRMS (ESI) *m/z* calcd for C_31_H_33_FN_5_O_3_ [M + H^+^] 542.2567, found 542.2563.

#### 3.1.25. 1-Cyclopropyl-6-fluoro-7-[4-(4-methyl-4*H*-1,2,4-triazol-3-yl)-8-oxa-2-azaspiro[4.5]dec-2-yl]-4-oxo-1,4-dihydroquinoline-3-carboxylic acid (**6i**)

Prepared according to General Procedure 3 from spirocyclic amine **1i**. Yield 50 mg (45%), white solid, m.p. 224–225 °C. ^1^H NMR (300 MHz, D_2_O∙DCl) δ 8.74 (s, 1H), 8.19 (s, 1H), 7.18 (d, *J* = 12.6 Hz, 1H), 3.65–3.37 (m, *J* = 41.5 Hz, 2H), 3.33–3.01 (m, *J* = 34.8 Hz, 9H), 3.05–2.74 (m, 2H), 1.23–1.00 (m, 2H), 0.97–0.76 (m, 1H), 0.72–0.57 (m, 3H), 0.53–0.39 (m, 2H); ^13^C NMR (75 MHz, D_2_O∙DCl) δ 166.14, 165.22, 151.04, 147.38, 145.47, 141.53, 140.82, 138.31, 108.36, 107.90, 107.58, 99.51, 61.87, 53.34, 49.44, 40.74, 39.44, 35.11, 31.39, 31.10, 27.01, 5.13; HRMS (ESI) *m/z* calcd for C_24_H_27_FN_5_O_4_ [M + H^+^] 468.2047, found 468.2050.

#### 3.1.26. 1-Cyclopropyl-7-[4-(4-cyclopropyl-4*H*-1,2,4-triazol-3-yl)-8-oxa-2-azaspiro[4.5]dec-2-yl]-6-fluoro-4-oxo-1,4-dihydroquinoline-3-carboxylic acid (**6j**)

Prepared according to General Procedure 3 from spirocyclic amine **1j**. Yield 45 mg (38%), white solid, m.p. 211–212 °C. ^1^H NMR (300 MHz, D_2_O∙DCl) δ 8.70 (s, 1H), 8.16 (s, 1H), 7.16 (d, *J* = 13.3 Hz, 1H), 3.58 (s, 1H), 3.42–2.76 (m, 11H), 1.10 (s, 2H), 0.95–0.29 (m, 11H); ^13^C NMR (75 MHz, D_2_O∙DCl) δ 166.09, 165.17, 152.31, 150.69, 147.33, 145.44, 141.47, 141.32, 140.71, 138.28, 108.22, 107.88, 99.47, 61.92, 53.56, 49.70, 40.70, 39.72, 35.09, 31.42, 27.12, 25.69, 5.11, 4.43, 4.17; HRMS (ESI) *m/z* calcd for C_26_H_29_FN_5_O_4_ [M + H^+^] 494.2203, found 494.2200.

#### 3.1.27. 1-Cyclopropyl-7-{4-[4-(cyclopropylmethyl)-4*H*-1,2,4-triazol-3-yl]-8-oxa-2-azaspiro[4.5]dec-2-yl}-6-fluoro-4-oxo-1,4-dihydroquinoline-3-carboxylic acid (**6k**)

Prepared according to General Procedure 3 from spirocyclic amine **1k**. Yield 50 mg (41%), white solid, m.p. 208–209 °C. ^1^H NMR (300 MHz, D_2_O∙DCl) δ 8.86 (s, 1H), 8.16 (s, 1H), 7.16 (d, *J* = 13.3 Hz, 1H), 3.62–3.46 (m, 1H), 3.44–2.99 (m, *J* = 60.8 Hz, 10H), 2.97–2.66 (m, 2H), 1.06 (s, 2H), 0.87–0.29 (m, *J* = 33.3 Hz, 8H), −0.03 (d, *J* = 7.4 Hz, 2H), −0.29 (s, 2H); ^13^C NMR (75 MHz, D_2_O∙DCl) δ 166.03, 165.13, 159.71, 150.46, 145.39, 141.27, 139.30, 138.22, 124.77, 107.54, 99.41, 61.82, 53.26, 49.45, 40.56, 39.53, 35.03, 31.05, 27.03, 6.40, 5.04, 1.80, 1.42; HRMS (ESI) *m/z* calcd for C_27_H_31_FN_5_O_4_ [M + H^+^] 508.2360, found 508.2358.

### 3.2. Biology Studies

Testing was conducted against the following microorganisms: *Enterococcus faecalis* (ATCC 29812), *Staphylococcus aureus* (ATCC 25912), *Klebsiella pneumoniae* (ATCC 19882), *Acinetobacter baumannii* (948^®^, patient-derived strain from the Pasteur Institute own collection), *Pseudomonas aeruginosa* (ATCC 27853) and Enterobacter cloacae (ATCC 13047) for compounds **6a–k** as well as ciprofloxacin (employed as a positive control) using the Kirby–Bauer disk diffusion test [27] under the Standard Operating Procedure of the European Committee on Antimicrobial Susceptibility Testing (EUCAST) [28]. Paper disks bearing 5 mg of the tested compounds and ciprofloxacin were used. Solutions of compounds **6a–k** made up in DMSO (1 mg/10 mL) were prepared and diluted to a total volume of 1 mL with deionized water. Aliquots of the resulting solutions (5 µL each) were added to a Petri dish containing Muller–Hilton agar that was inoculated with a bacterial suspension (McFarland OD ¼ 0.5). After the compound solution had dried off, the Petri dish was incubated at 37 °C for 18 h. The bacterial-growth inhibition zone diameter around the disc with ciprofloxacin or the compounds’ dried solution circular spot indicated the general susceptibility to the drug being assessed. Thereupon, minimum inhibitory concentrations (MIC, µg/mL) were determined using serial broth dilutions [29]. All measurements were completed in triplicate.

## 4. Conclusions

We have presented the synthesis of novel fluoroquinolones, congeners of ciprofloxacin that bear spirocyclic periphery. The synthesis of spirocyclic amines was achieved in several steps from readily available cyclic α,β-unsaturated esters via an azomethine ylide dipolar [3 + 2] cycloaddition, followed by a periphery manipulation to install *N^4^*-alkyl-substituted 1,2,4-triazole periphery. Antibacterial evaluation of the 11 fluoroquinolone compounds synthesized against the ESKAPE panel of pathogens in comparison with ciprofloxacin revealed an interesting structure–activity relationship trend. The more compact spirocycles in the fluoroquinolone periphery (i.e., compounds that bear azaspiro[3.4]octanes and 2-azaspiro[4.4]nonanes as substituents) were antibacterially active, while larger congeners (compounds with 2-azaspiro[4.5]decane and 8-oxa-2-azaspiro[4.5]decane periphery) displayed no activity at all. In the active cohort, the level of potency was comparable to that of ciprofloxacin. However, the spectrum of antibacterial activity was quite different. Among the prepared and tested compounds, the broadest range of activity (five pathogens of the six in the ESKAPE panel) and the highest level of activity were demonstrated by 1-cyclopropyl-7-[8-(4-cyclopropyl-4*H*-1,2,4-triazol-3-yl)-6-azaspiro[3.4]oct-6-yl]-6-fluoro-4-oxo-1,4-dihydroquinoline-3-carboxylic acid (compound **6a**), which is the lead compound nominated for further characterization and development. The results of these studies will be reported in due course.

## Figures and Tables

**Table 1:** Antibacterial activity as minimal inhibitory concentration (MIC, µg/mL) of compounds **6a–k** as well as ciprofloxacin (positive control) against the ESKAPE panel of pathogens (E1 = *Enterobacter cloacae* (G−), S = *Staphylococcus aureus* (G+), K = *Klebsiella pneumoniae* (G−), A = *Acinetobacter baumannii* (G−), P = *Pseudomonas aeruginosa* (G−), and E2 = *Enterococcus faecalis* (G+)); NT—not tested (the disk diffusion inhibition zone diameter did not warrant MIC determination). The MIC values are mean from three different assays (errors were in the range of ±5–10% of the reported values). Compound **3w** from [18] is provided as a comparison. The antibacterial activity of the lead compound (**6a**) is underscored.

Compound	Structure	MIC (μg/mL)					
		E1	S	K	A	P	E2
**6a**	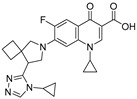	3.0	1.5	2.5	6.0	750	3.0
**6b**	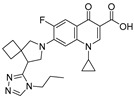	6.0	1.5	>750	100	>750	6.0
**6c**	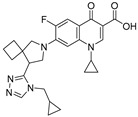	3.0	1.5	12.0	12.0	750	3.0
**6d**	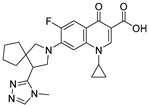	3.0	1.5	190	12.0	>750	1.5
**6e**	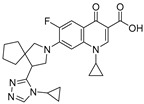	3.0	1.5	190	6.0	750	1.5
**6f**	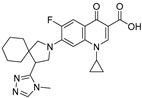	NT	NT	NT	NT	NT	NT
**6g**	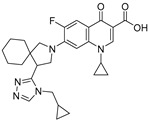	NT	NT	NT	NT	NT	NT
**6h**	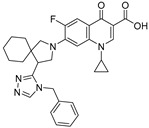	NT	NT	NT	NT	NT	NT
**6i**	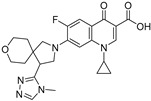	NT	NT	NT	NT	NT	NT
**6j**	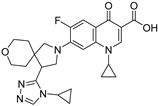	NT	NT	NT	NT	NT	NT
**6k**	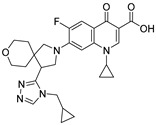	NT	NT	NT	NT	NT	NT
**3w** [18]	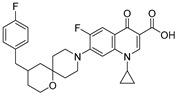	NT	inactive	inactive	0.15	inactive	NT
ciprofloxacin	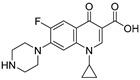	1.25	1.25	0.6	2.5	0.6	0.3

## Data Availability

Not applicable.

## Supplementary Materials

The supporting information can be downloaded at: https://www.mdpi.com/article/10.3390/ijms24020954/s1.
