# Supplementary material for: Synthesis and Antibacterial Evaluation of Ciprofloxacin Congeners with Spirocyclic Amine Periphery
Source: Int J Mol Sci. 2023 Jan 4;24(2):954. doi: 10.3390/ijms24020954 (PMC9863982; doi:10.3390/ijms24020954)
Supplement: Supplementary file 1 [file ijms-24-00954-s001.zip › ijms-2149109-supplementary.pdf]

Supplementary Information for

Contents:

Copies of ^{1}H and ^{13}C NMR spectra S2-S27

Copies of HPLC traces (LCMS) of compounds **6** S28-S33

^{1}H and ^{13}C NMR spectra of compound **3a**

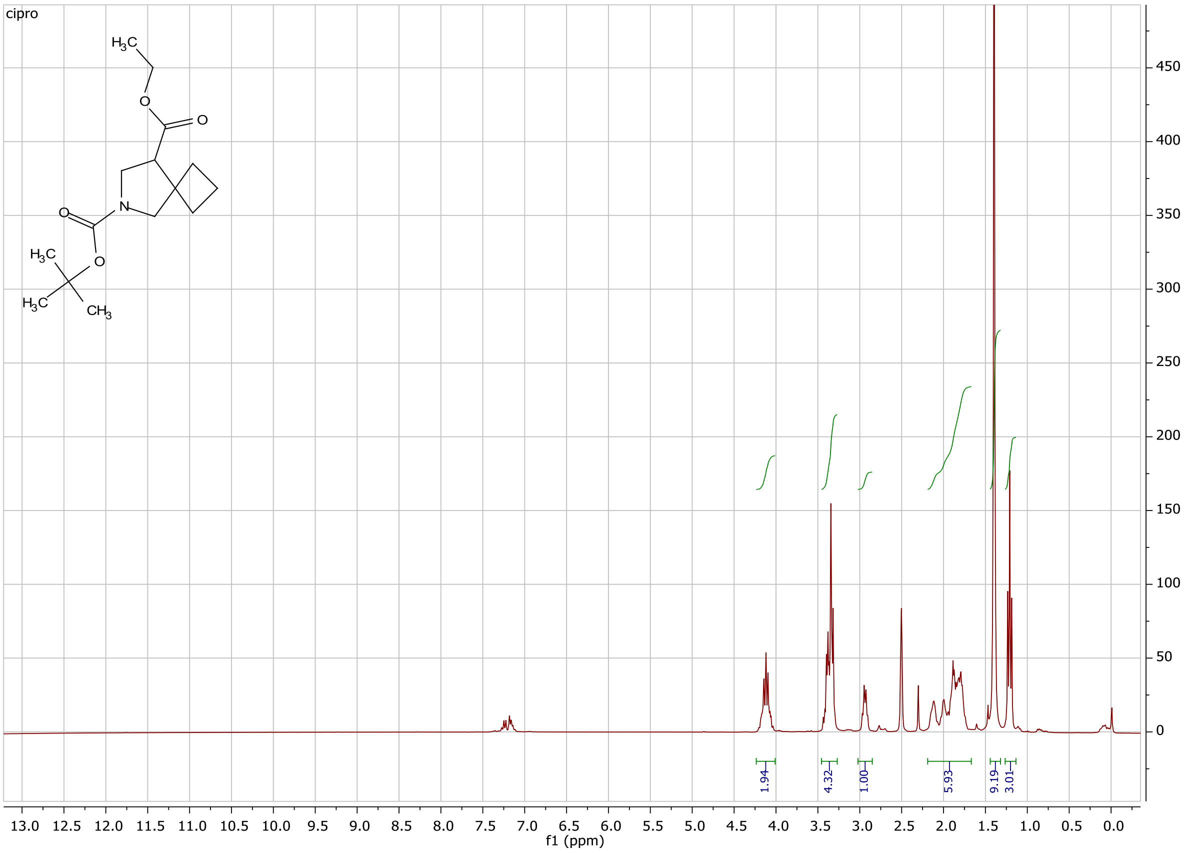

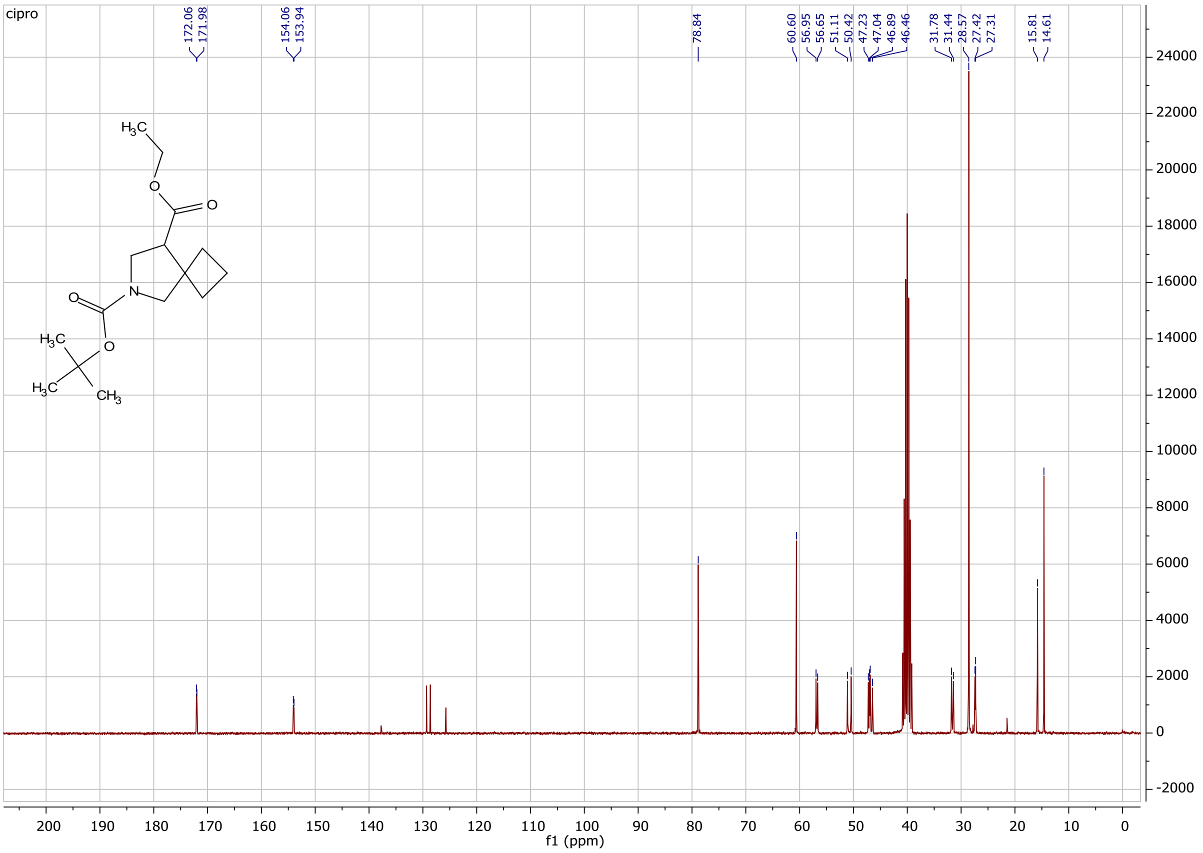

^{1}H and ^{13}C NMR spectra of compound **3b**

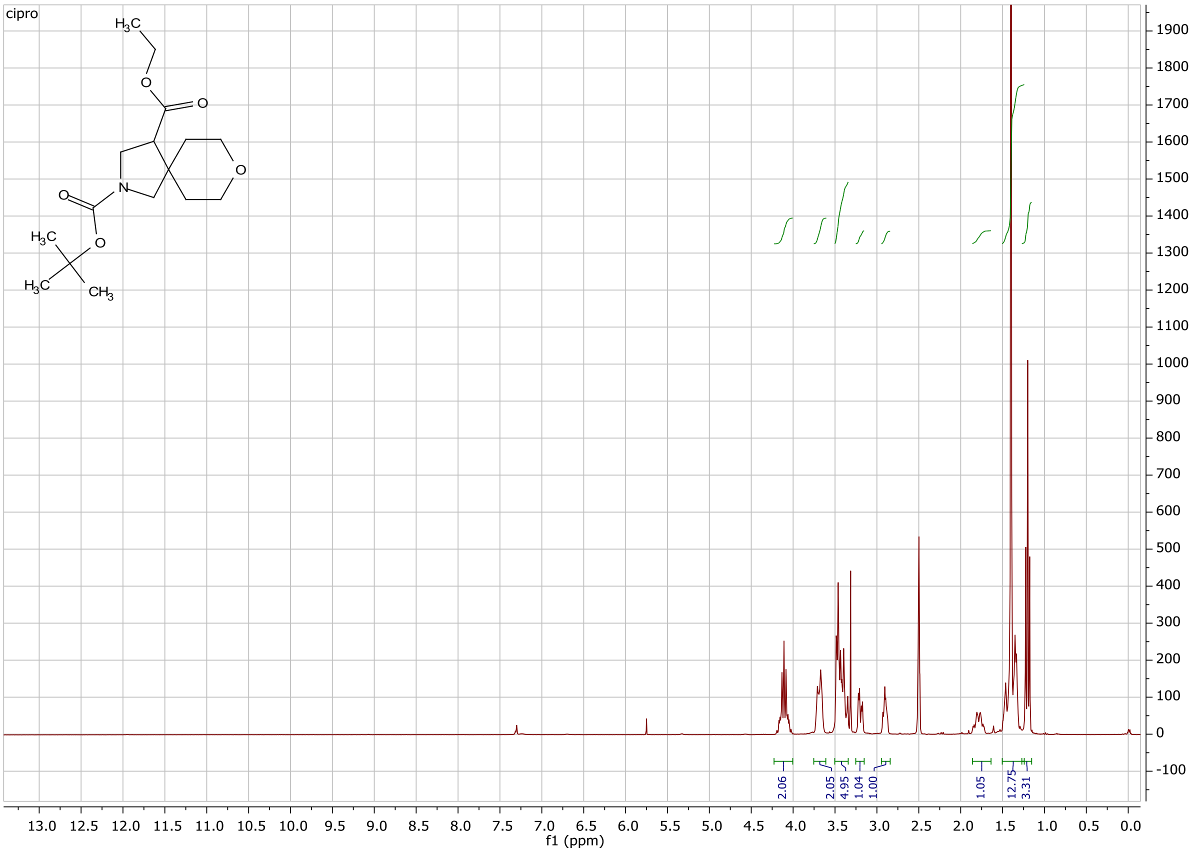

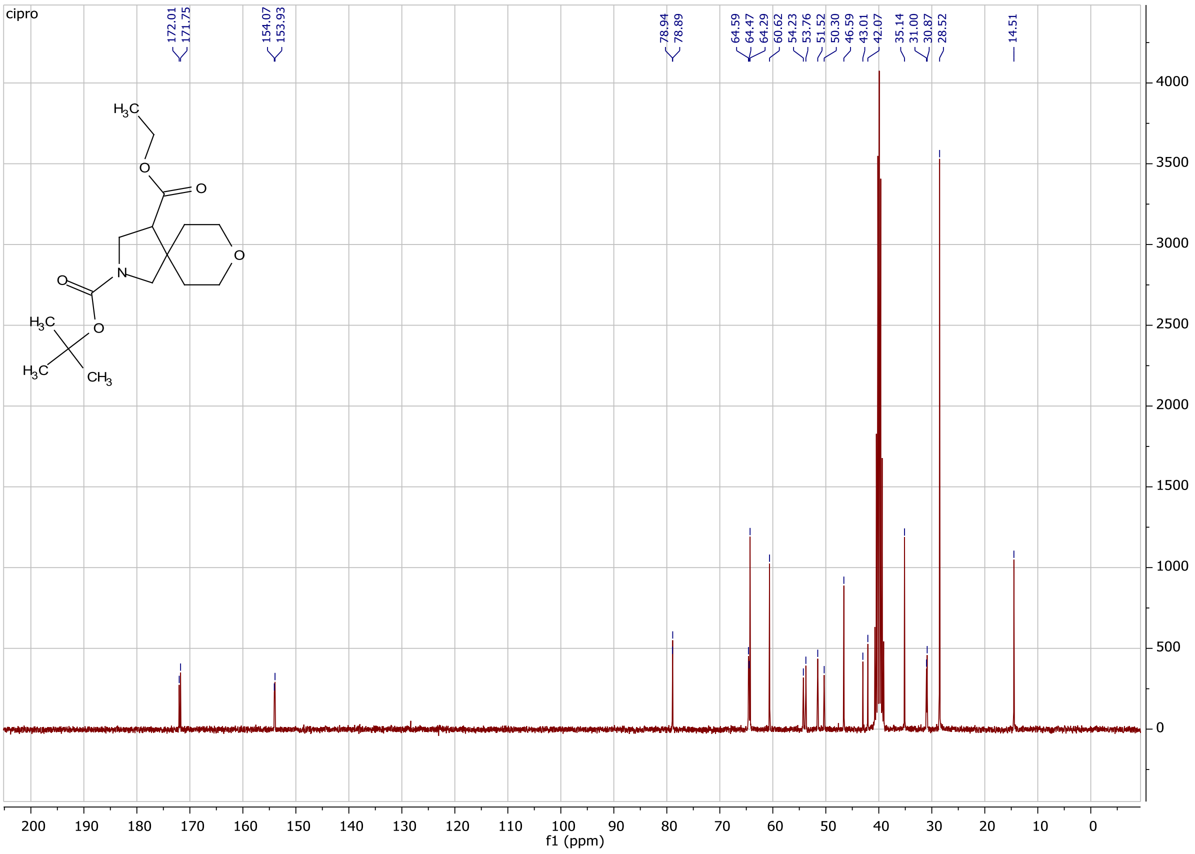

^{1}H and ^{13}C NMR spectra of compound **3c**

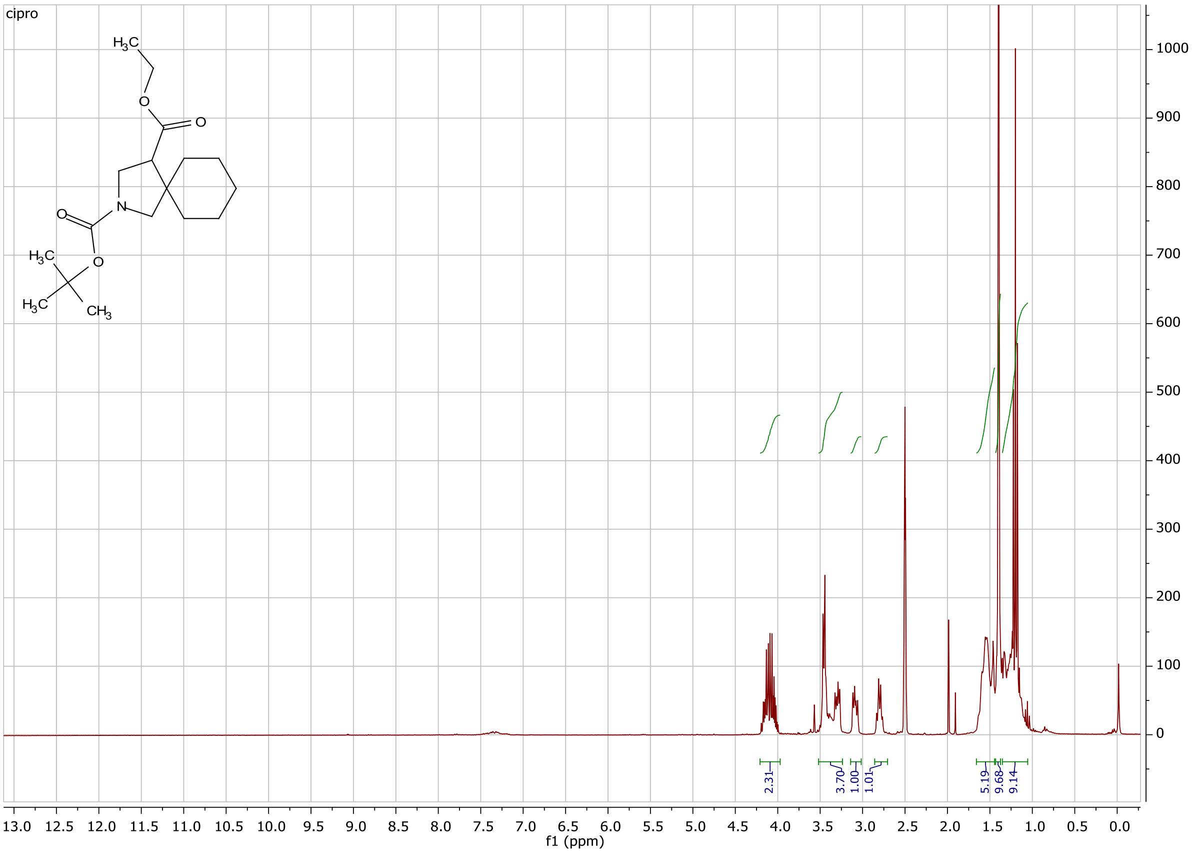

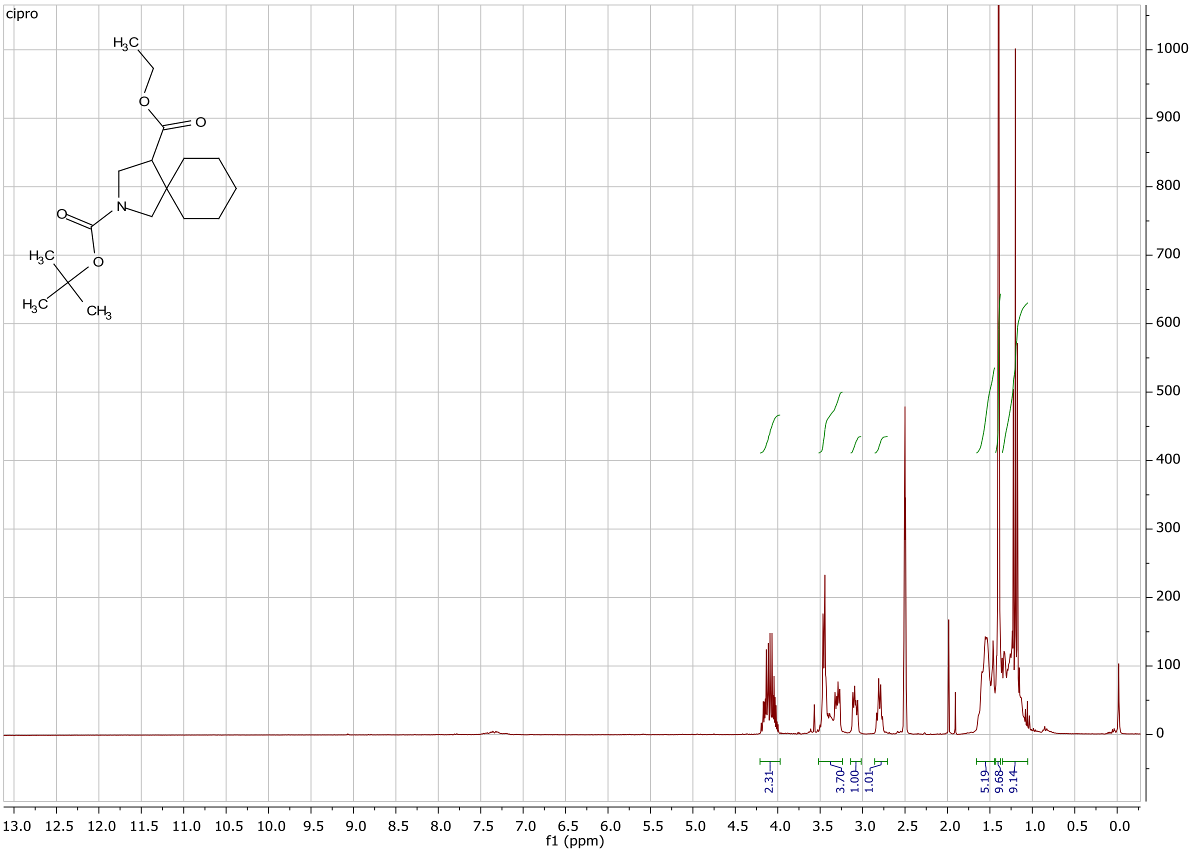

^{1}H and ^{13}C NMR spectra of compound **3d**

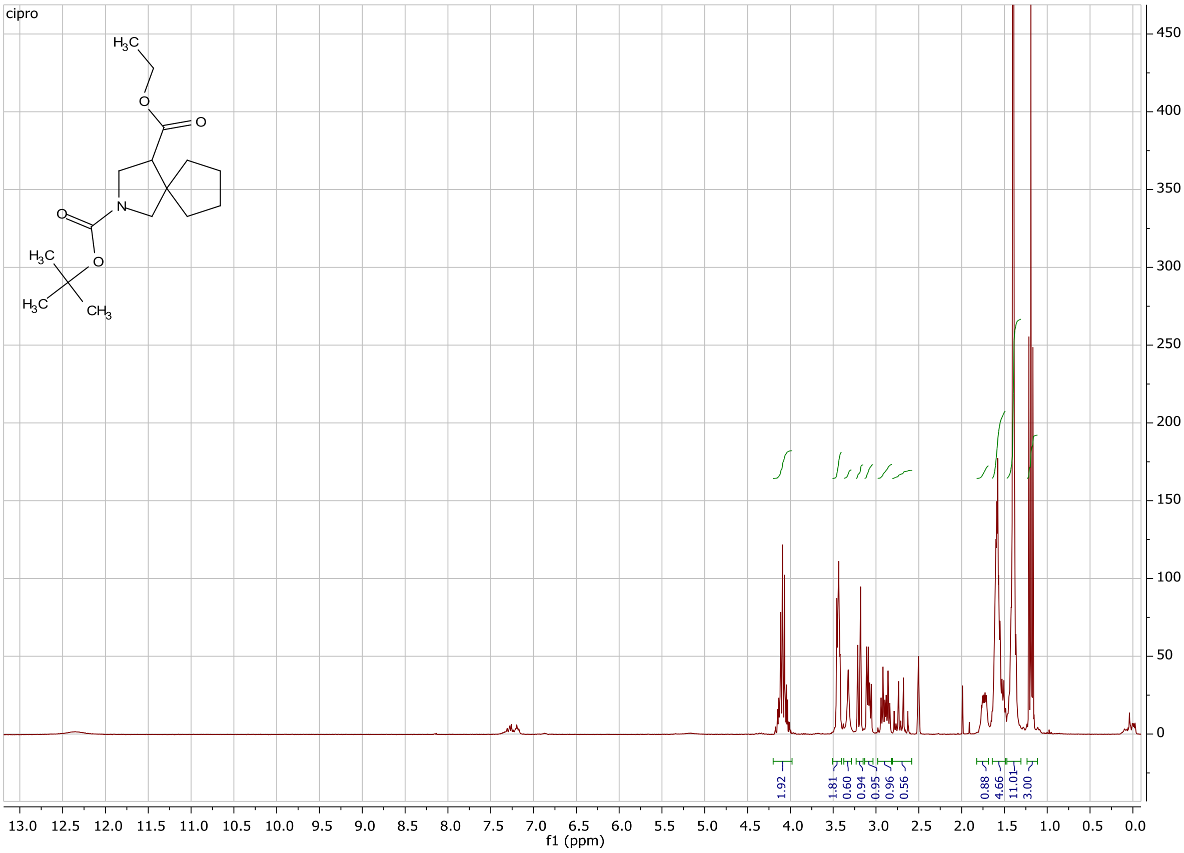

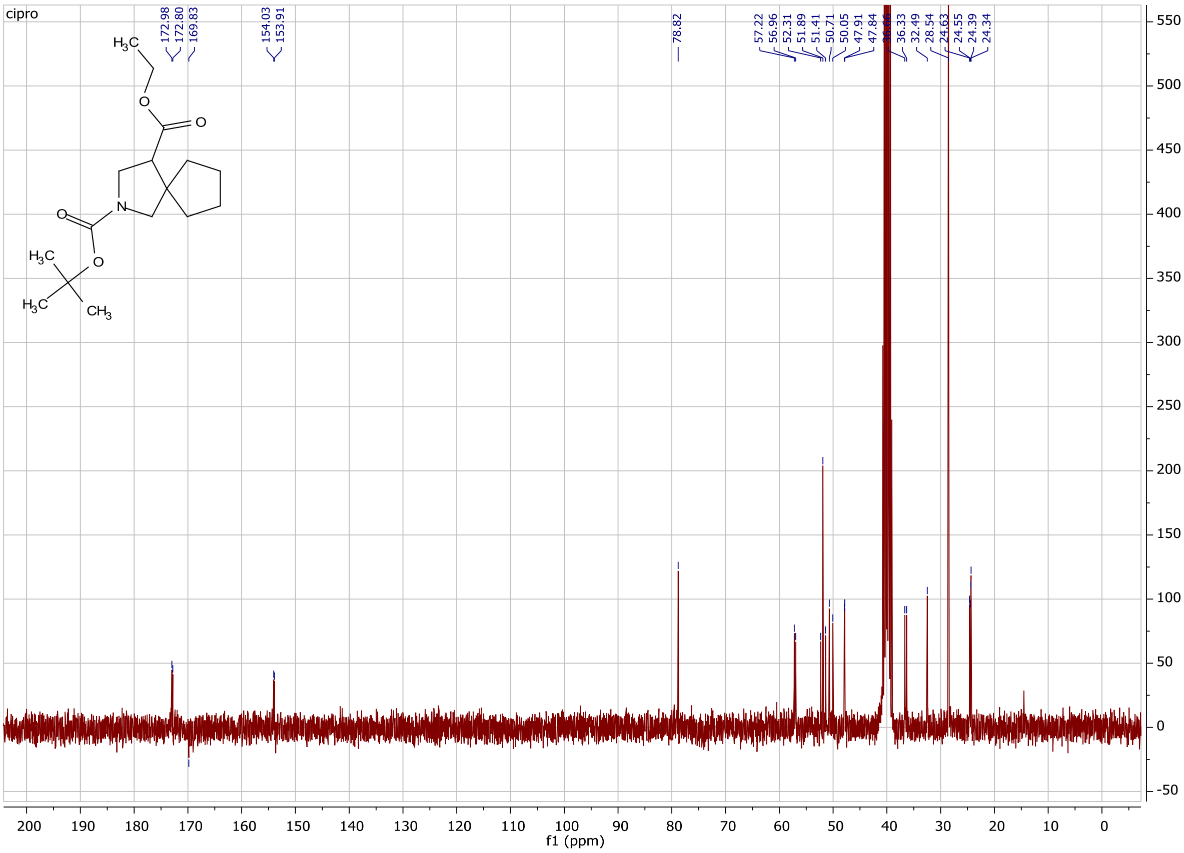

^{1}H and ^{13}C NMR spectra of compound **1a**

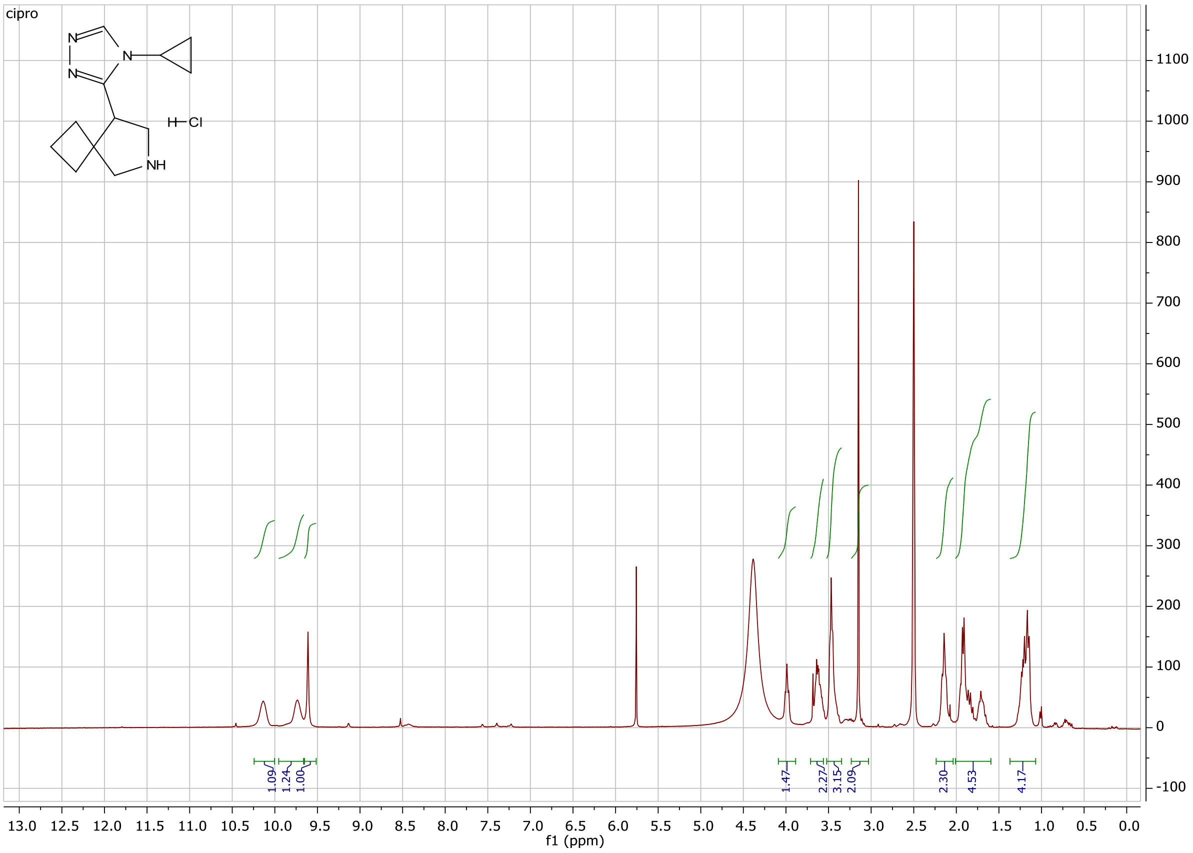

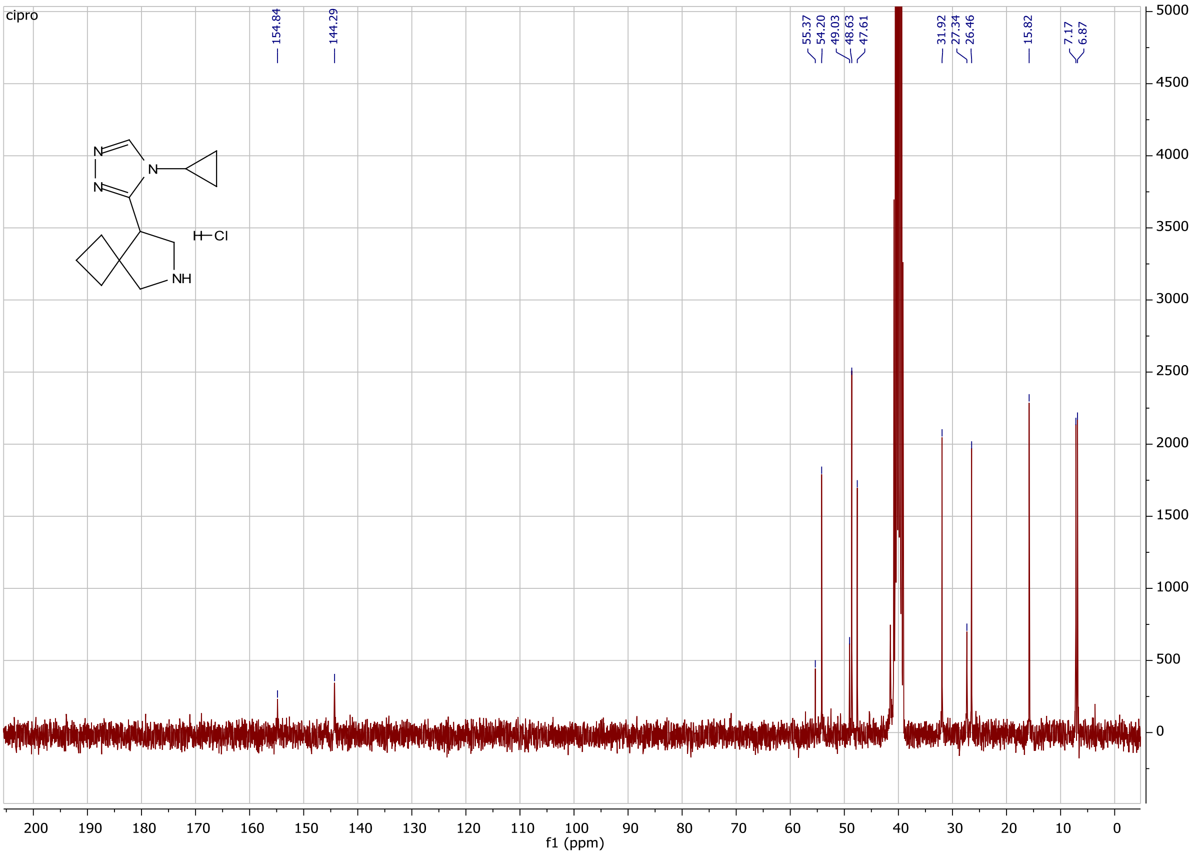

^{1}H and ^{13}C NMR spectra of compound **1b**

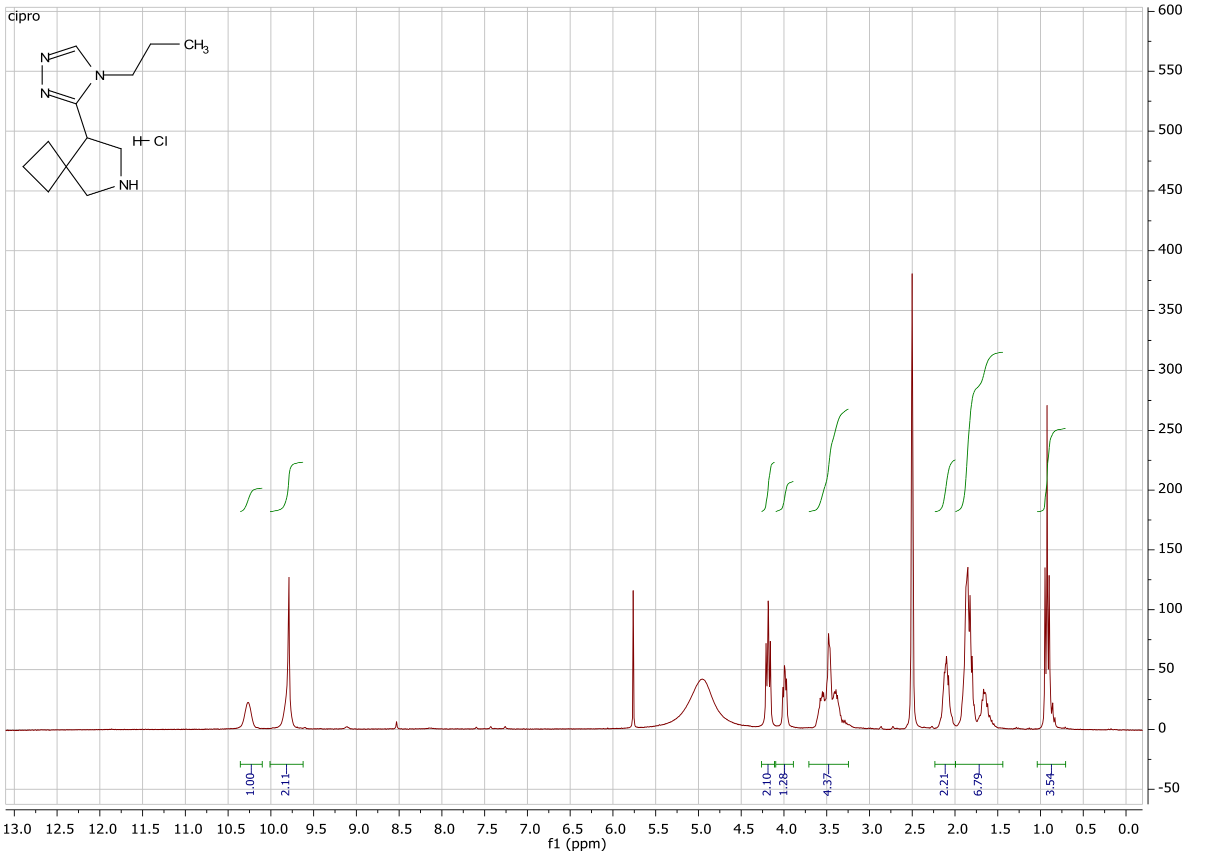

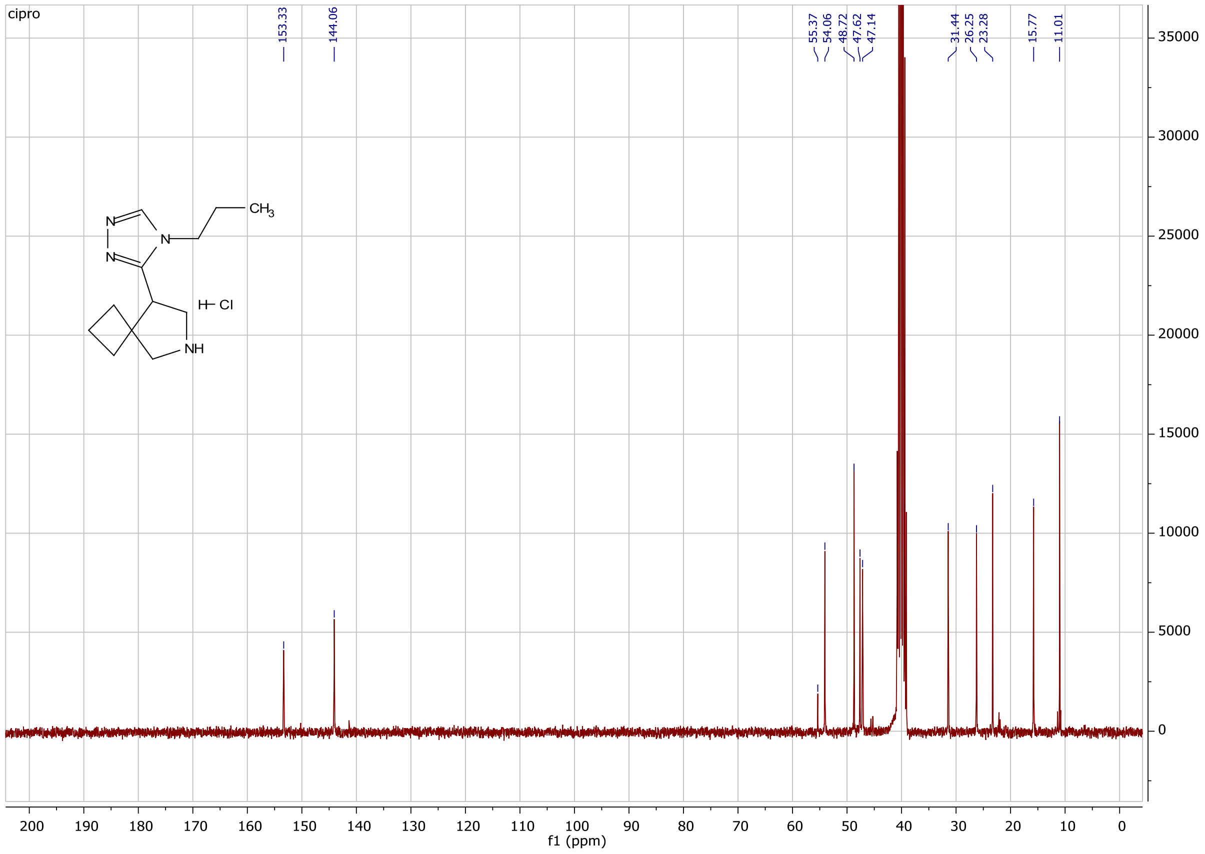

^{1}H and ^{13}C NMR spectra of compound **1c**

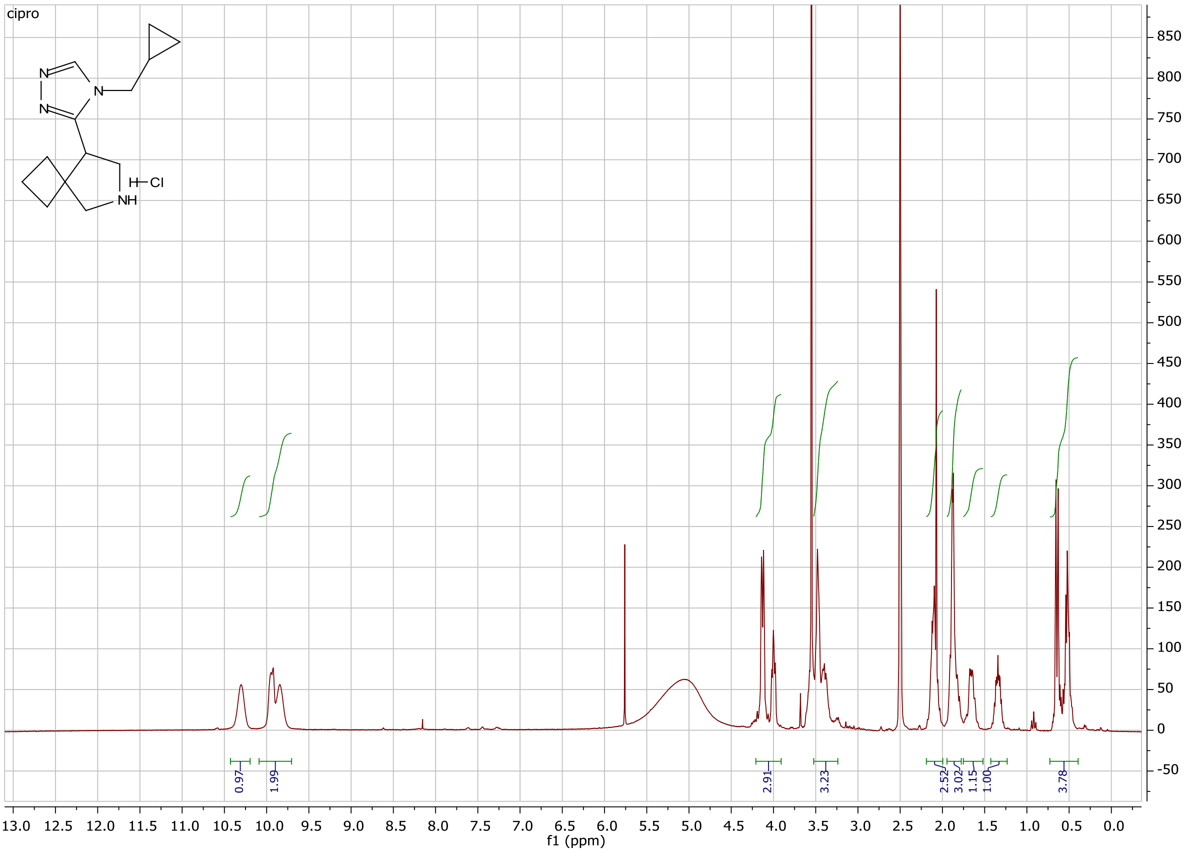

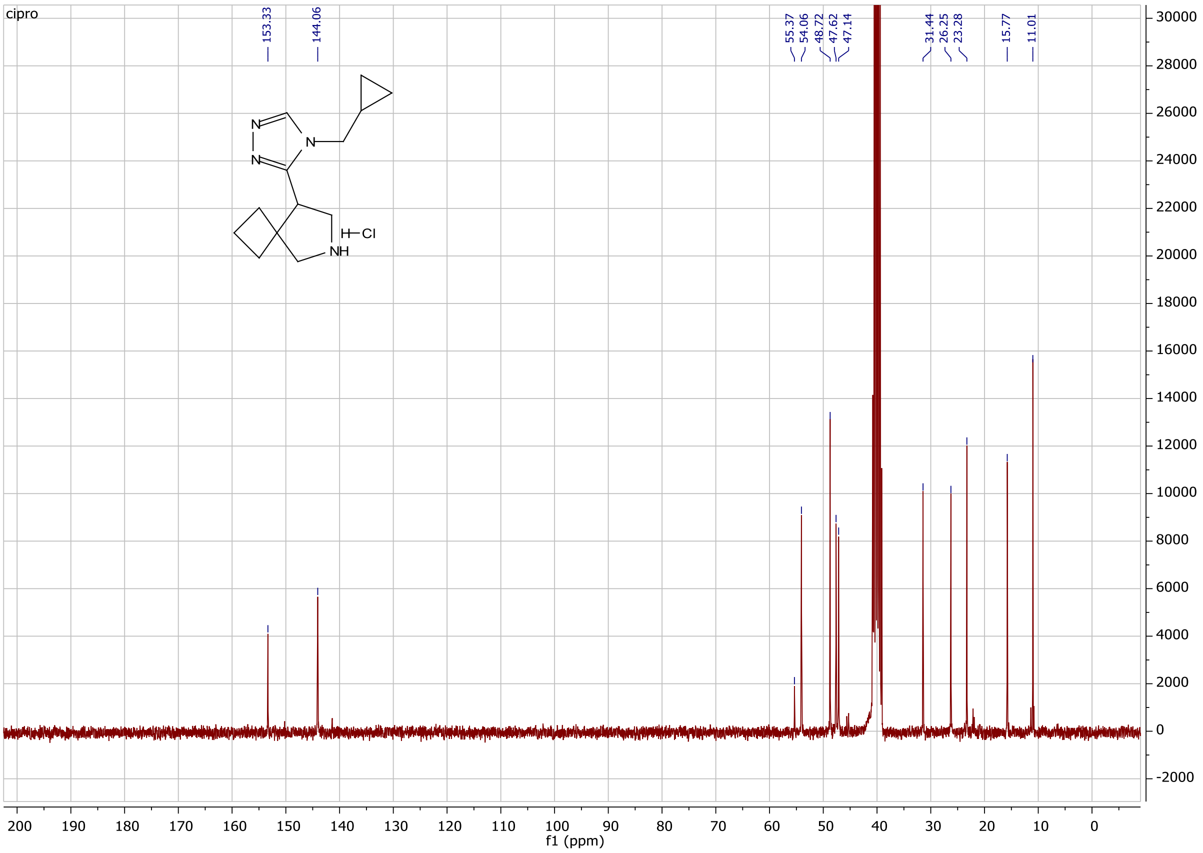

^{1}H and ^{13}C NMR spectra of compound **1d**

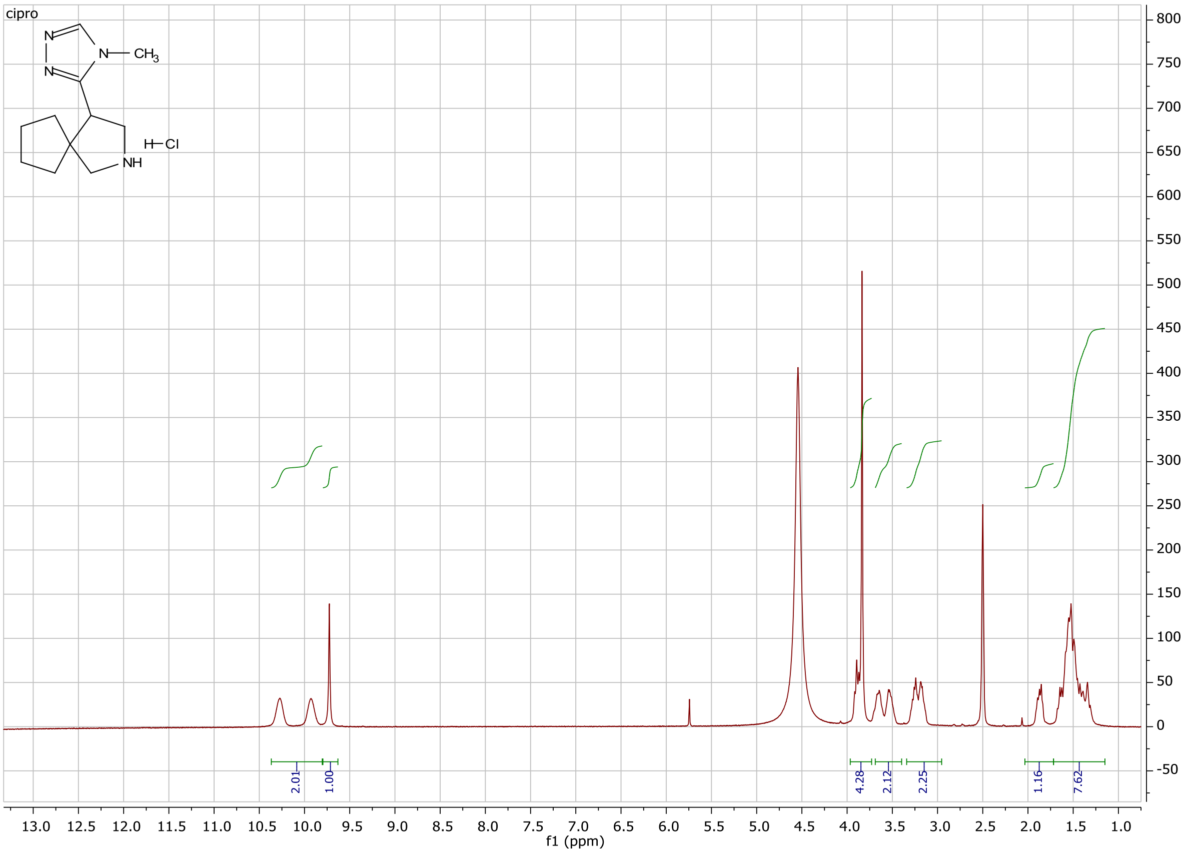

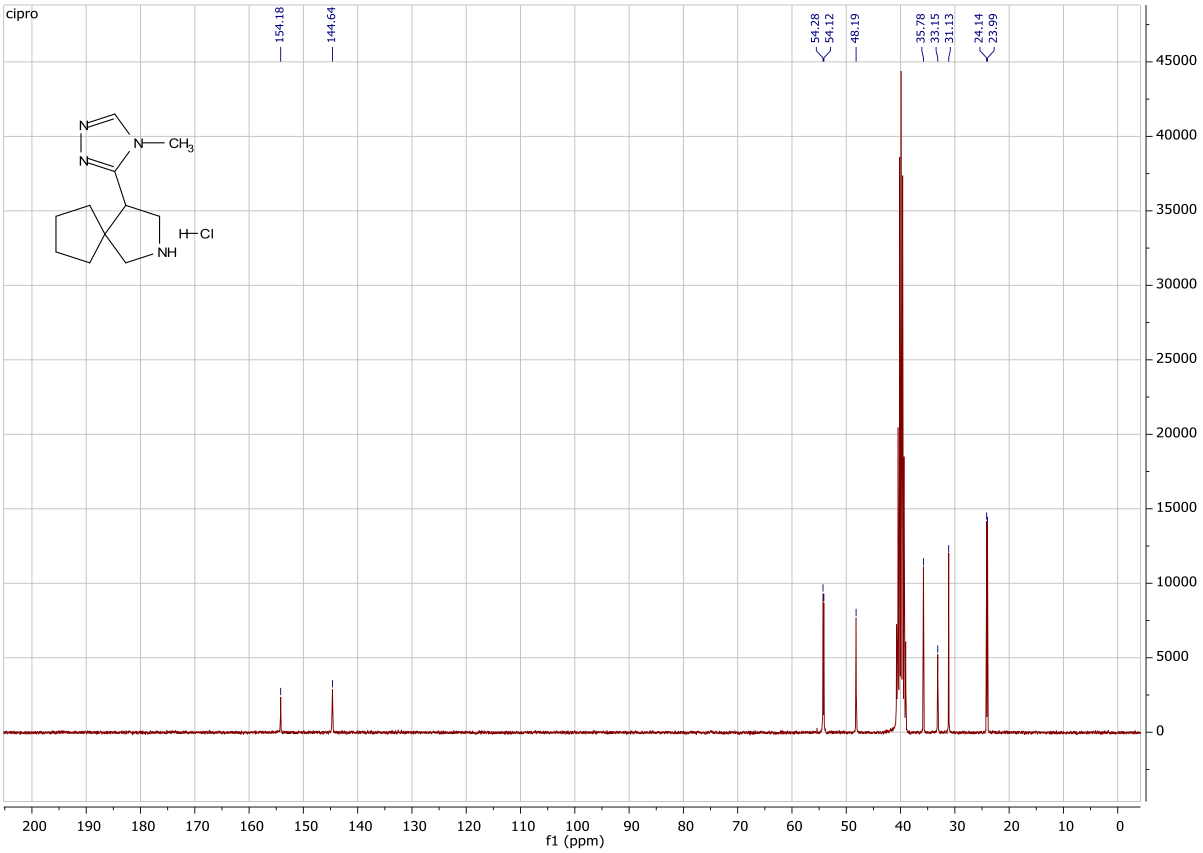

^{1}H and ^{13}C NMR spectra of compound **1e**

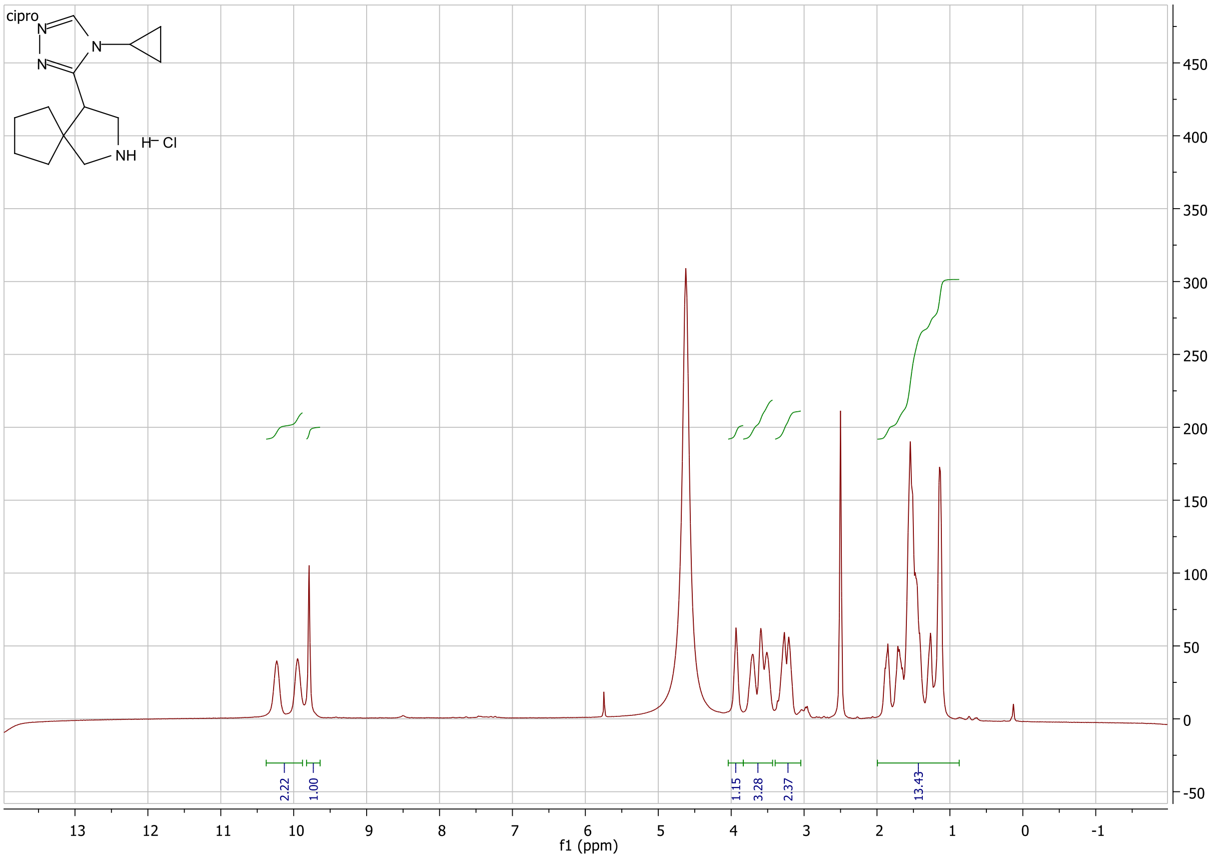

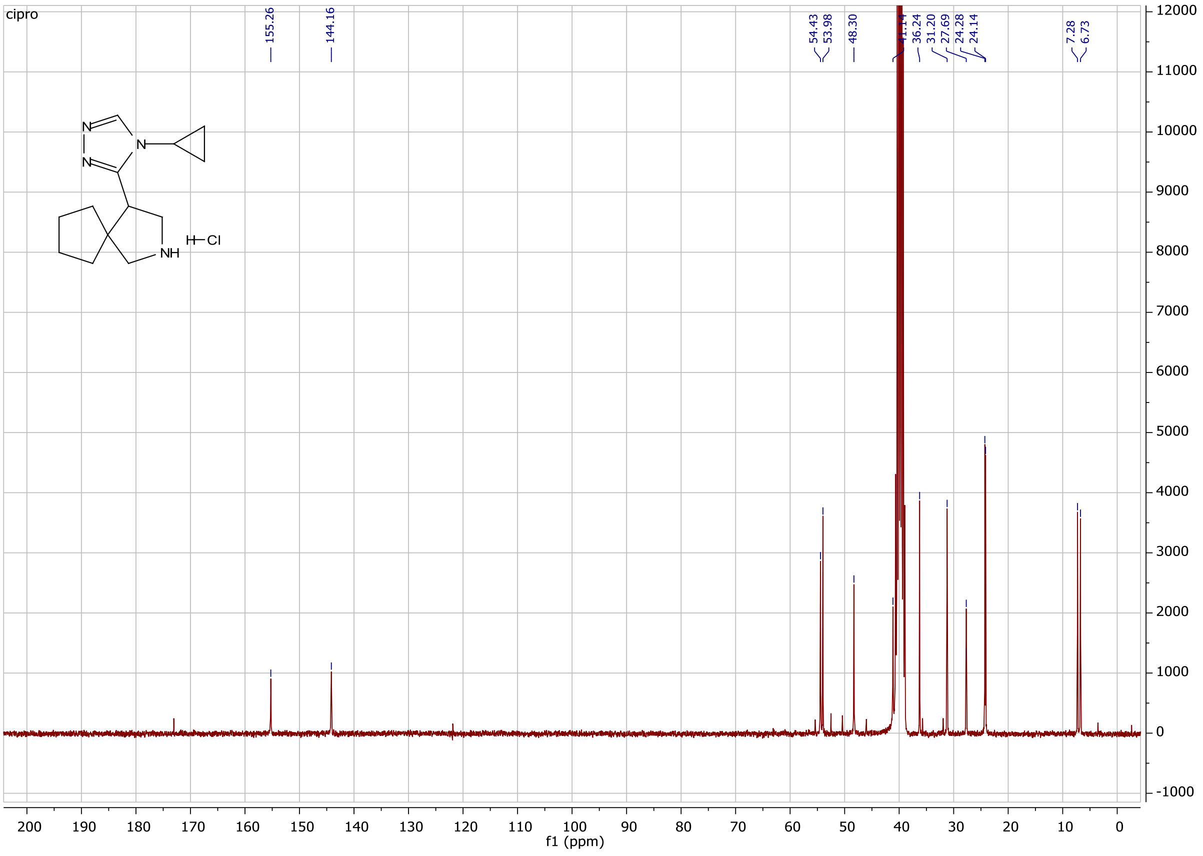

^{1}H and ^{13}C NMR spectra of compound **1f**

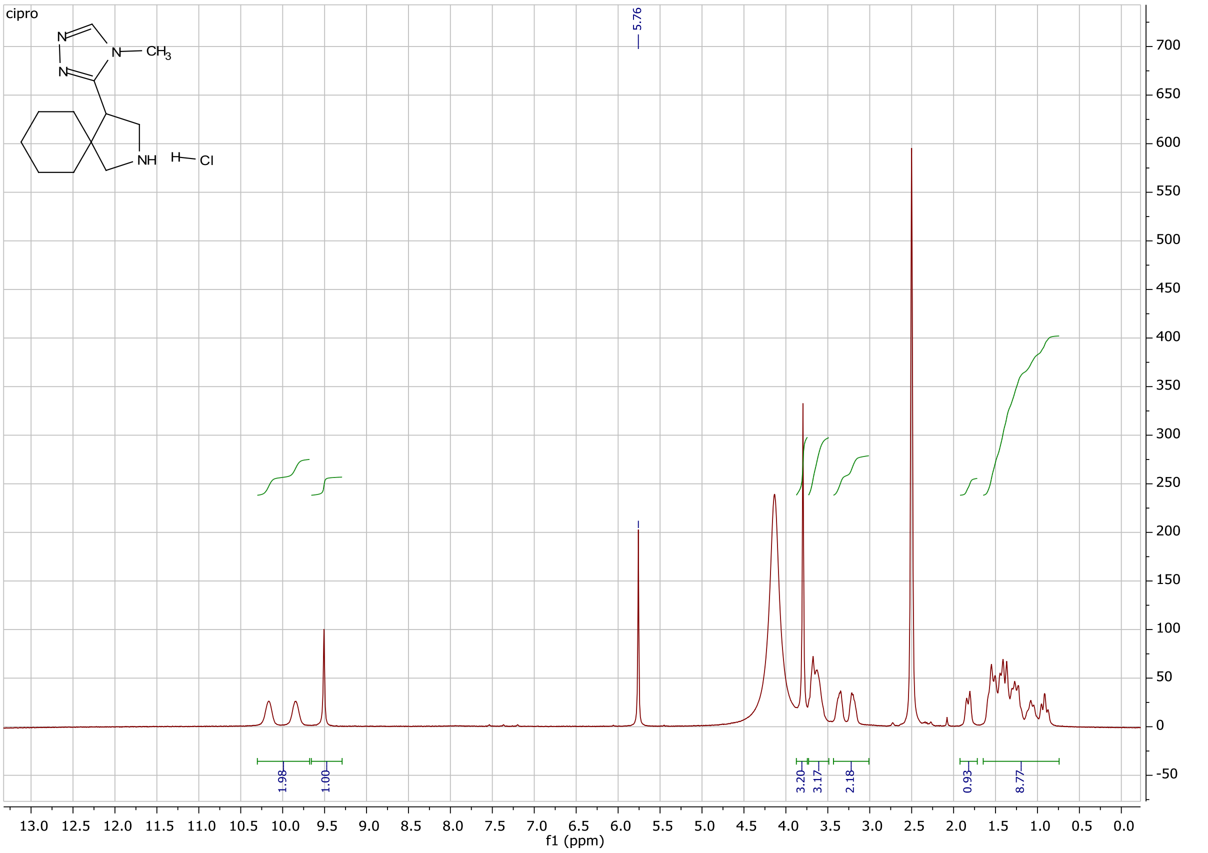

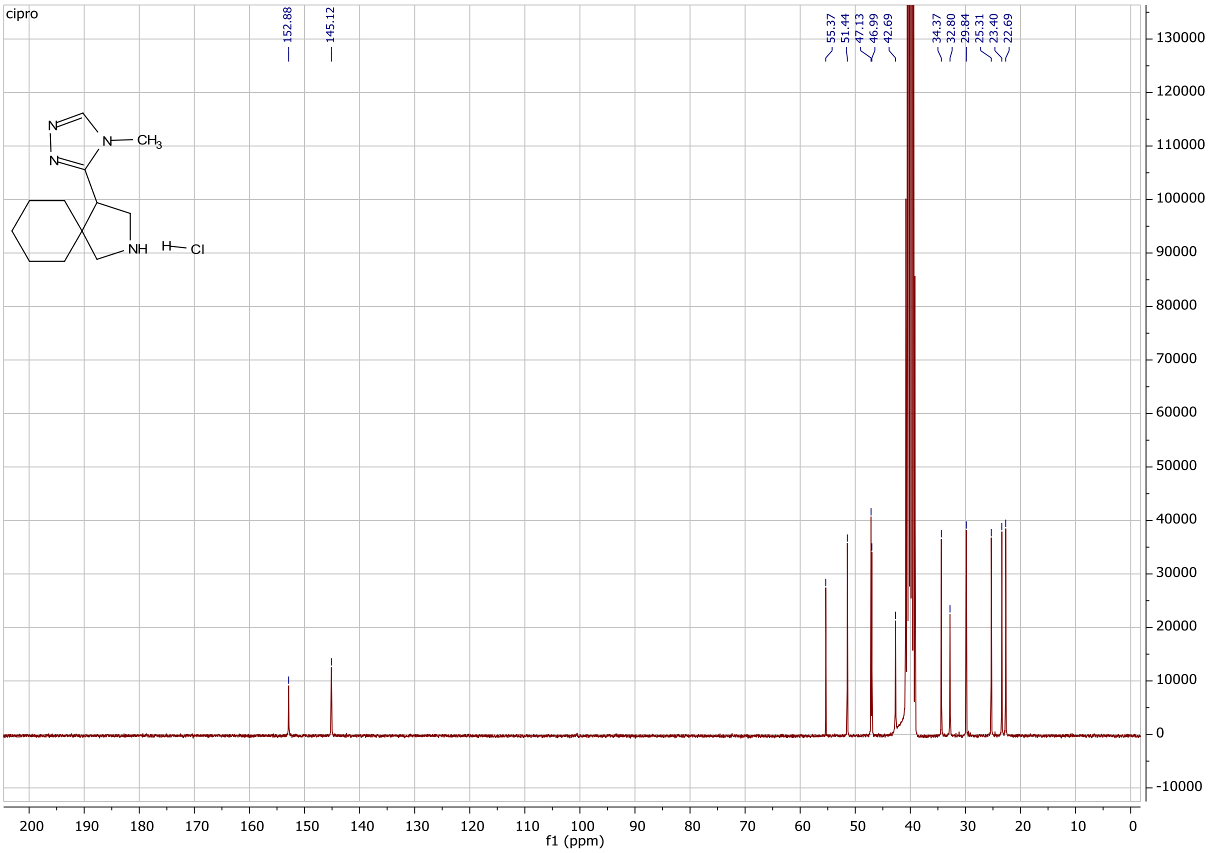

^{1}H and ^{13}C NMR spectra of compound **1g**

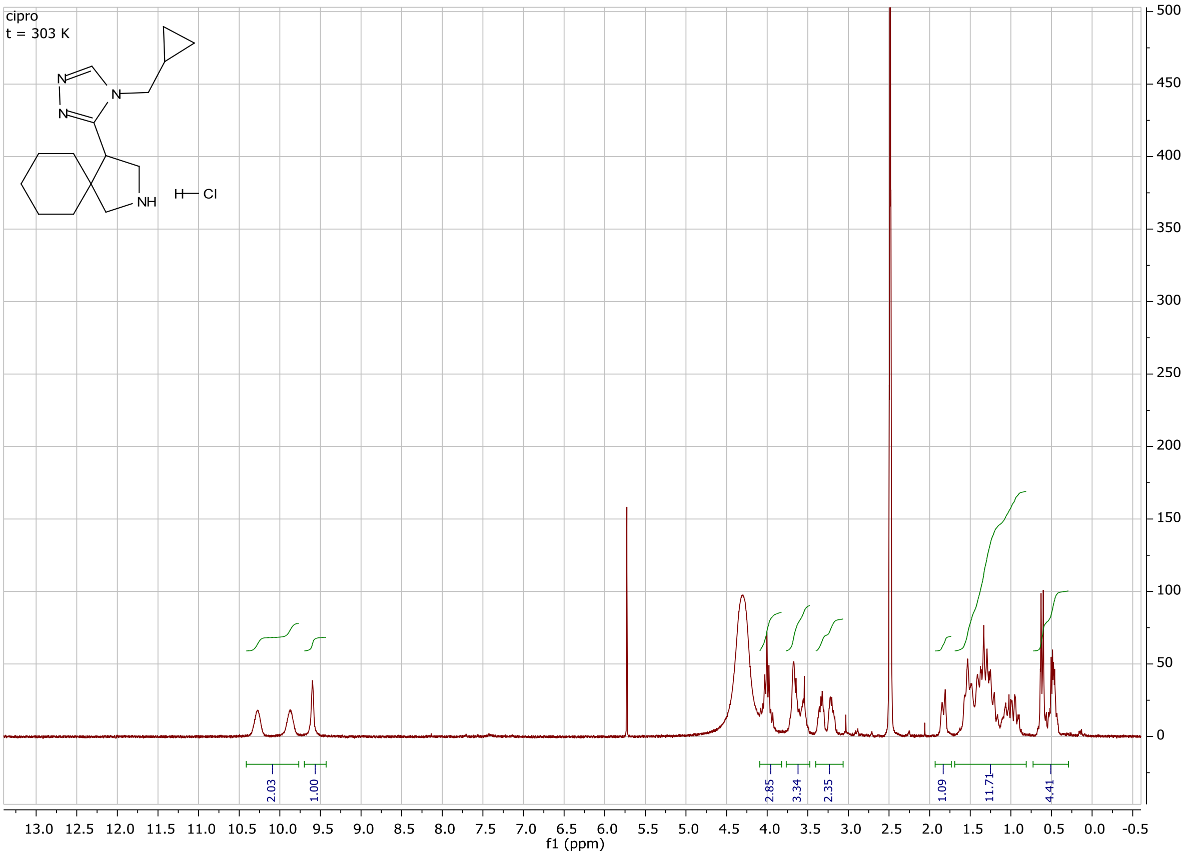

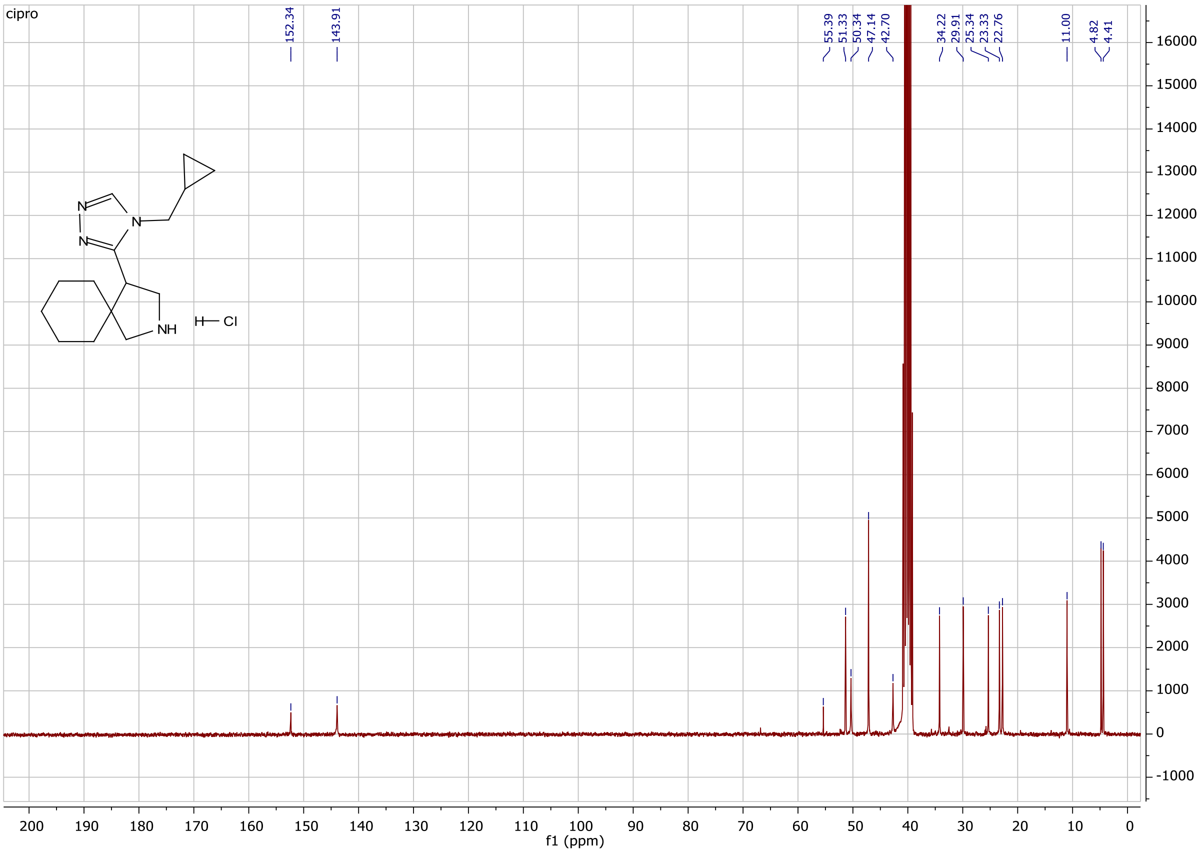

^{1}H and ^{13}C NMR spectra of compound **1h**

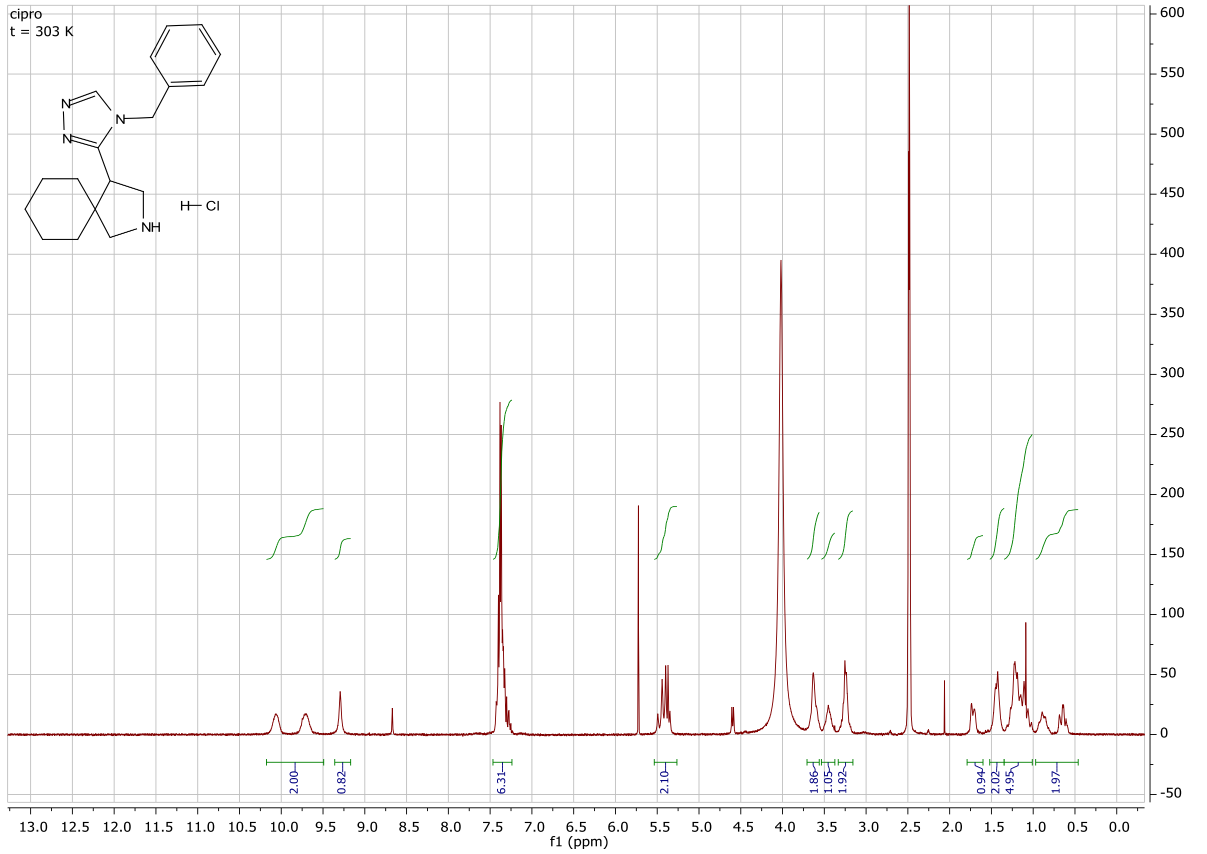

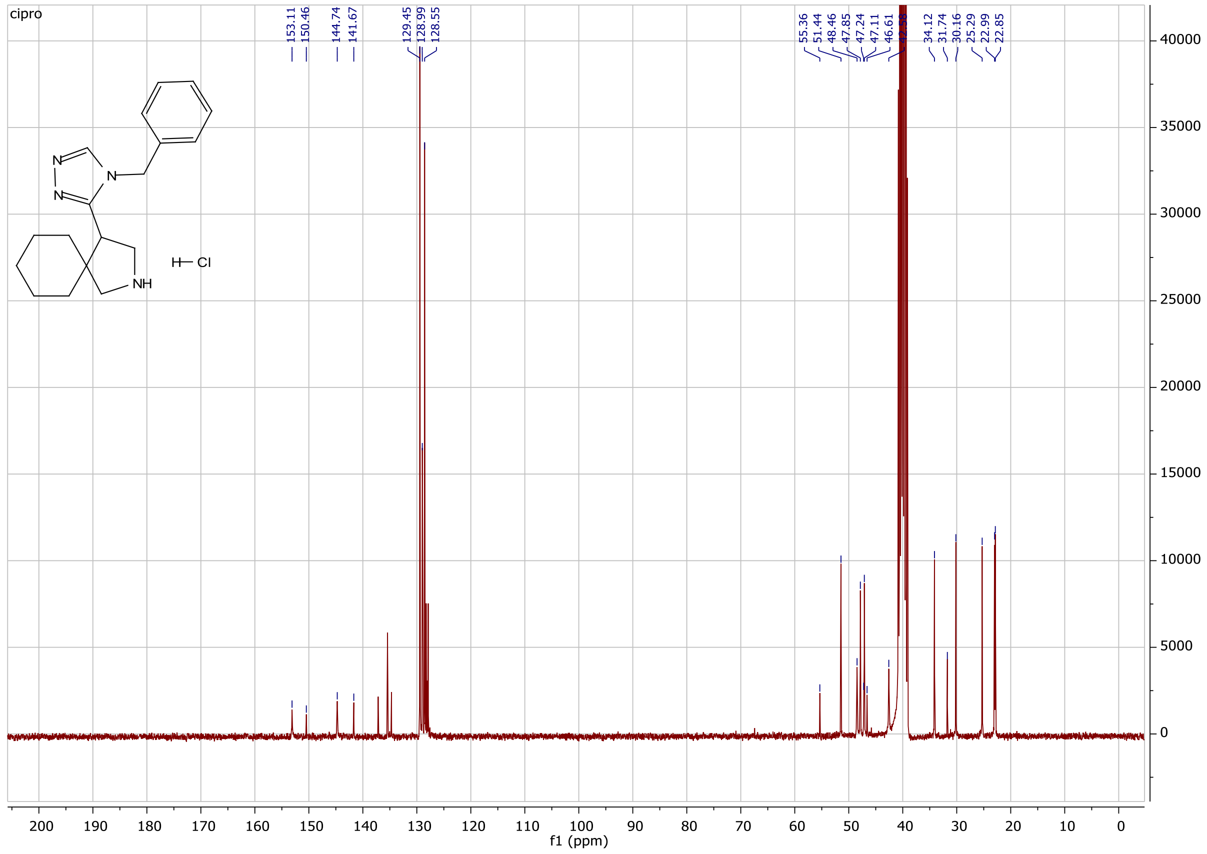

^{1}H and ^{13}C NMR spectra of compound **1i**

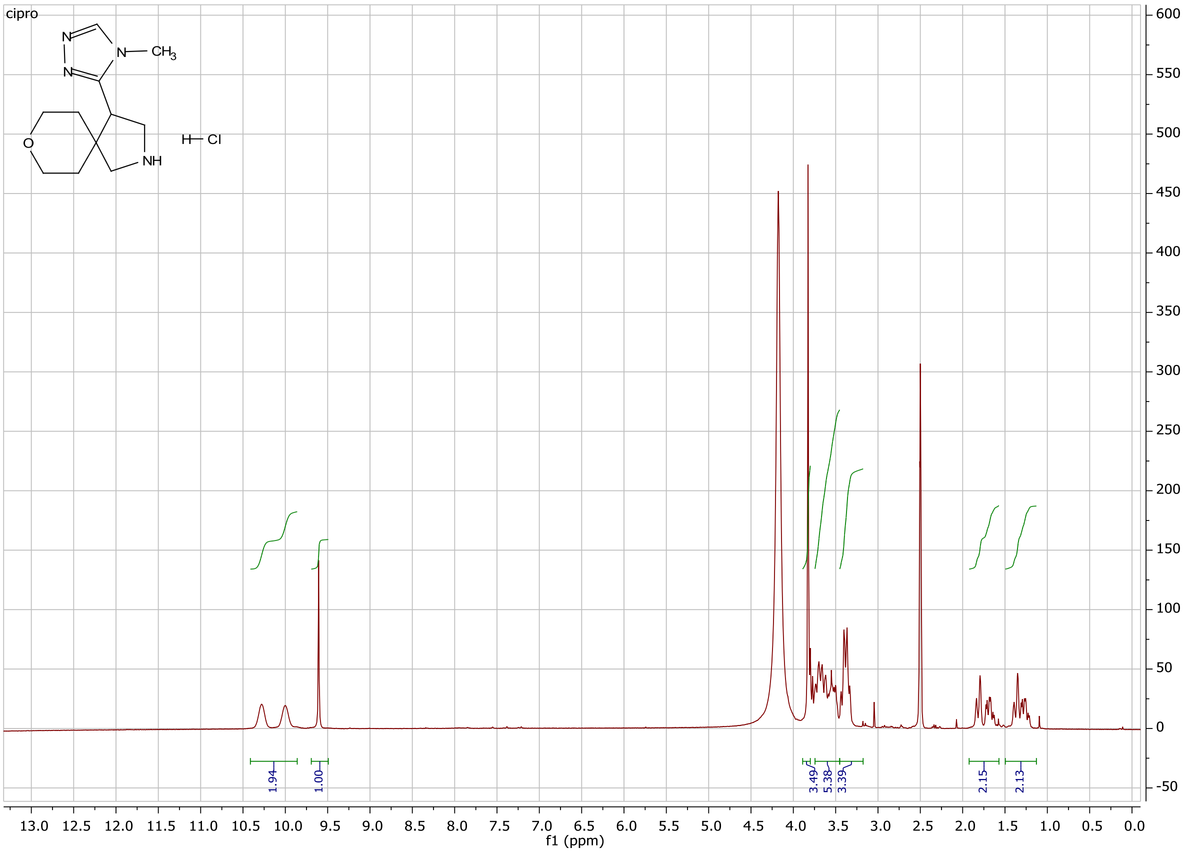

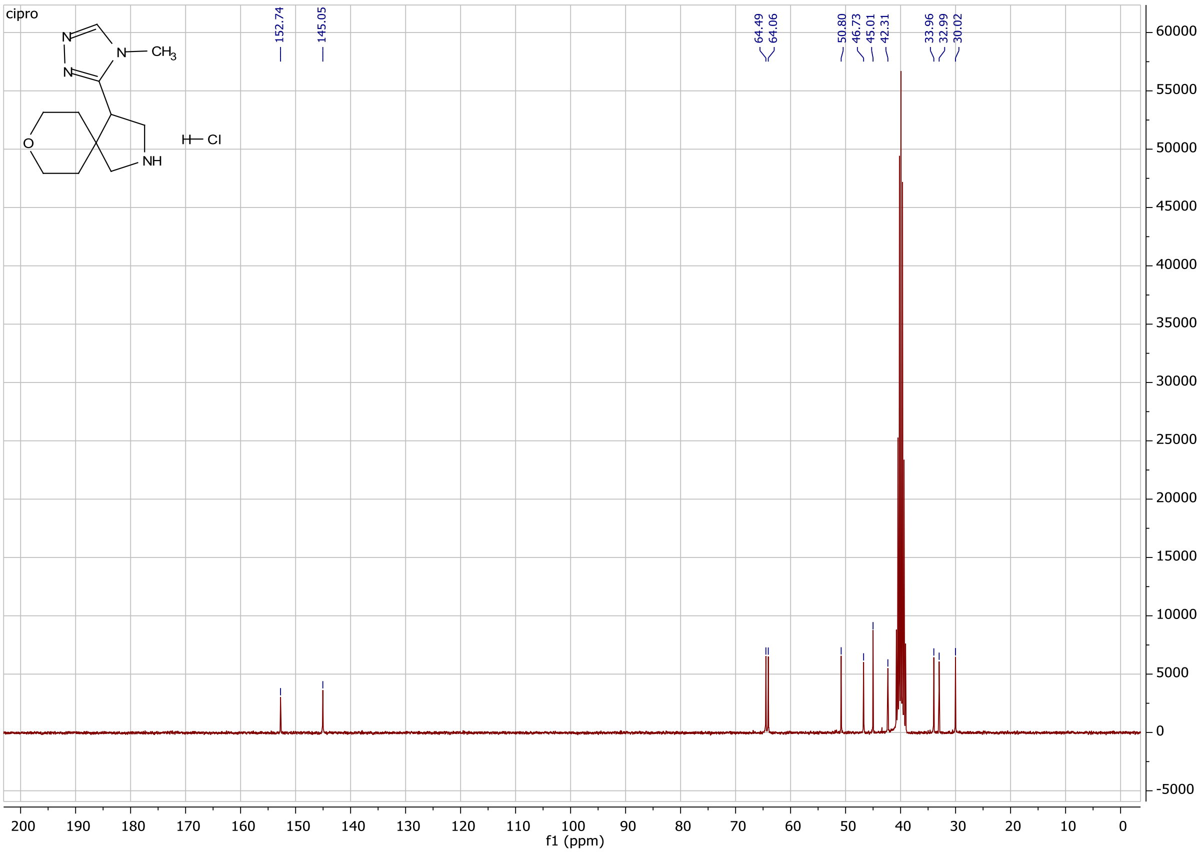

^{1}H and ^{13}C NMR spectra of compound **1j**

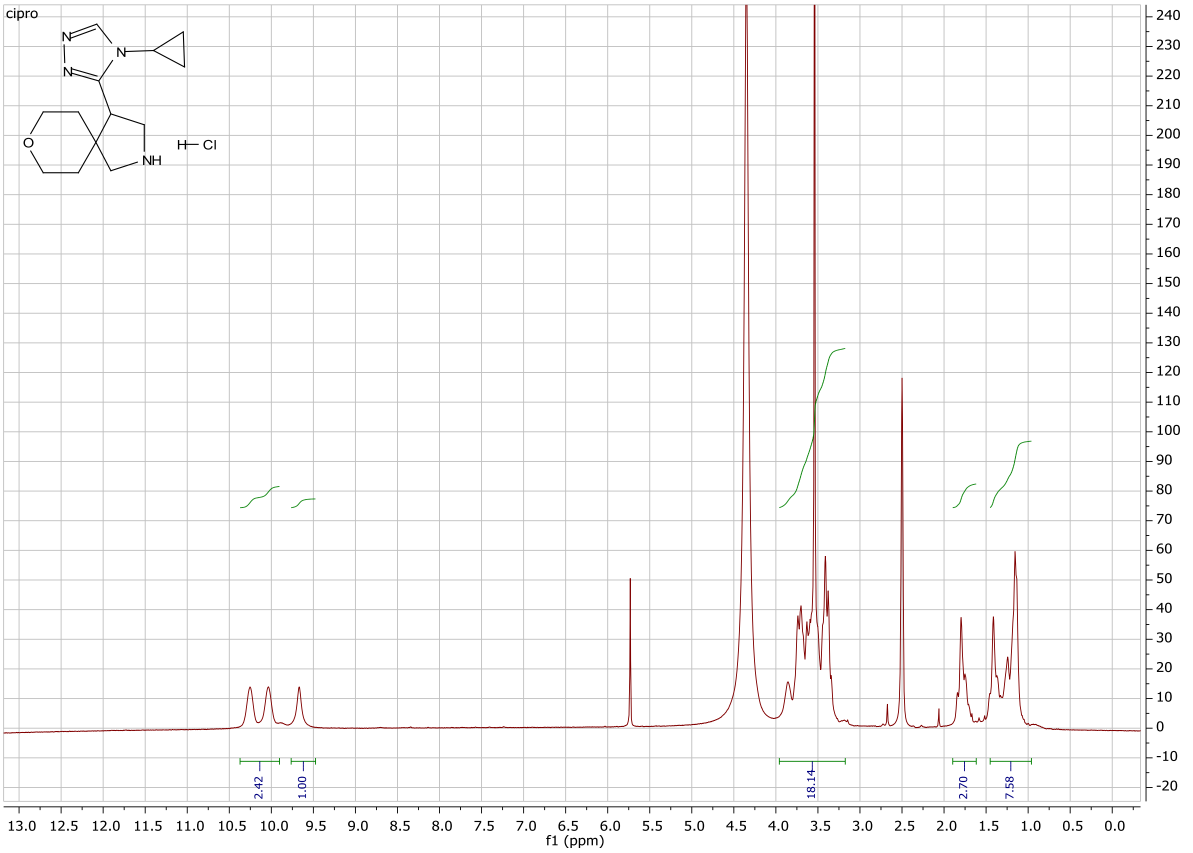

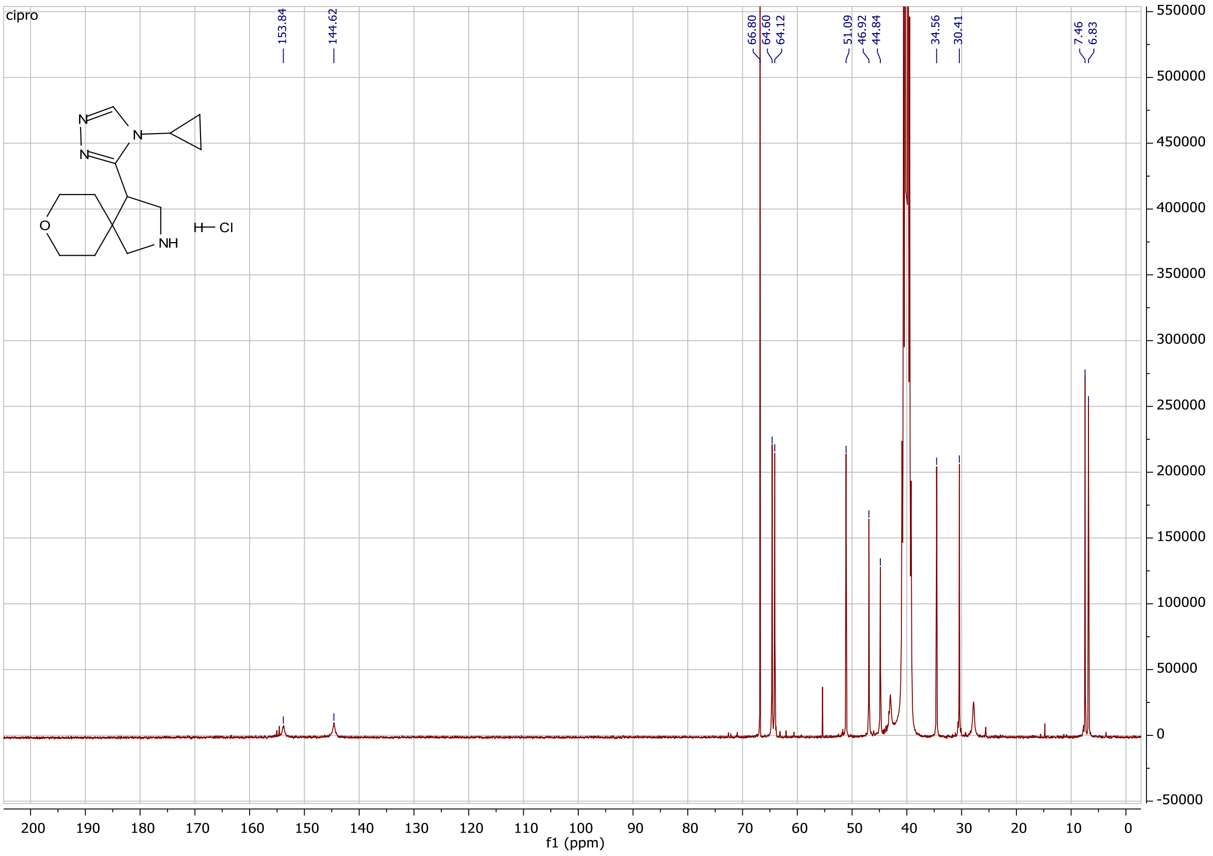

^{1}H and ^{13}C NMR spectra of compound **1k**

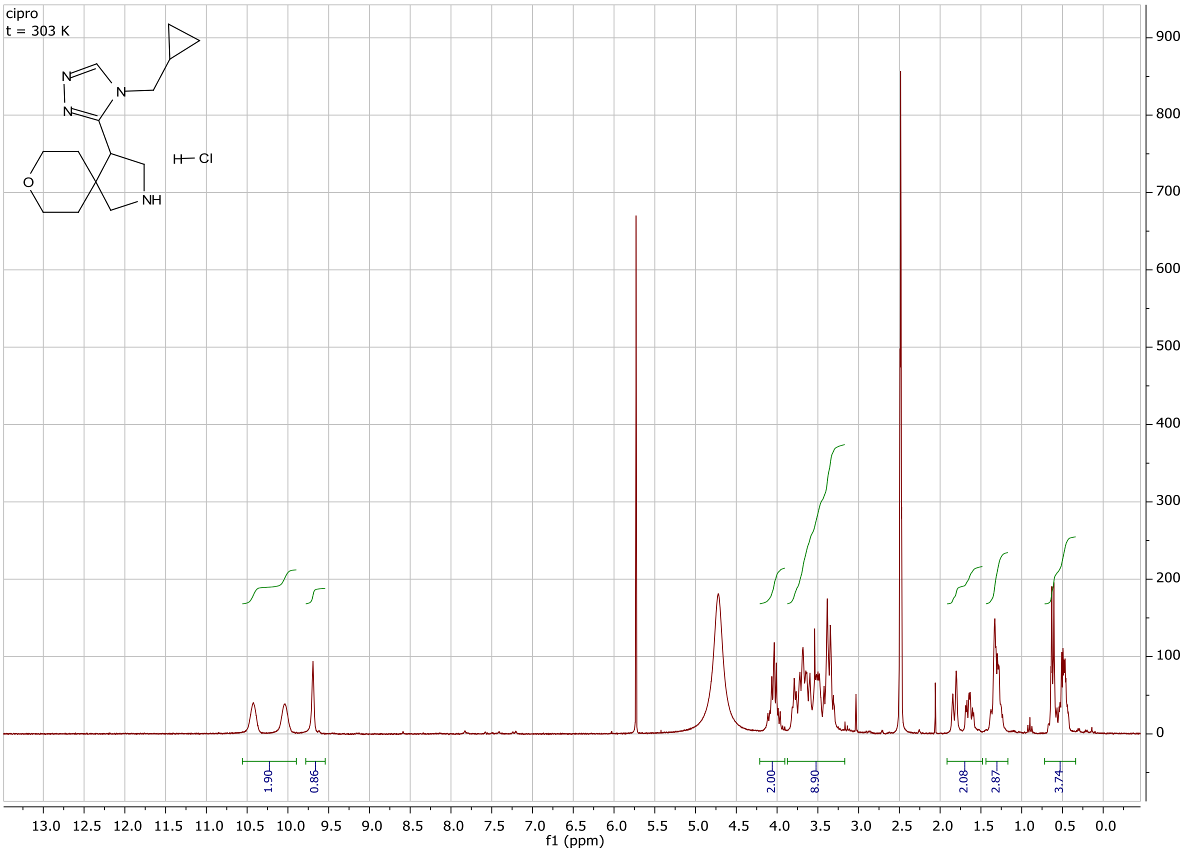

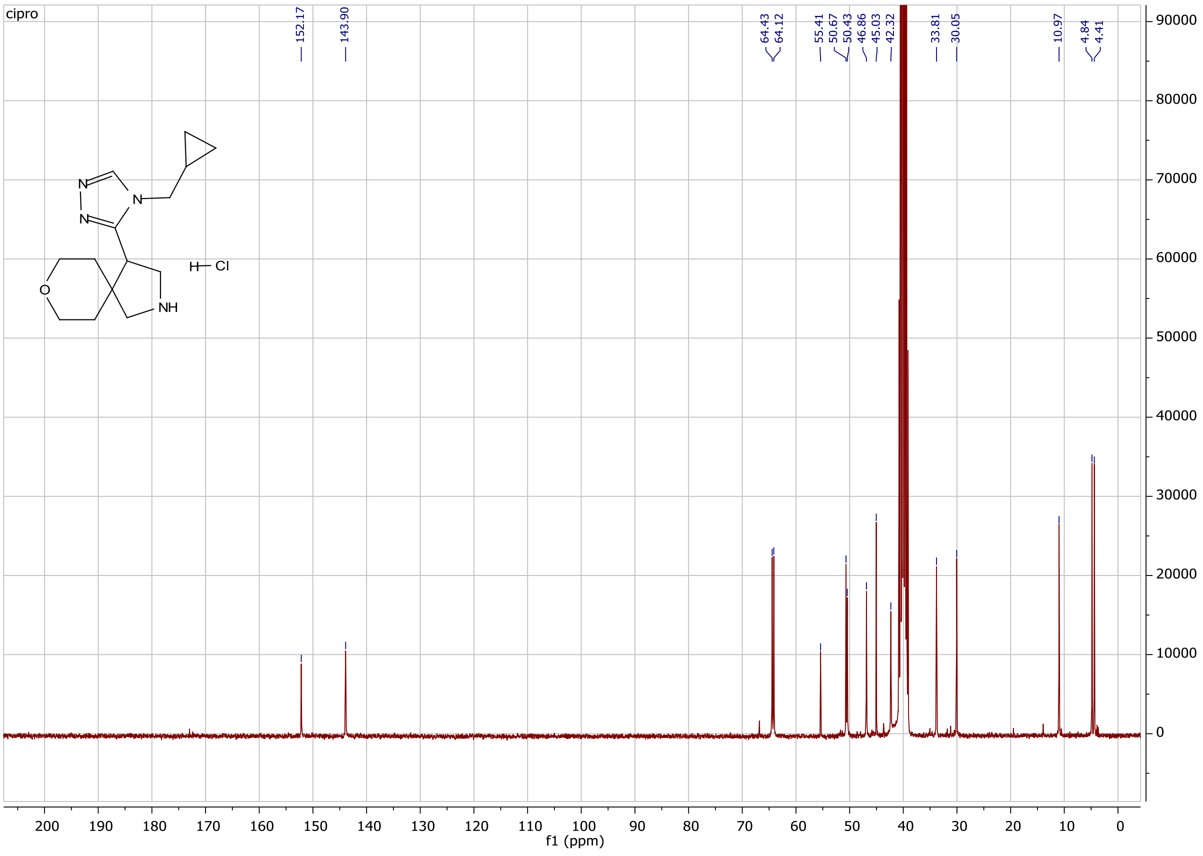

^{1}H and ^{13}C NMR spectra of compound **6a**

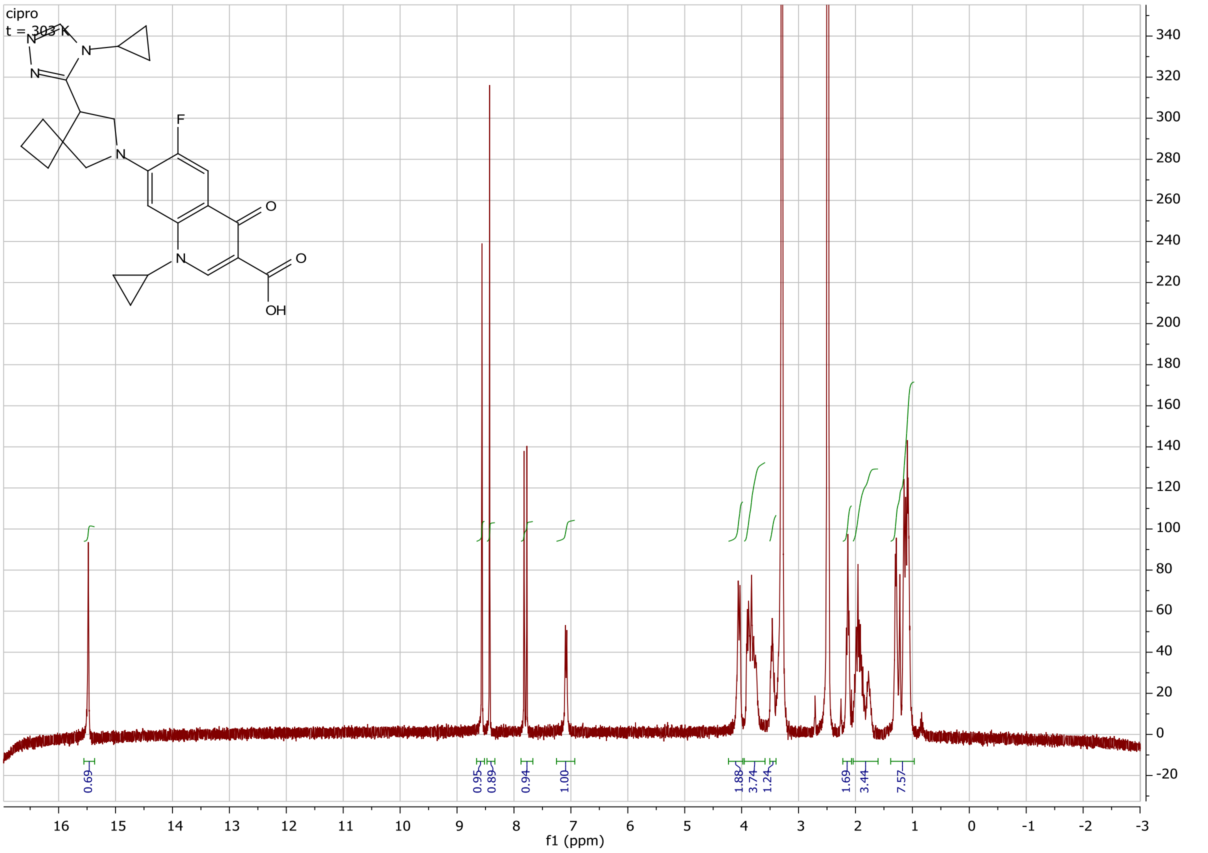

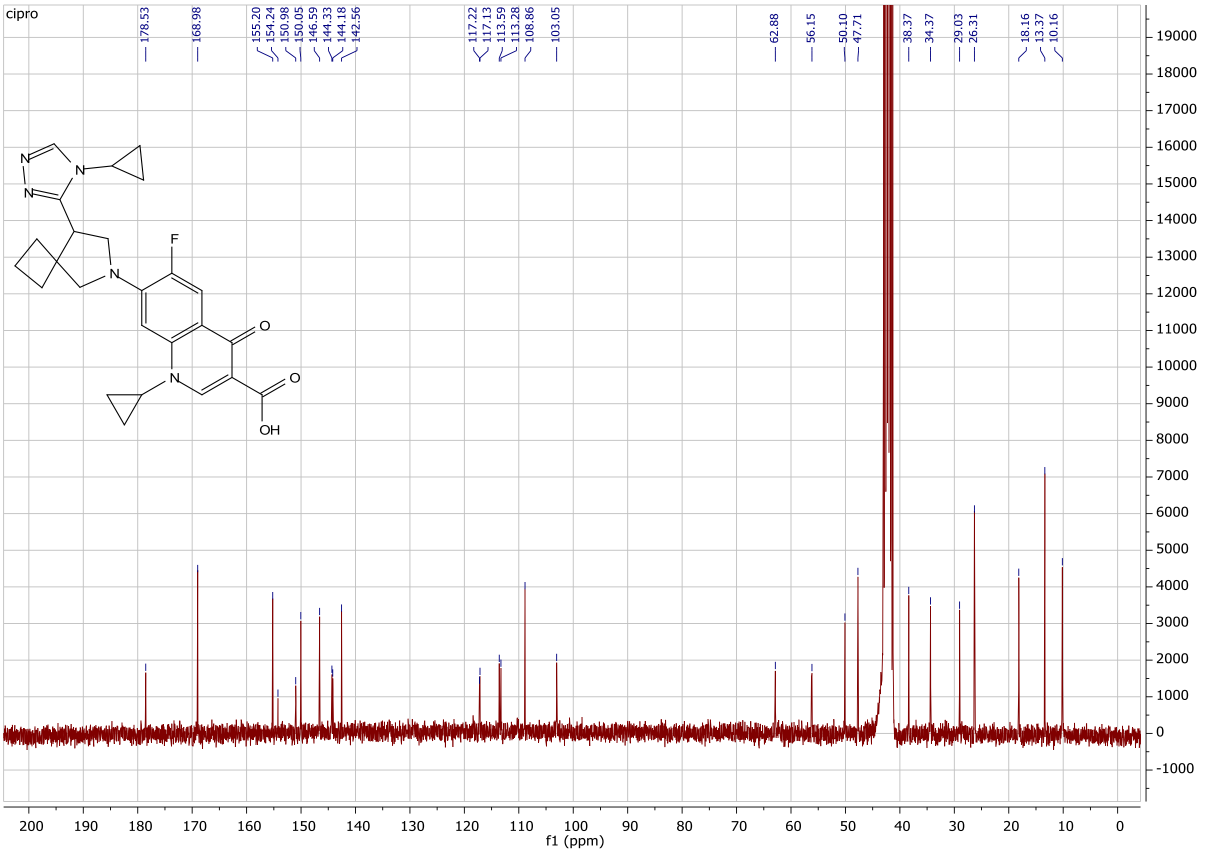

^{1}H and ^{13}C NMR spectra of compound **6b**

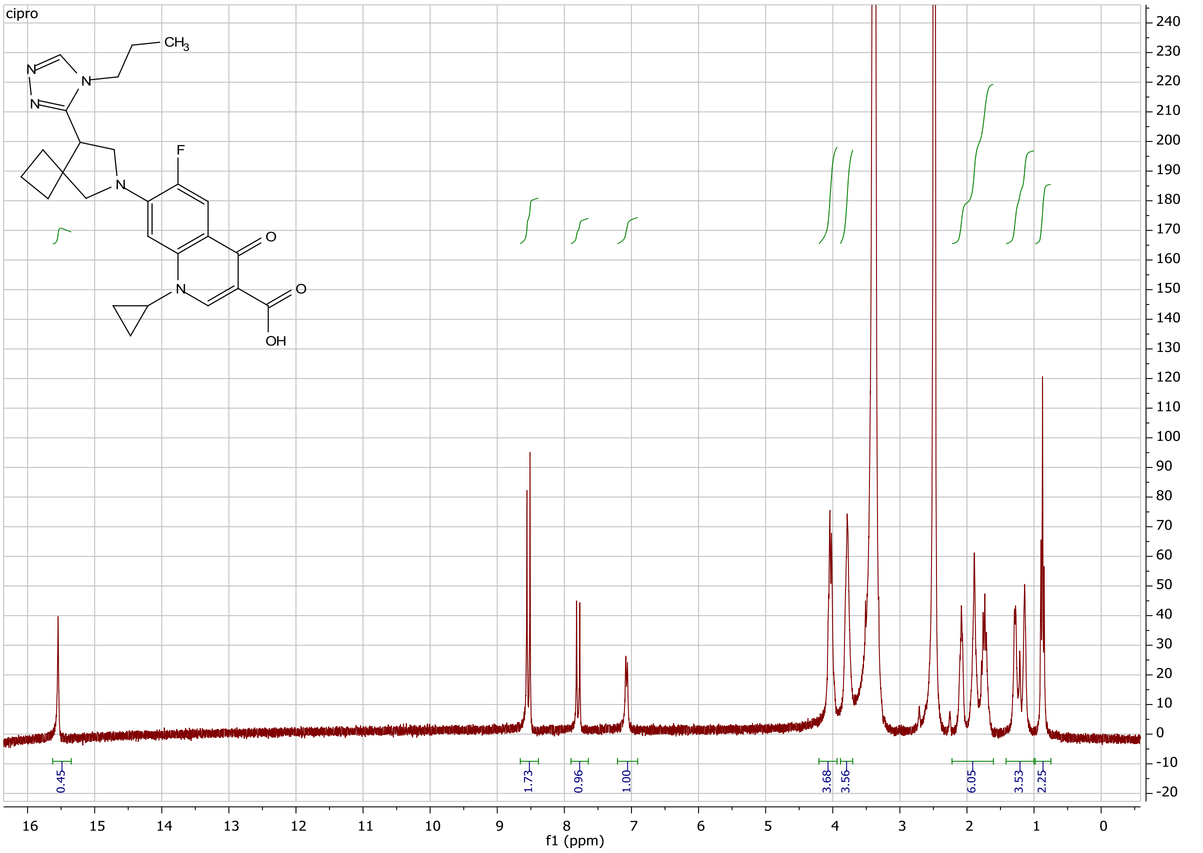

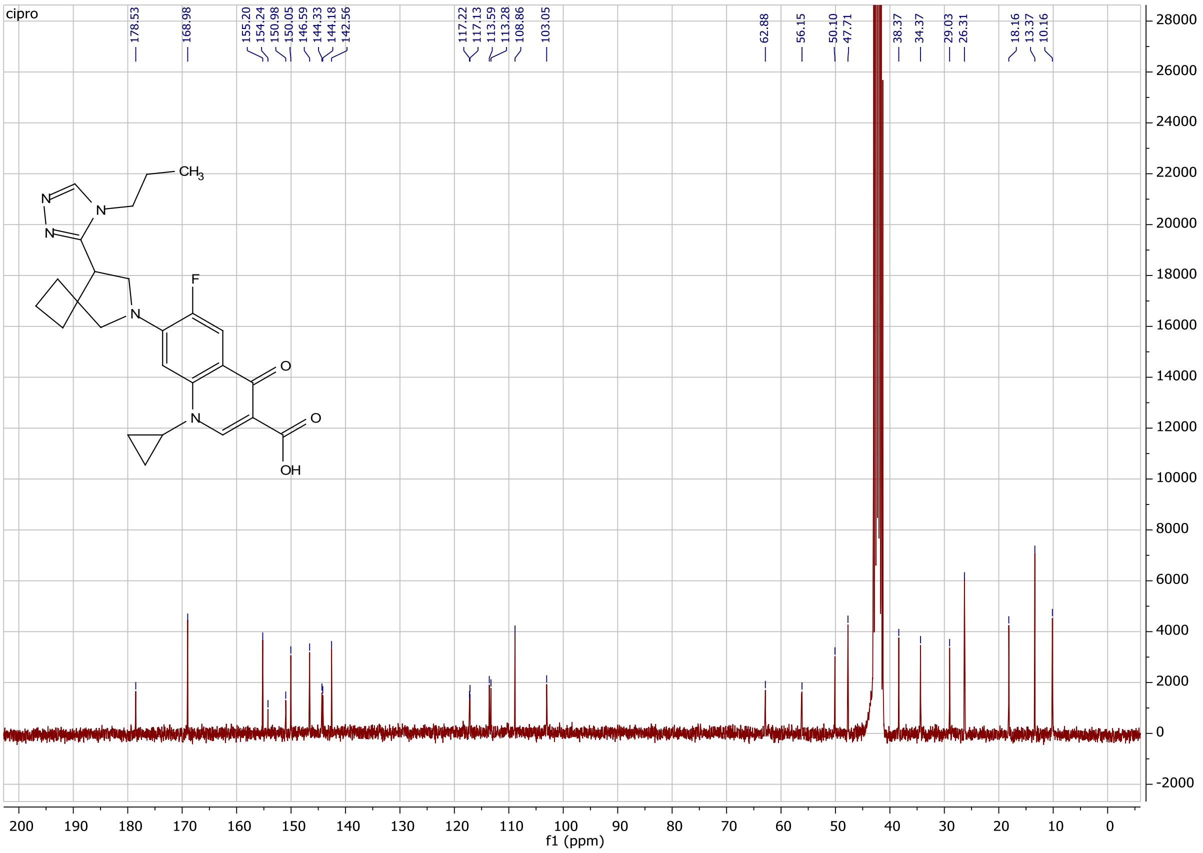

^{1}H and ^{13}C NMR spectra of compound **6c**

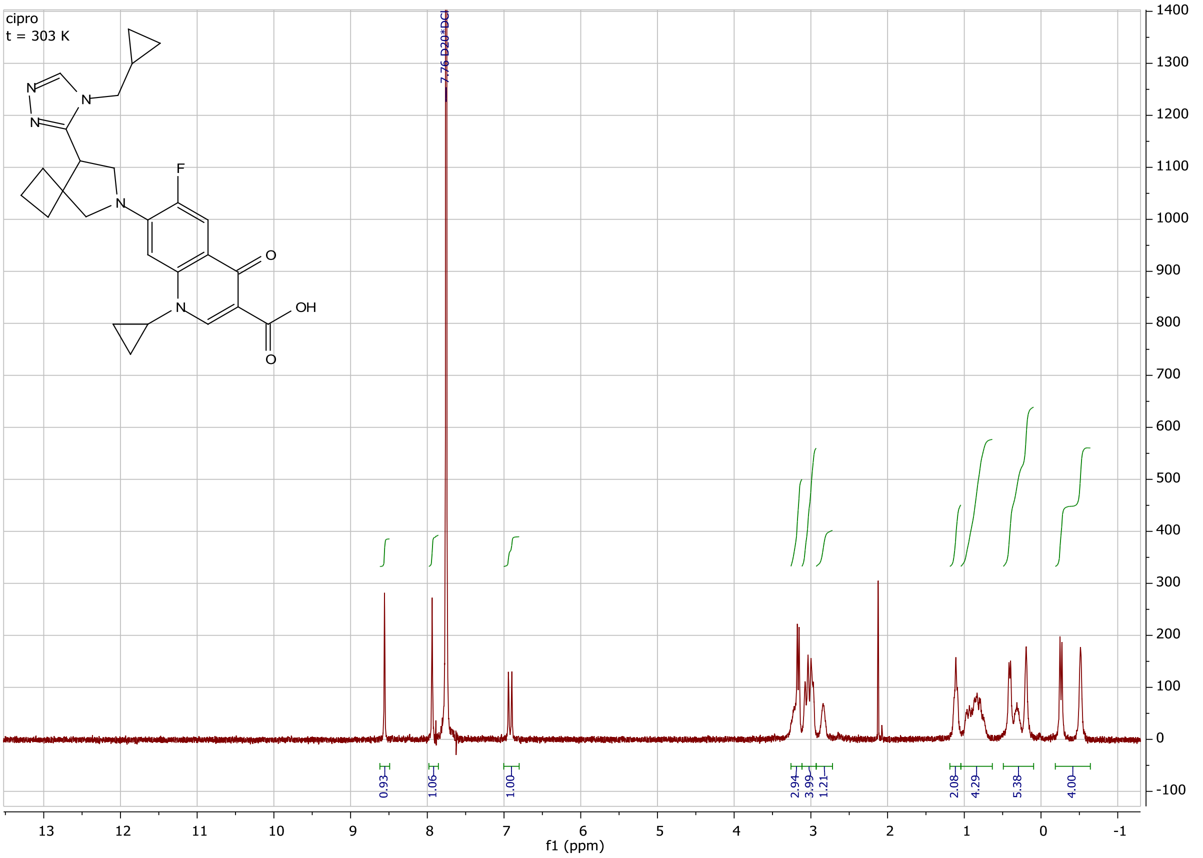

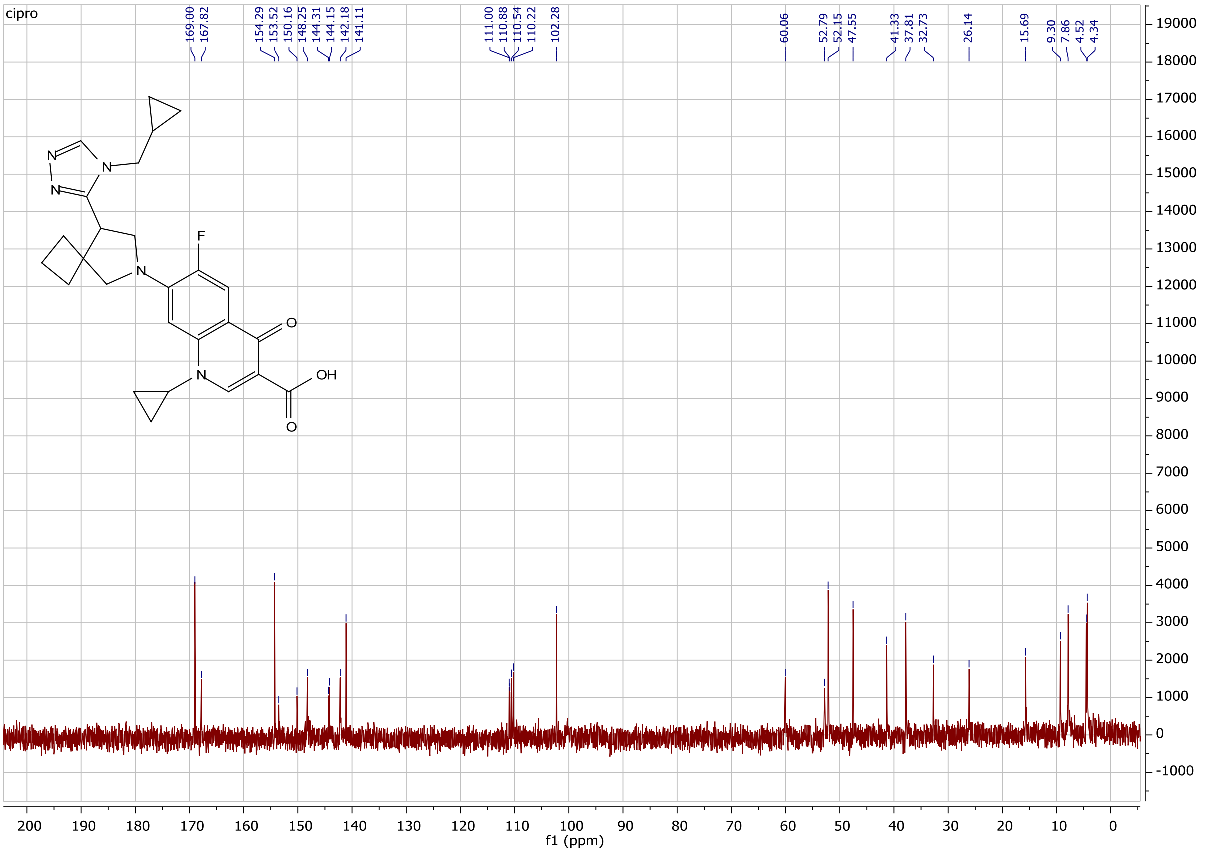

^{1}H and ^{13}C NMR spectra of compound **6d**

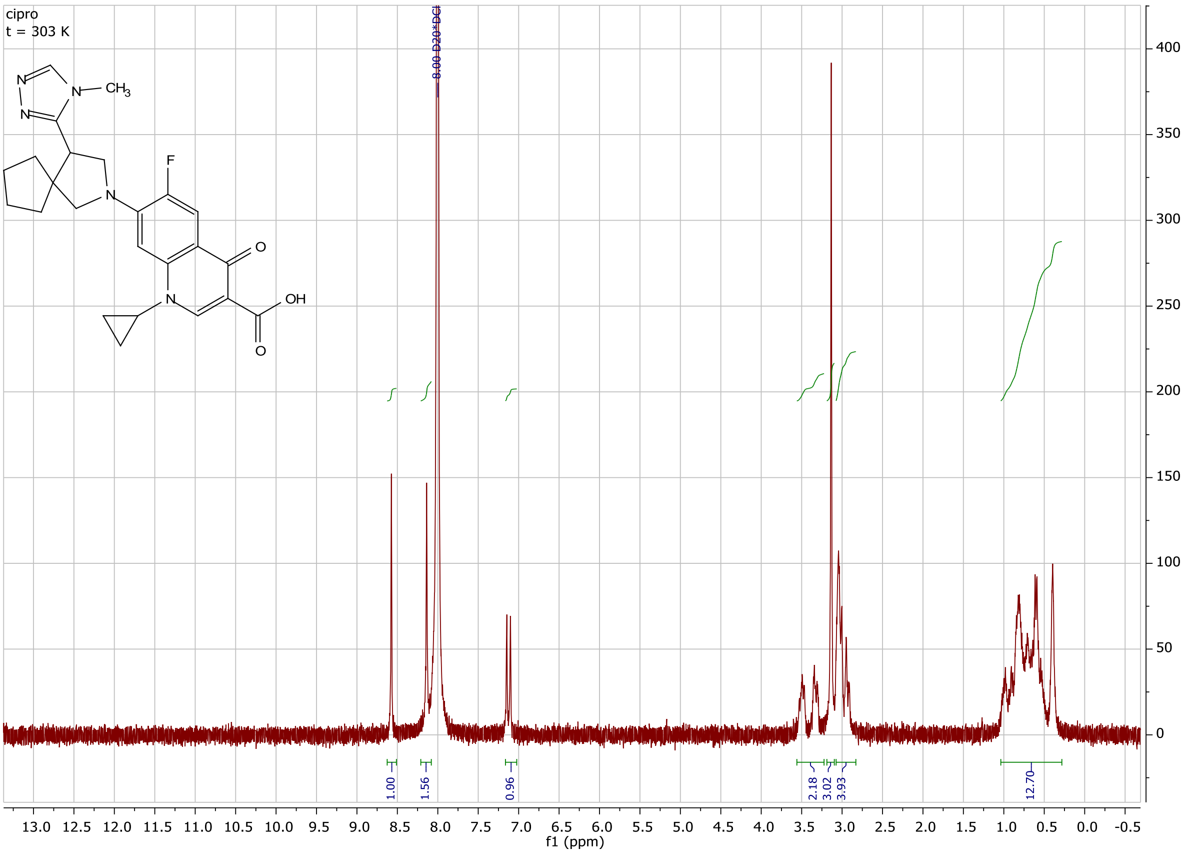

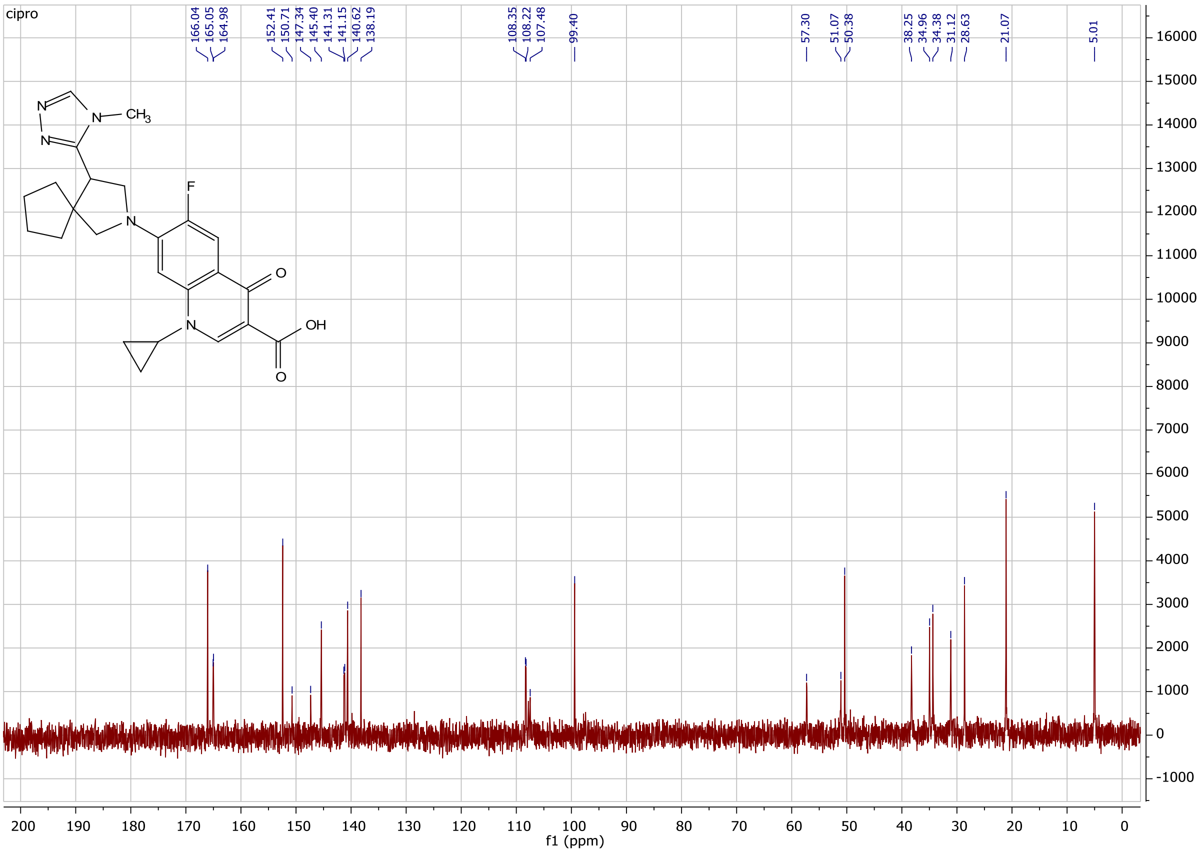

^{1}H and ^{13}C NMR spectra of compound **6e**

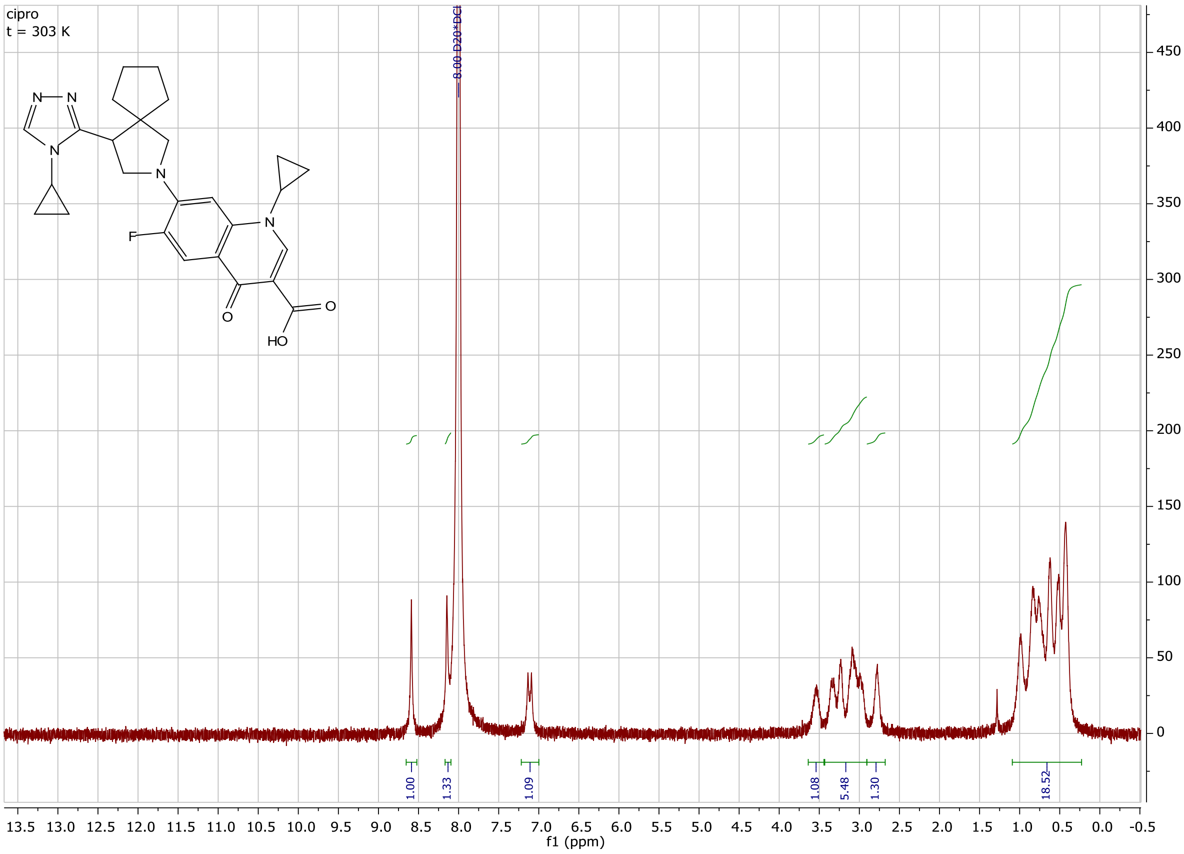

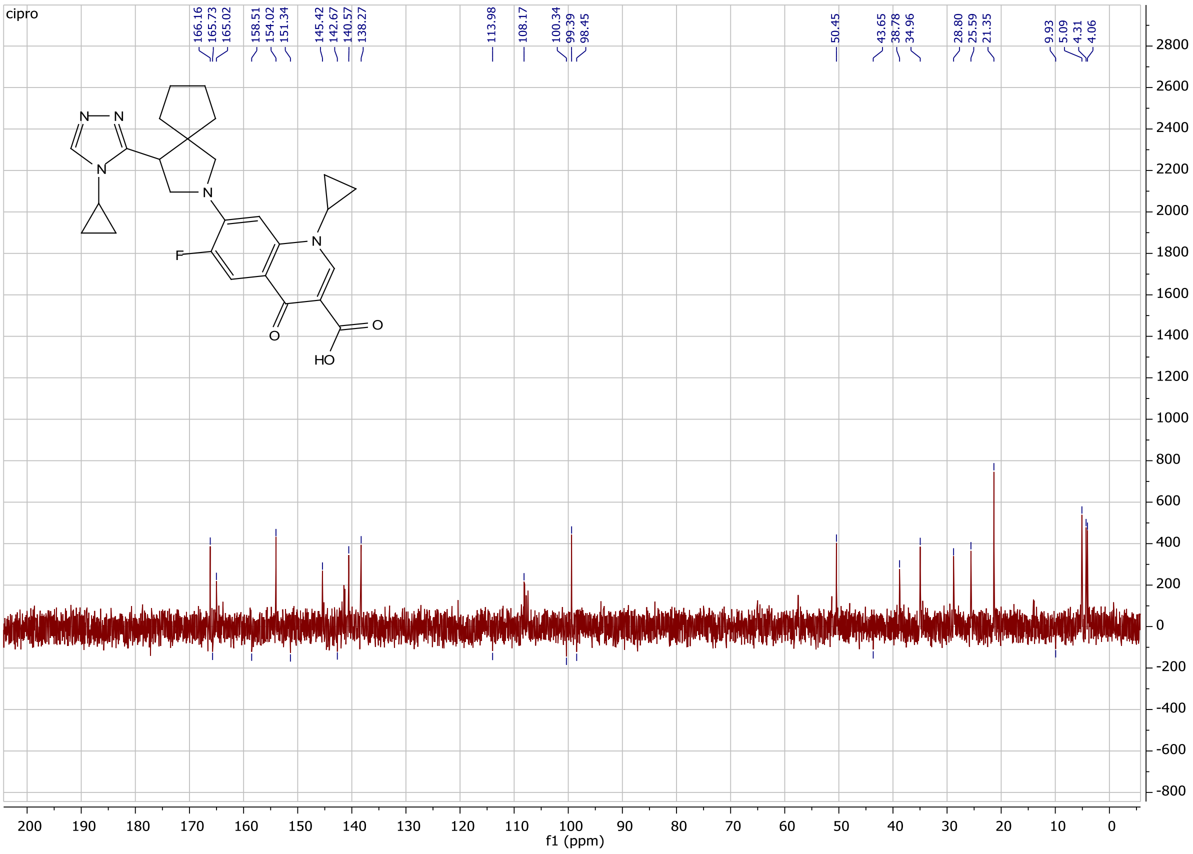

^{1}H and ^{13}C NMR spectra of compound **6f**

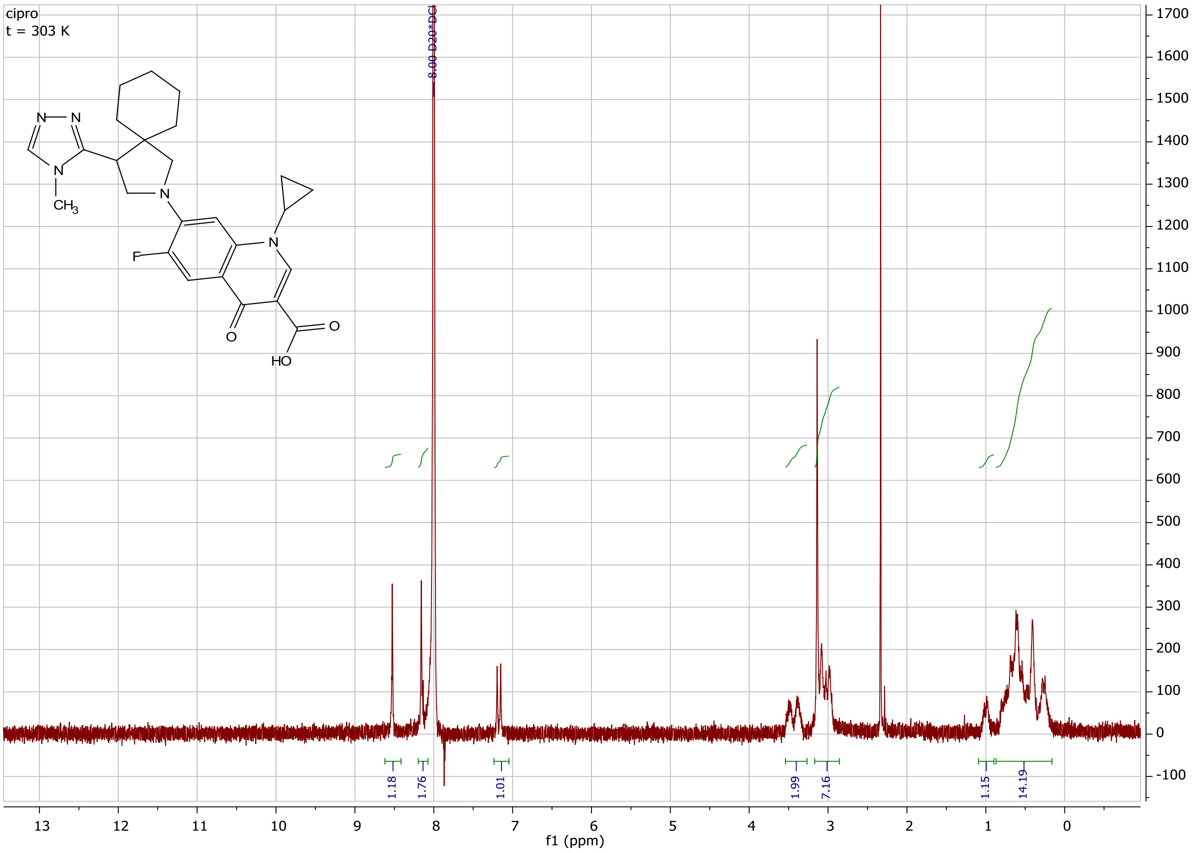

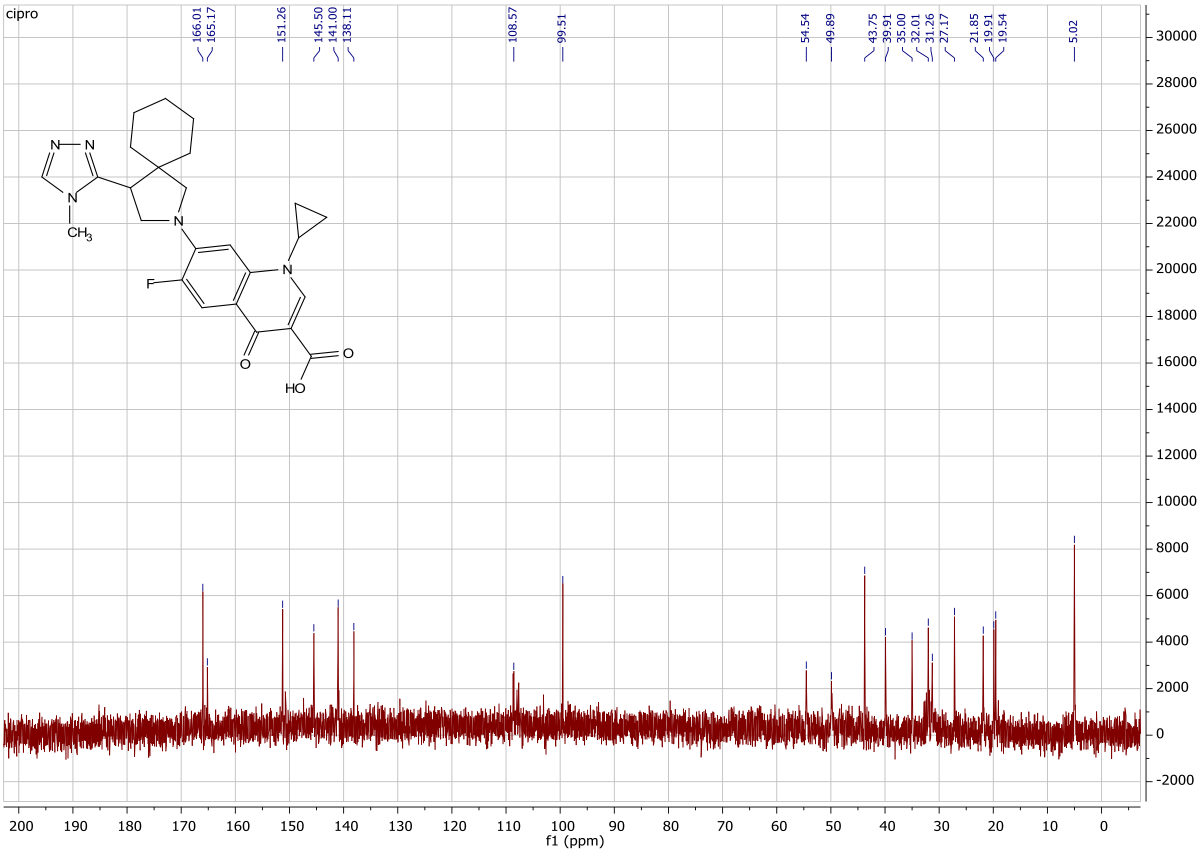

^{1}H and ^{13}C NMR spectra of compound **6g**

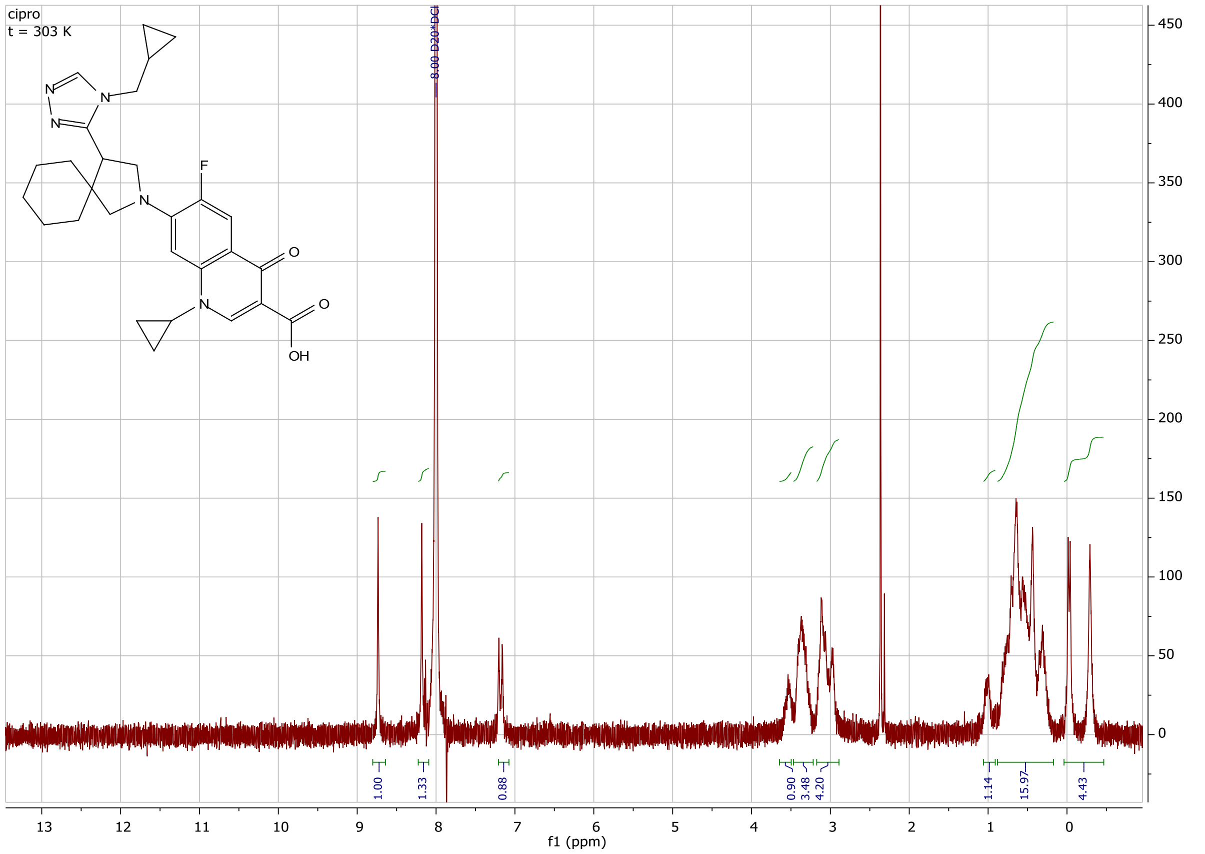

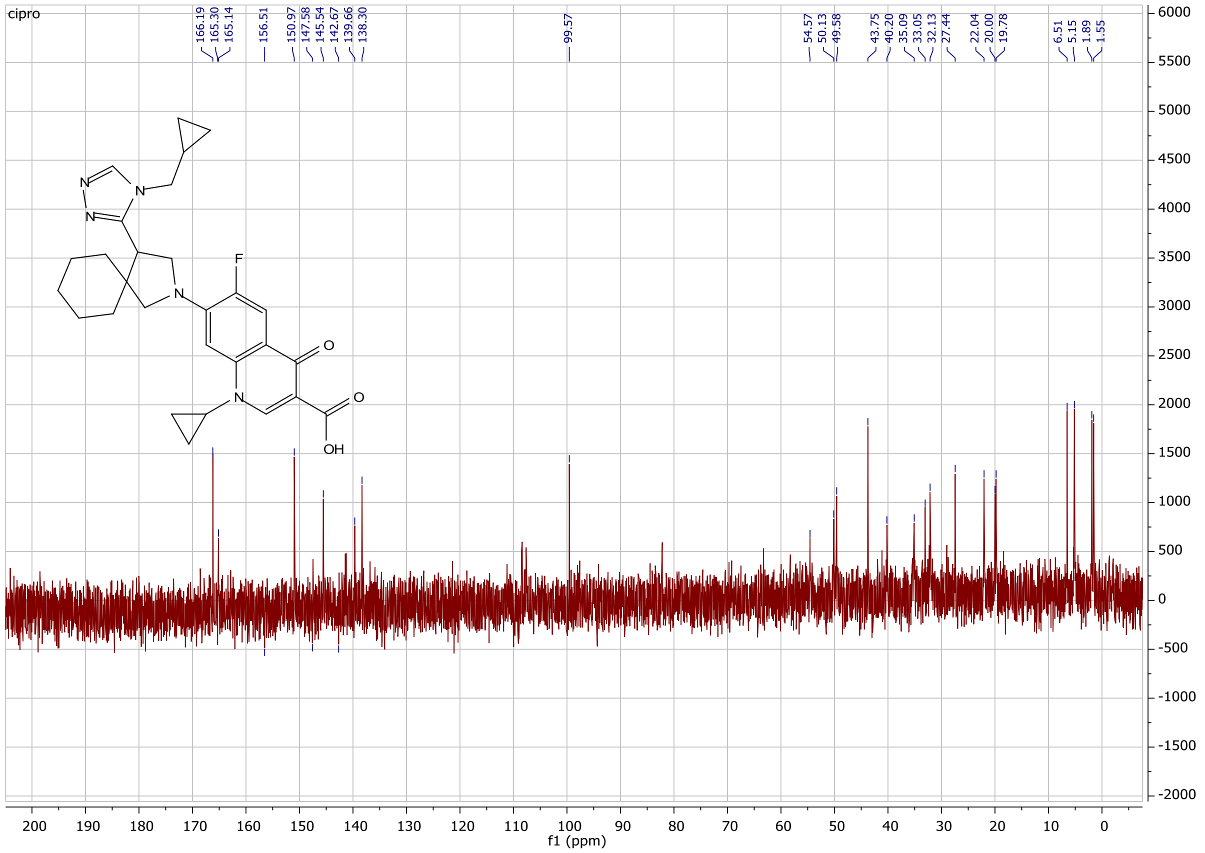

^{1}H and ^{13}C NMR spectra of compound **6h**

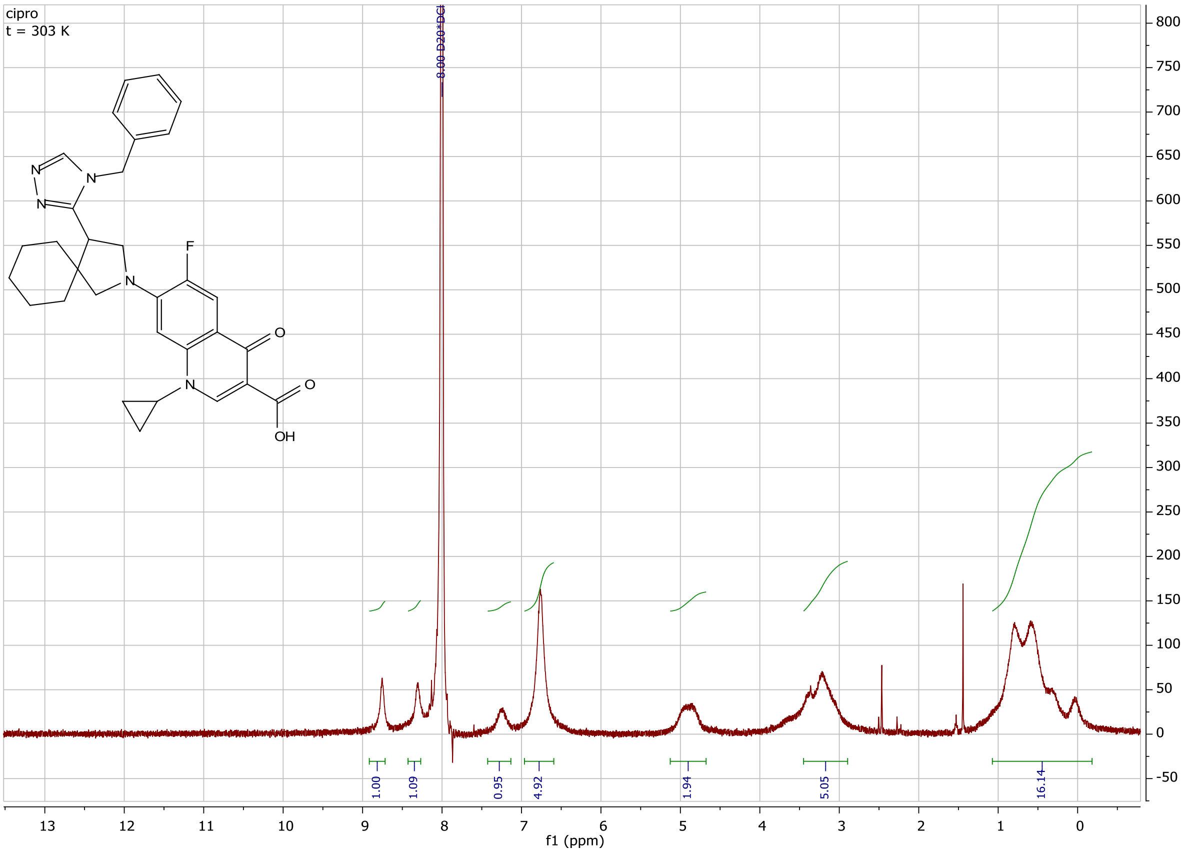

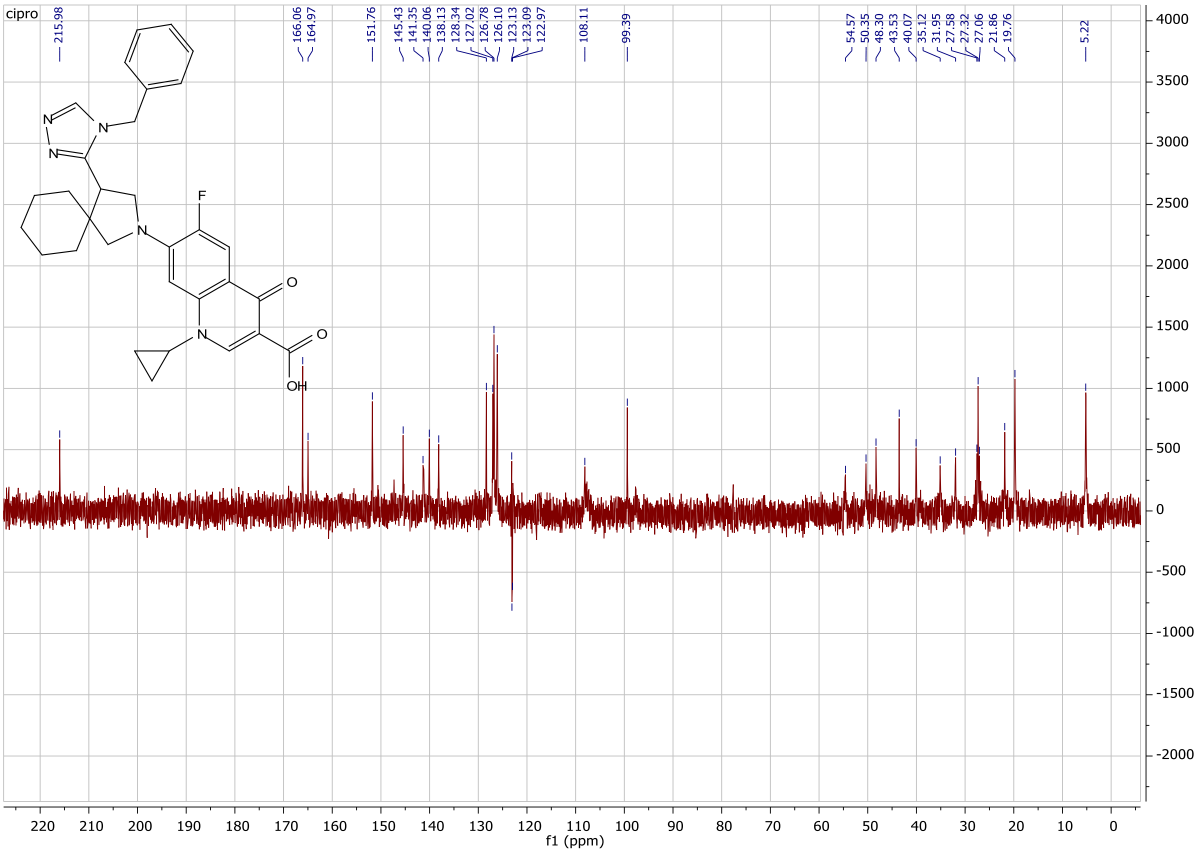

^{1}H and ^{13}C NMR spectra of compound **6i**

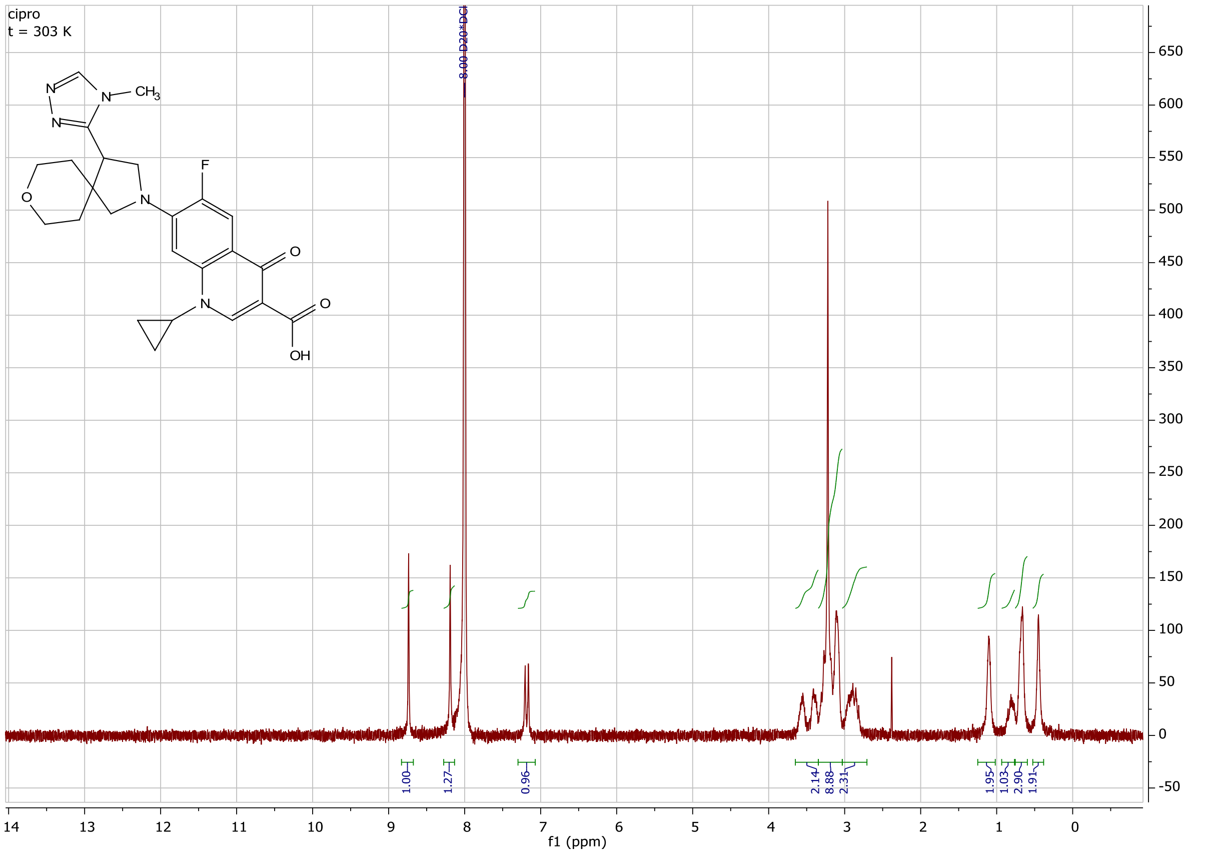

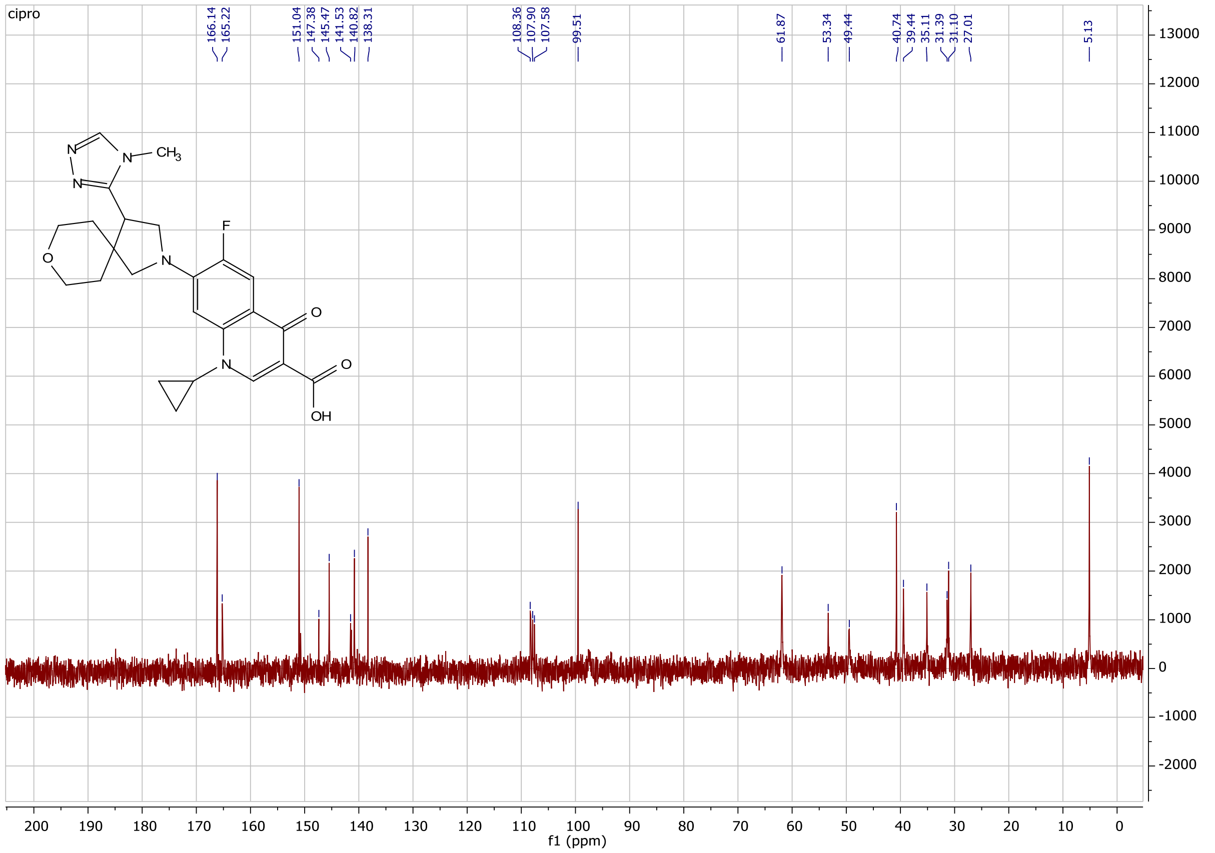

^{1}H and ^{13}C NMR spectra of compound **6j**

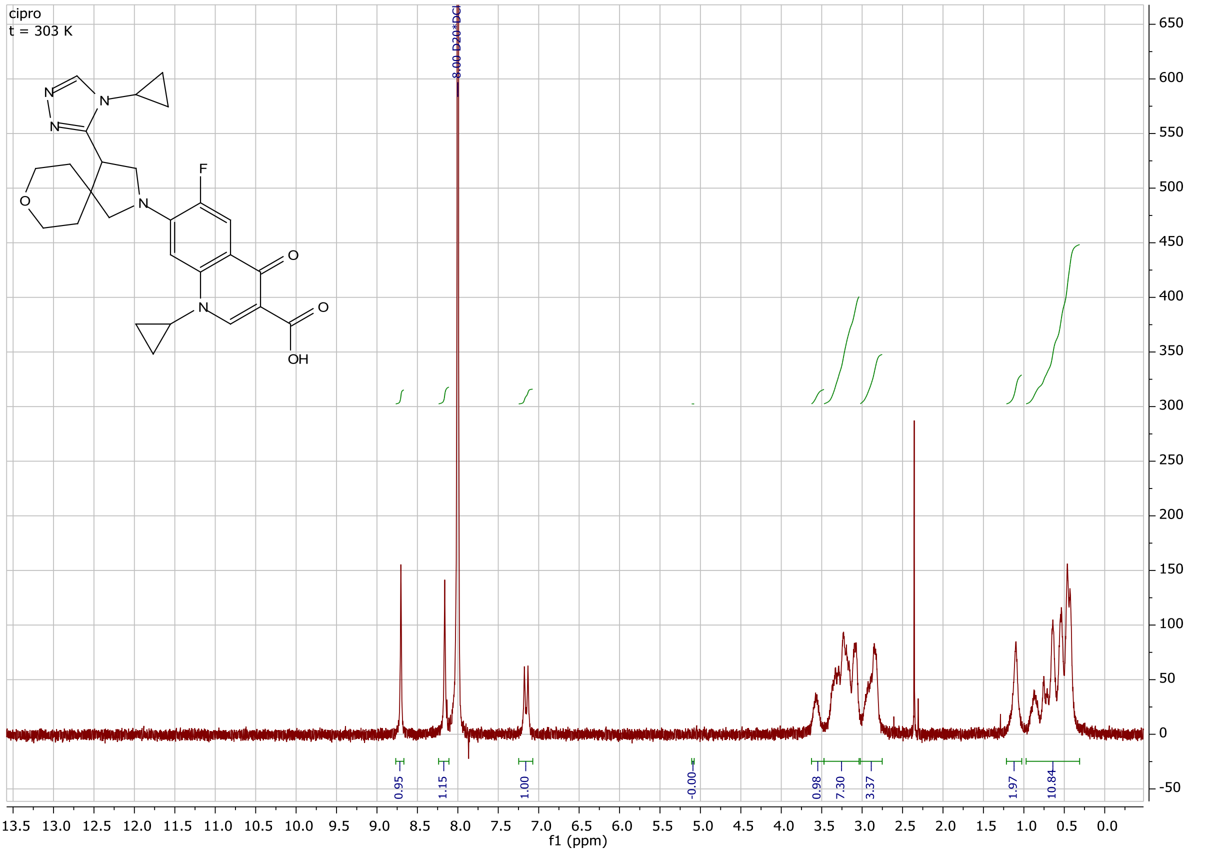

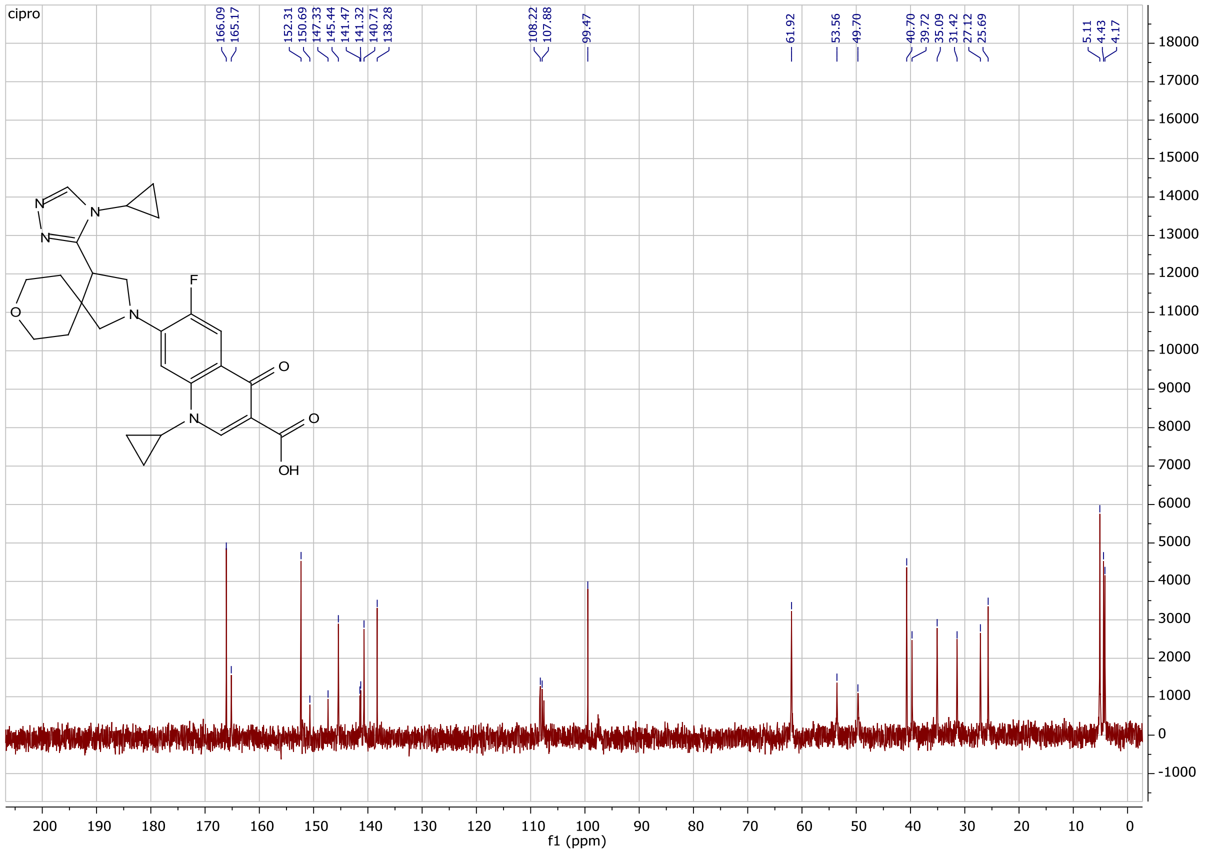

^{1}H and ^{13}C NMR spectra of compound **6k**

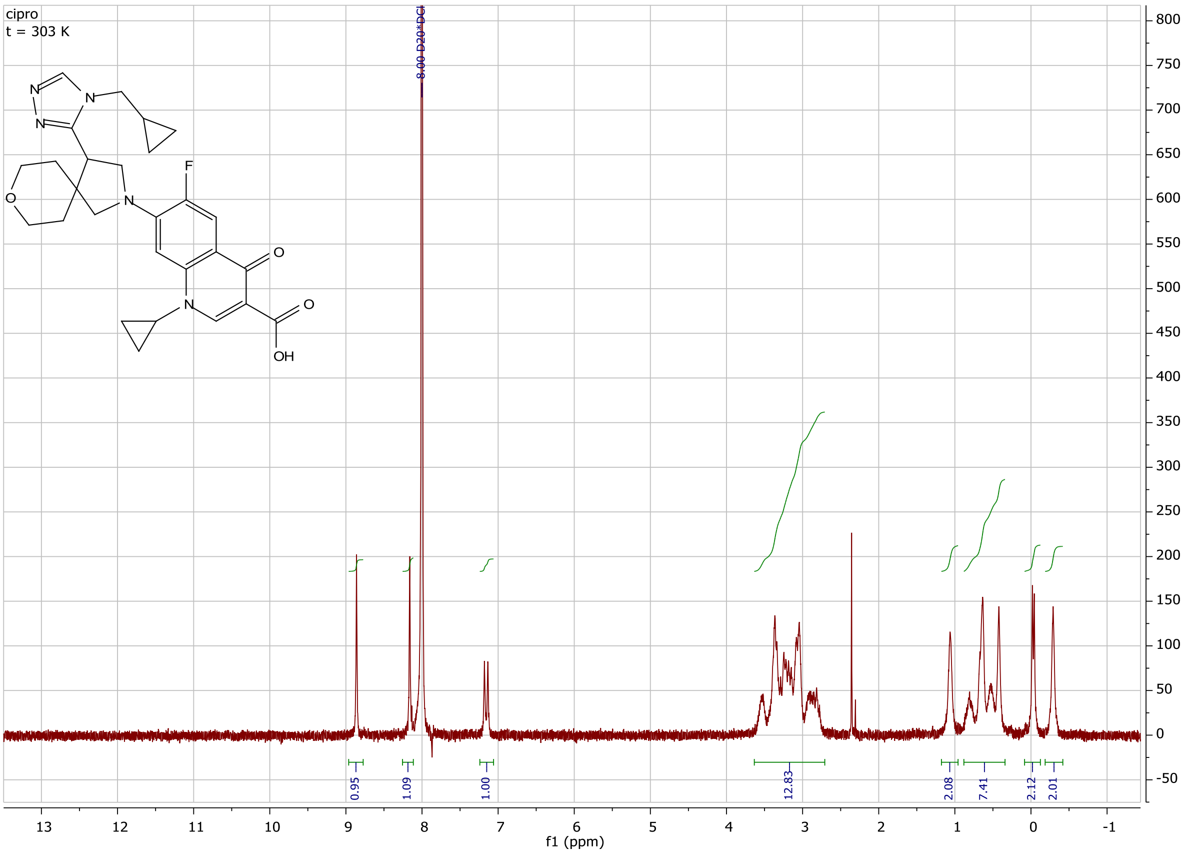

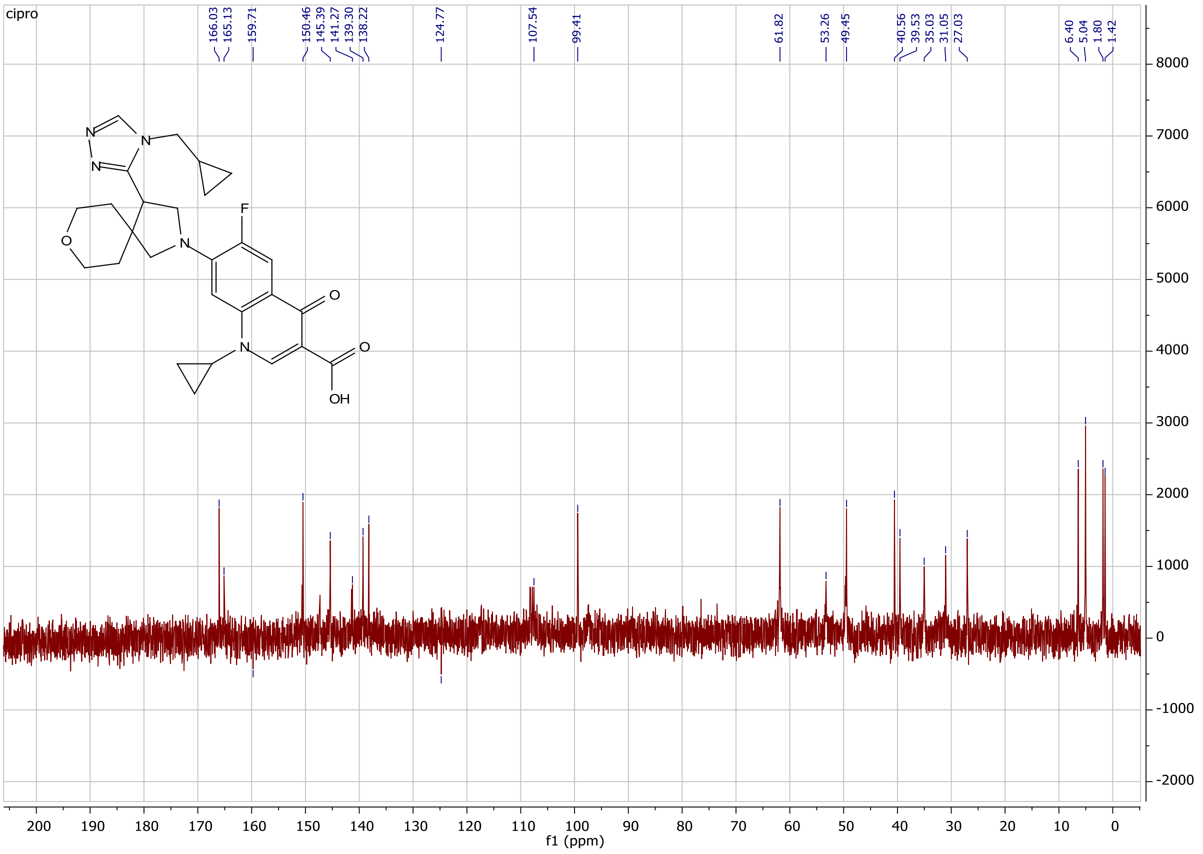

HPLC traces of compounds **6**.

Compound **6a**

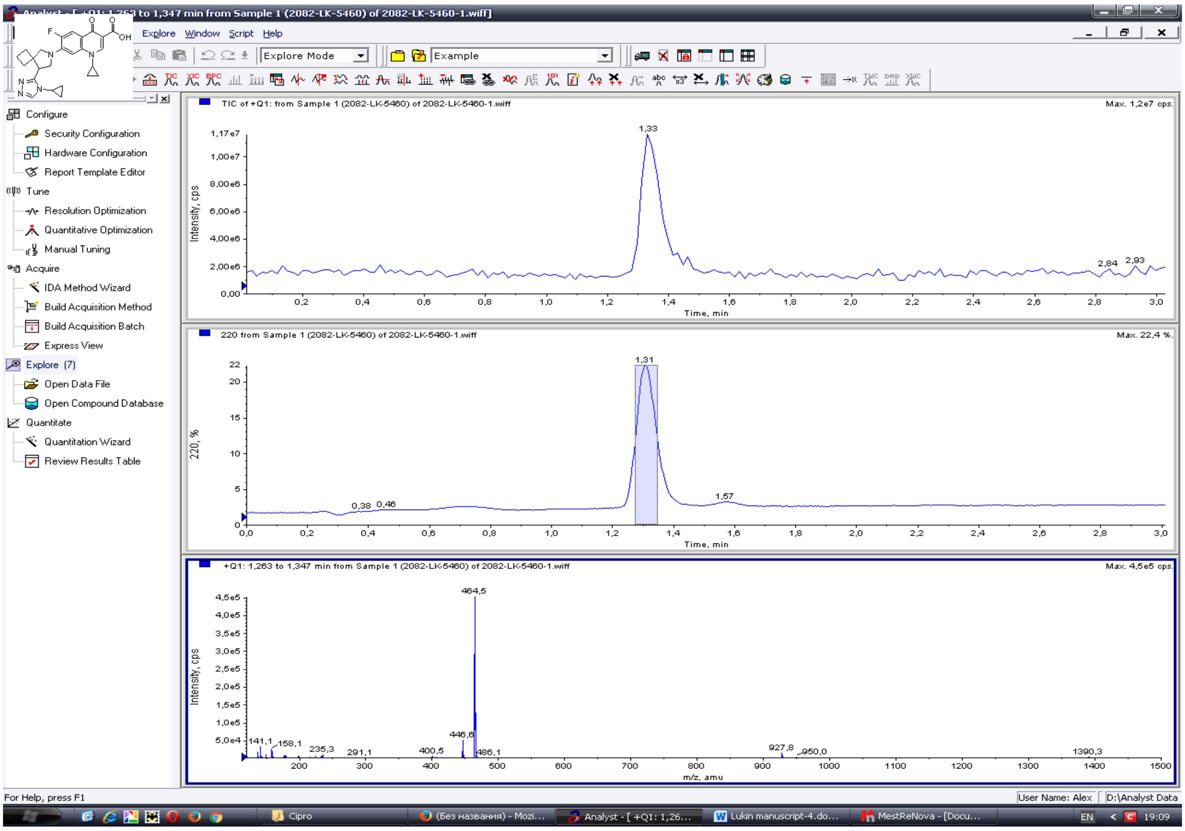

Compound **6b**

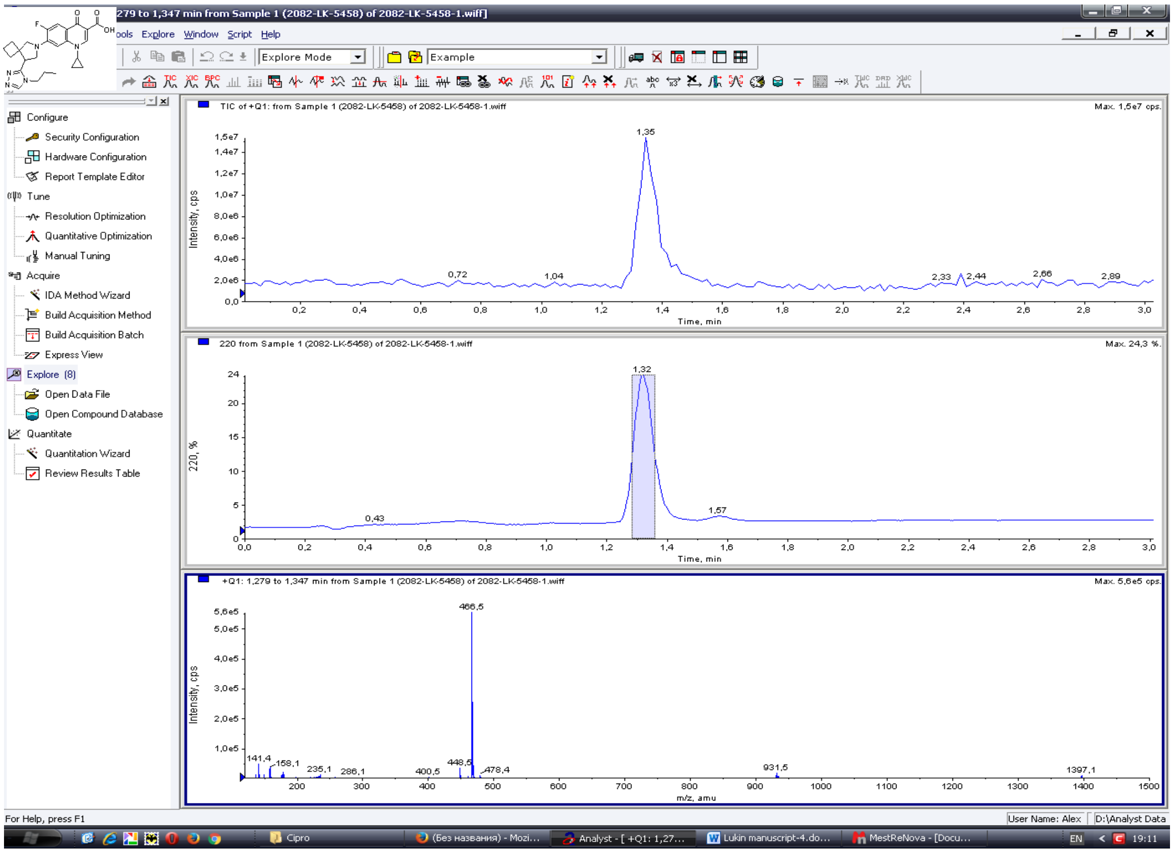

Compound **6c**

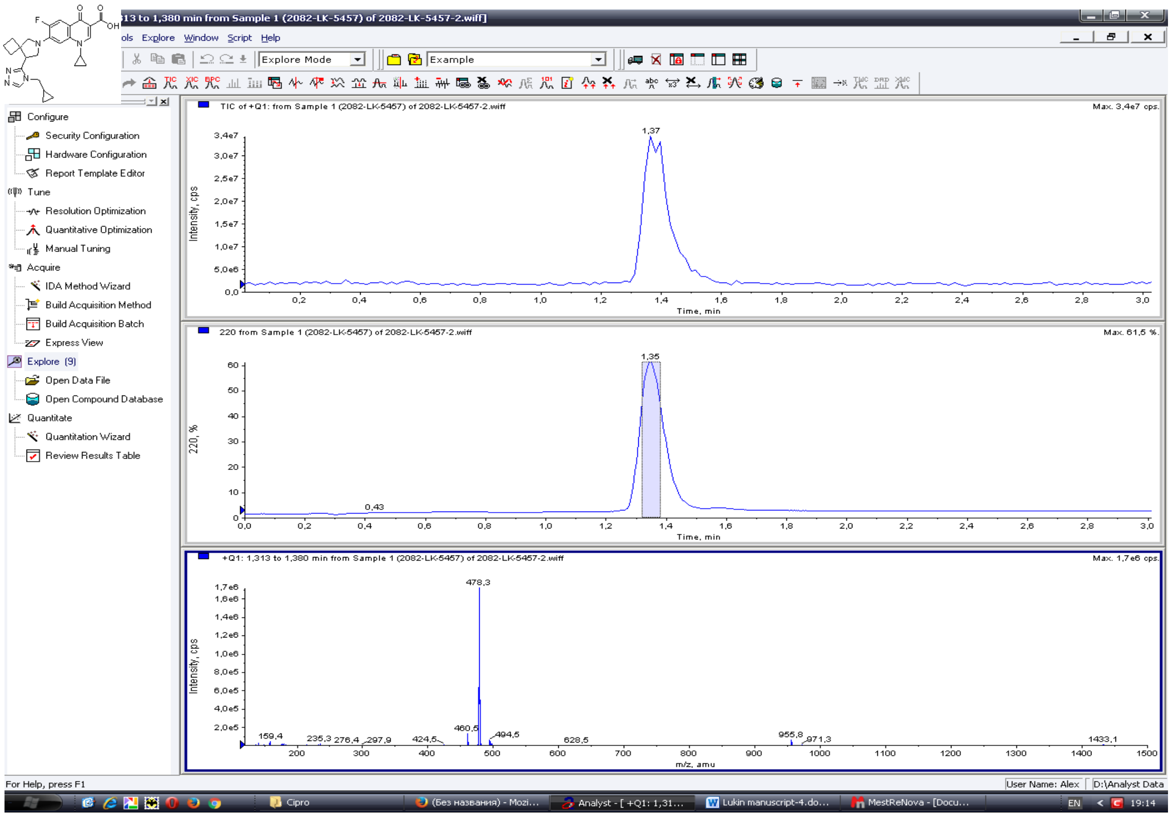

Compound **6d**

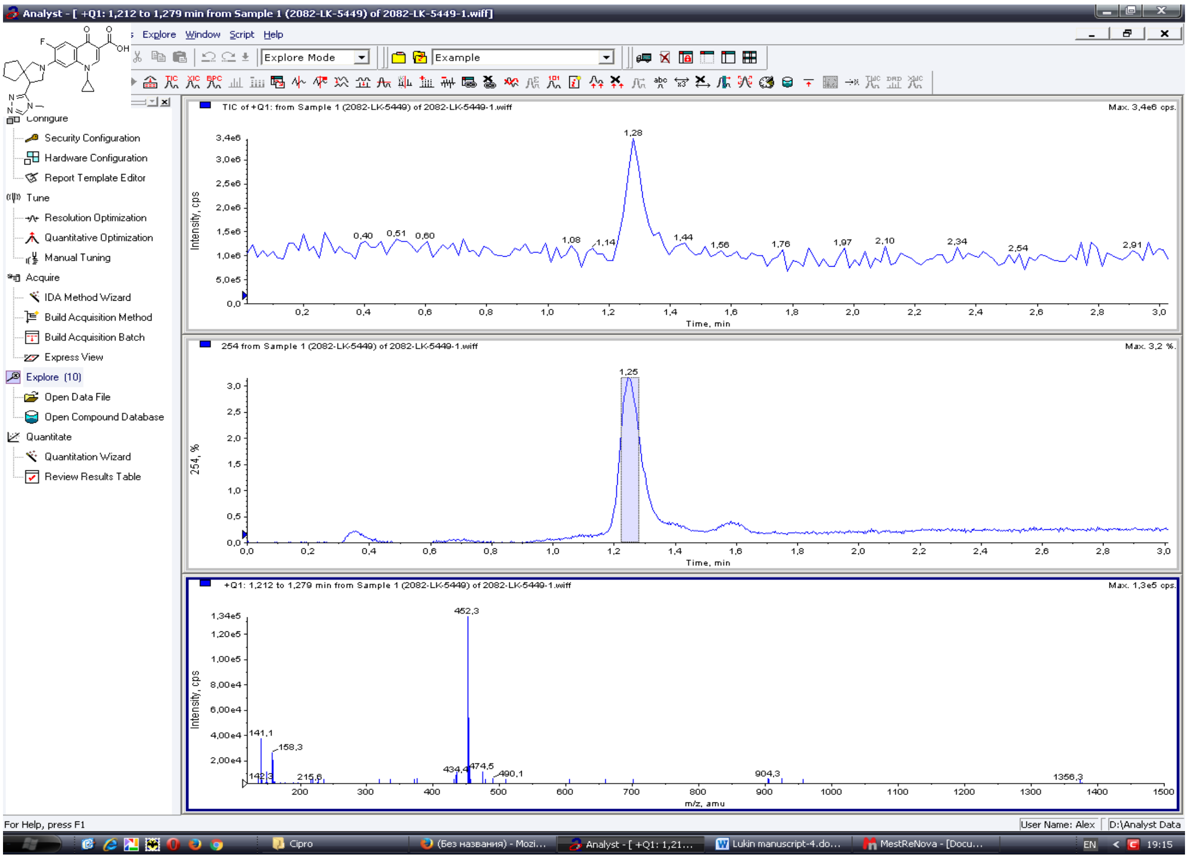

Compound **6e**

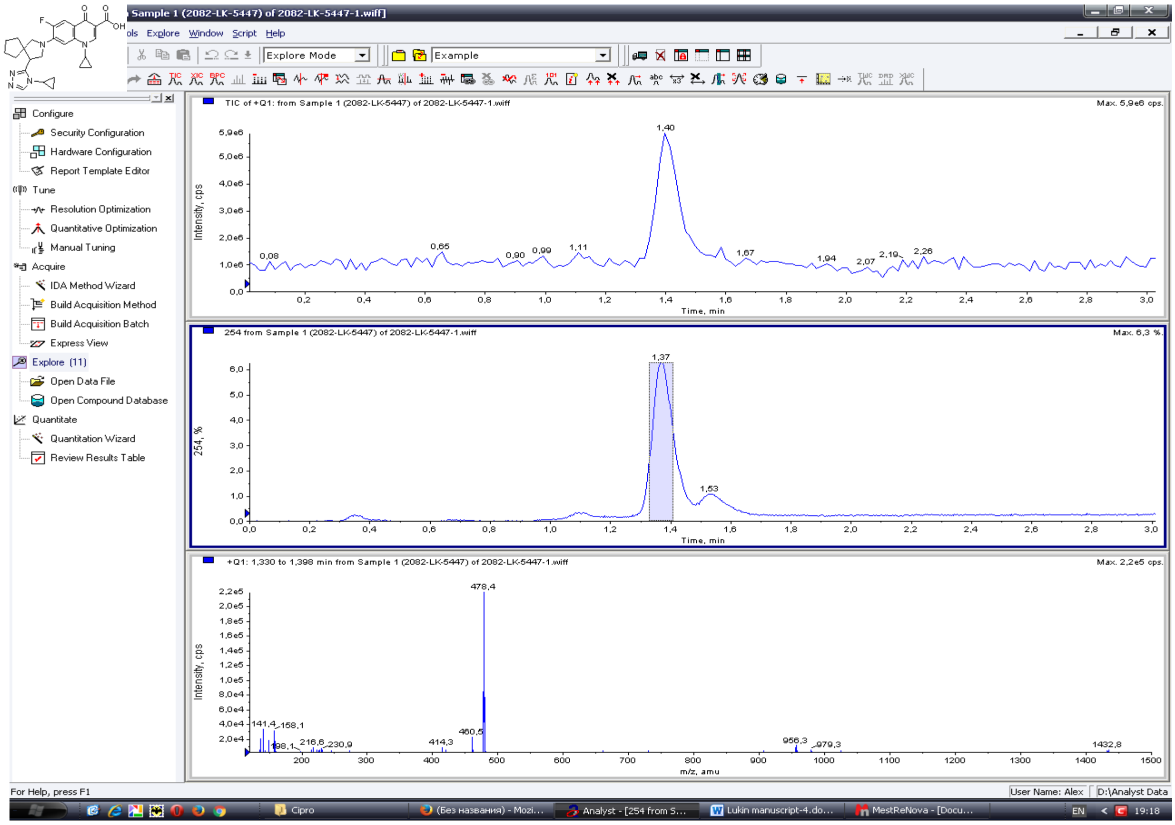

Compound **6f**

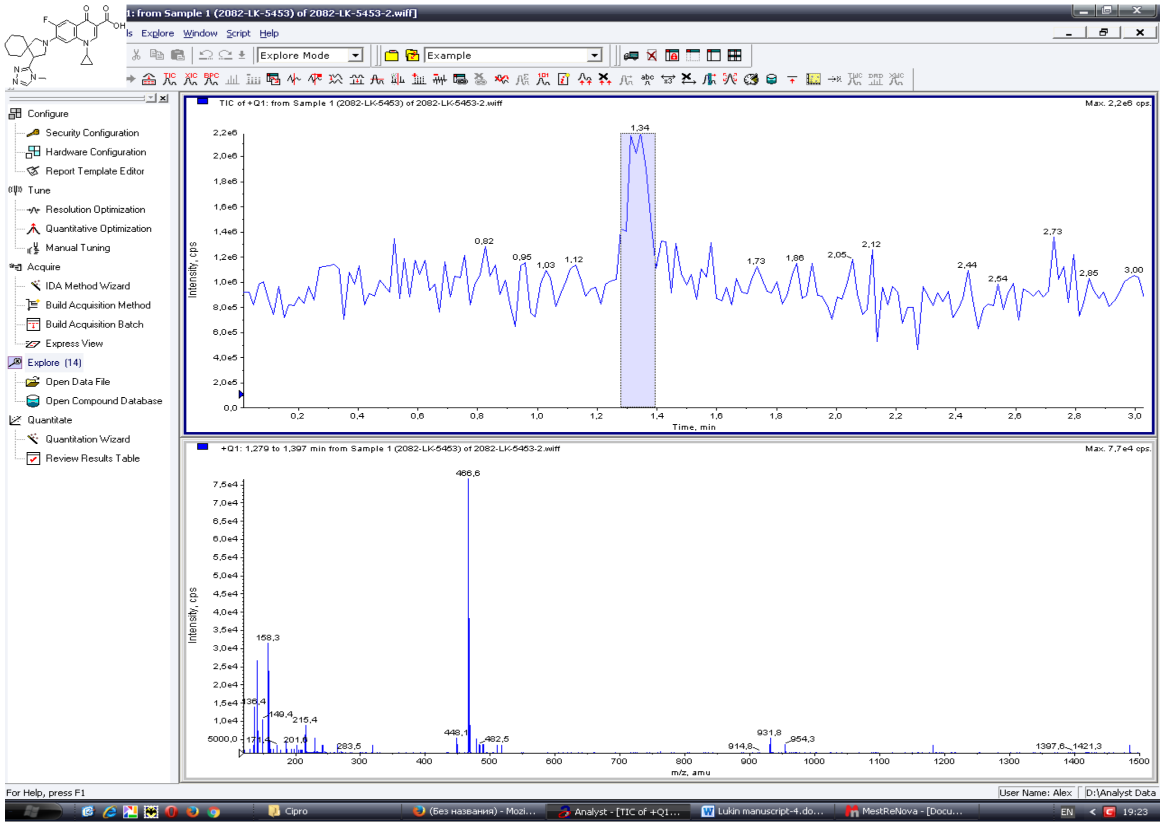

Compound **6g**

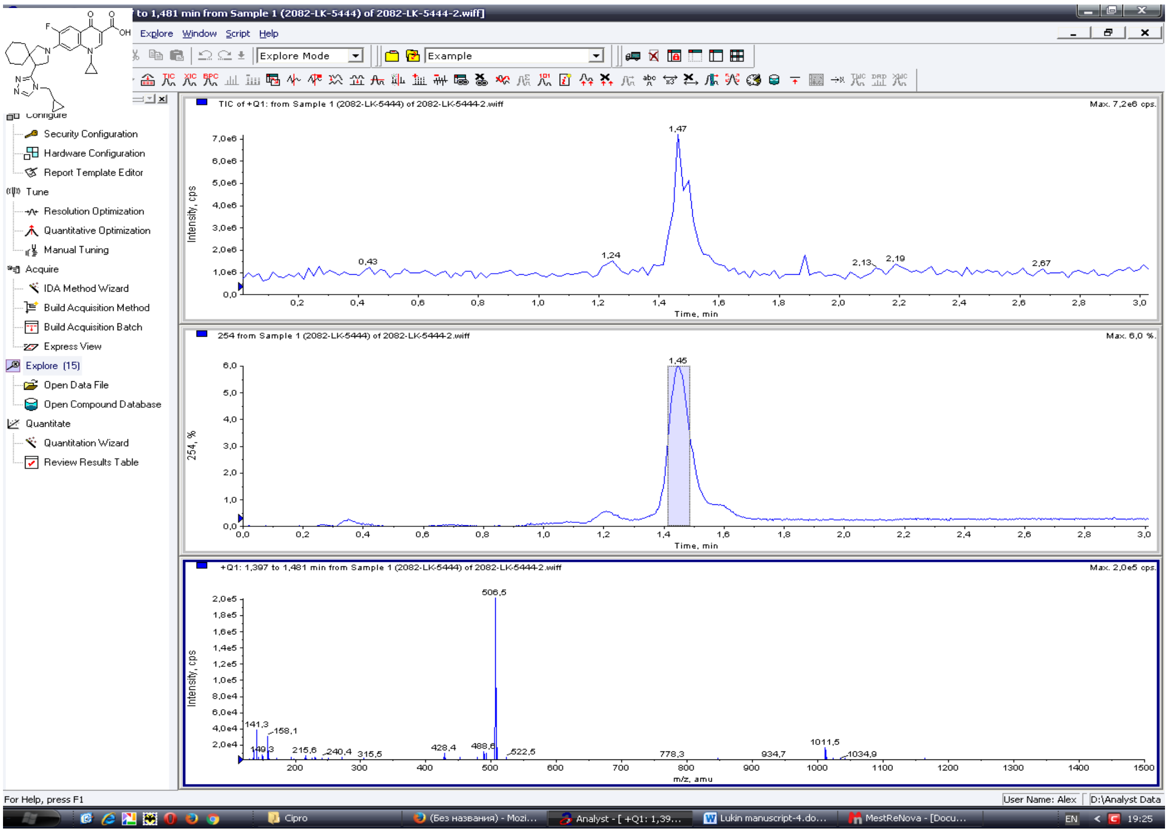

Compound **6h**

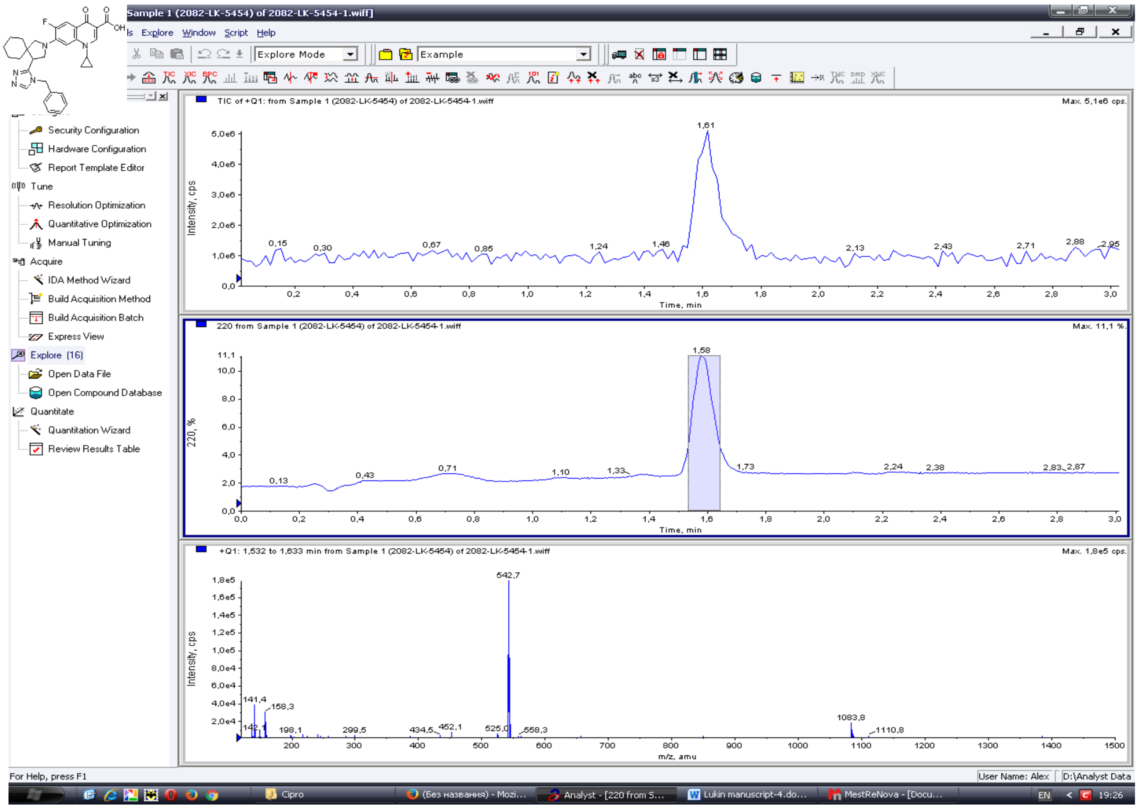

Compound **6i**

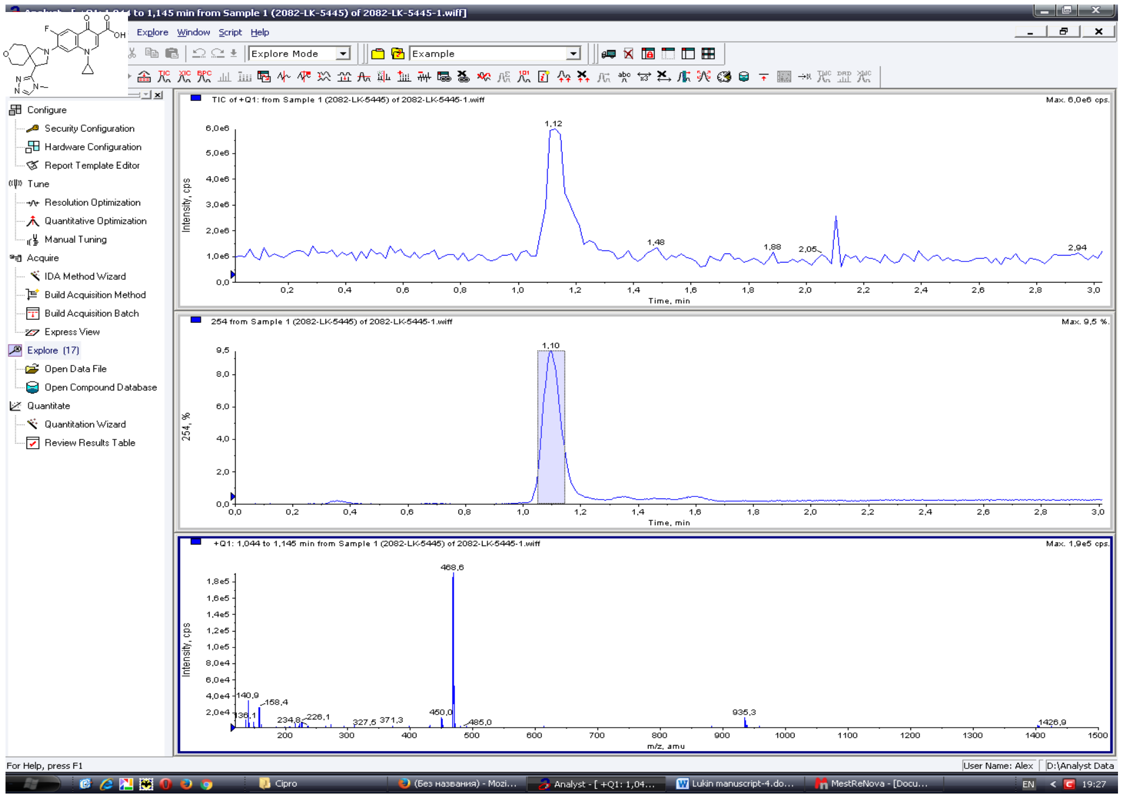

Compound **6j**

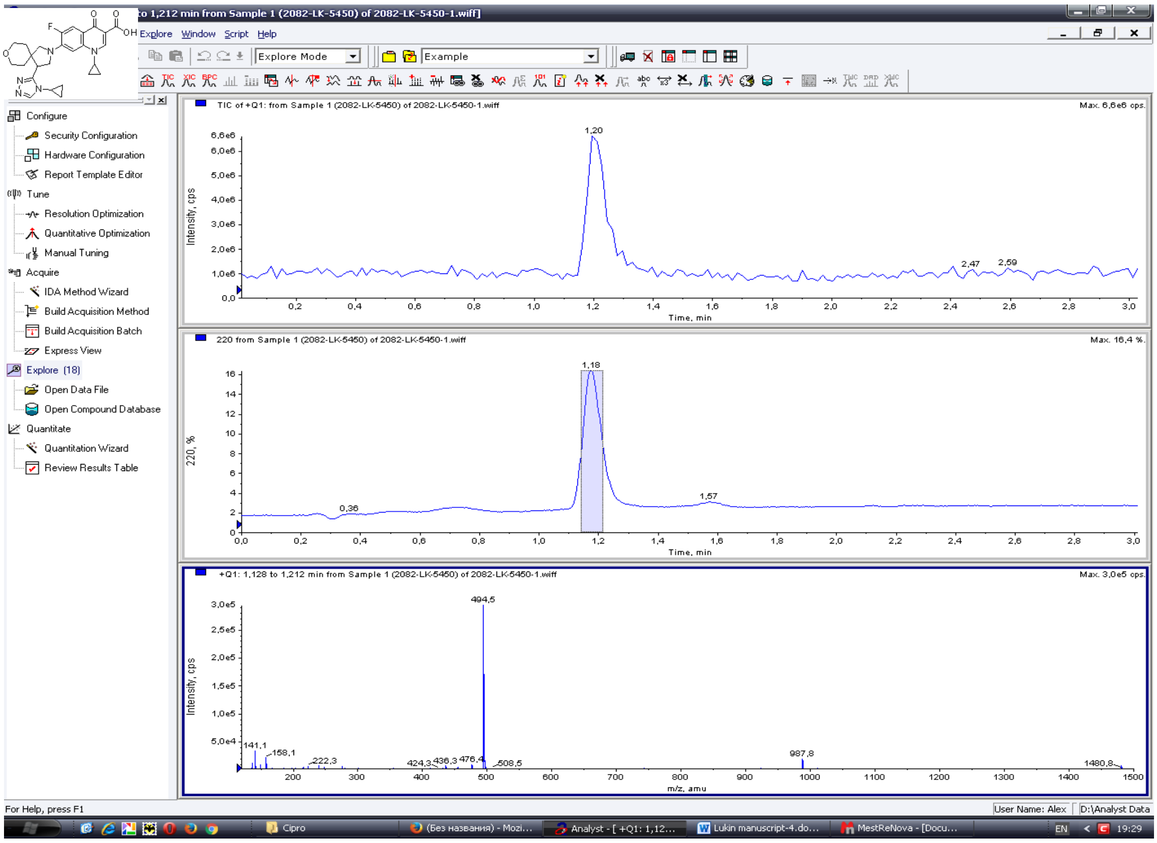

Compound **6k**

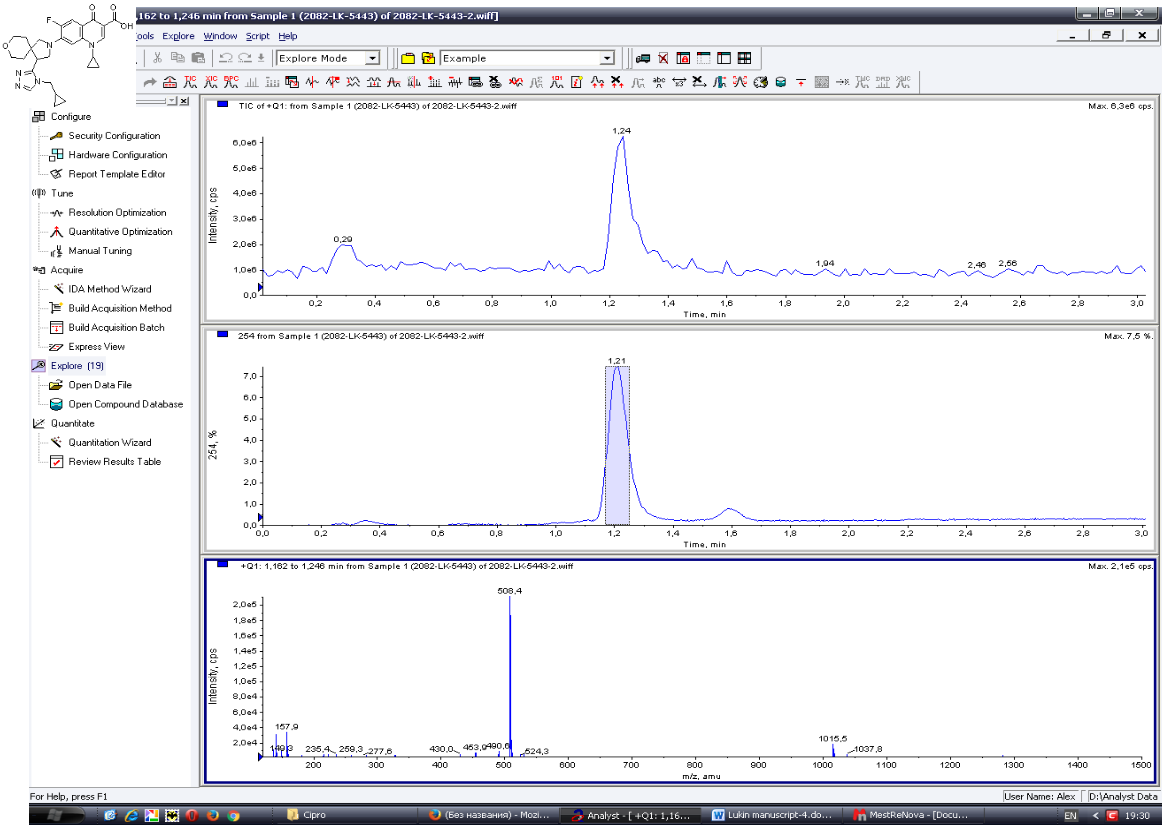

TIC of +Q1: from Sample 1 (2082-LK-5443) of 2082-LK-5443-2.wiff
Max. 6,3e6 cps.
Intensity, cps
Time, min
254 from Sample 1 (2082-LK-5443) of 2082-LK-5443-2.wiff
Max. 7,5 %.
254, %
+Q1: 1,162 to 1,246 min from Sample 1 (2082-LK-5443) of 2082-LK-5443-2.wiff
Max. 2,1e5 cps.
508,4
m/z, amu
